# Adaptive self-assembly and induced-fit transformations of anion-binding metal-organic macrocycles

**DOI:** 10.1038/ncomms15898

**Published:** 2017-06-16

**Authors:** Ting Zhang, Li-Peng Zhou, Xiao-Qing Guo, Li-Xuan Cai, Qing-Fu Sun

**Affiliations:** 1 State Key Laboratory of Structural Chemistry, Fujian Institute of Research on the Structure of Matter, Chinese Academy of Sciences, Fuzhou 350002, China; 2 College of Chemistry, Fuzhou University, Fuzhou 350108, China

## Abstract

Container-molecules are attractive to chemists due to their unique structural characteristics comparable to enzymes and receptors in nature. We report here a family of artificial self-assembled macrocyclic containers that feature induced-fit transformations in response to different anionic guests. Five metal-organic macrocycles with empirical formula of M_*n*_L_2*n*_ (M=Metal; L=Ligand; *n*=3, 4, 5, 6, 7) are selectively obtained starting from one simple benzimidazole-based ligand and square-planar palladium(II) ions, either by direct anion-adaptive self-assembly or induced-fit transformations. Hydrogen-bonding interactions between the inner surface of the macrocycles and the anionic guests dictate the shape and size of the product. A comprehensive induced-fit transformation map across all the M_*n*_L_2*n*_ species is drawn, with a representative reconstitution process from Pd_7_L_14_ to Pd_3_L_6_ traced in detail, revealing a gradual ring-shrinking mechanism. We envisage that these macrocyclic molecules with adjustable well-defined hydrogen-bonding pockets will find wide applications in molecular sensing or catalysis.

## Abstract (web summary)

Container-molecules capable of recognizing charged species possess great potential as sensors, but are typically limited by their rigid frameworks. Here, Sun and co-workers design a family of adaptive metal-organic macrocycles that exhibit shape and size induced-fit transformations upon anion-binding.

Coordination-driven self-assembly has become one of the most convenient strategies for the bottom-up construction of functional molecular ensembles^1^,^2^,^3^,^4^,^5^. On the basis of elaborate symmetry considerations, numerous two-dimensional (2D) and three-dimensional (3D) architectures have been obtained in ease by the simple combination of designed ligands (L) and metal ions (M), which show great potential in the modulation of reactivity and/or photoelectric properties of guest molecules by encapsulation^6^,^7^,^8^,^9^,^10^. To ensure the directed assembly, rigid ligands are most often employed^5^,^11^,^12^. Flexible ligands, which usually give rise to interconvertible architectures, are far less utilized in coordination-directed self-assembly^13^. However, assemblies available in nature favour flexibility over rigidity. For example, enzymes are rather flexible structures, the active site of which is continuously reshaped by interactions with specific substrates, following the so called induced-fit mechanism^14^.

Anion receptor chemistry has witnessed great advances in the past decades. This area of supramolecular chemistry has a number of potential applications in biology, environment and the food industry^15^,^16^,^17^. The fast developing coordination-driven supramolecular chemistry has provided enormous examples of metal-organic assembled systems that can recognize, respond to, or sense negative-charged species^18^,^19^,^20^,^21^,^22^. However, most metal-organic receptors reported so far are of invariant structures, aiming to reach high selectivity towards targeting anions. Adaptive anion receptors, that is, receptors that can continuously transform its shape and size to maximize the binding interactions with different anions, remain elusive^23^,^24^,^25^,^26^. For example, seminal work by Hasenknopf *et al*.^23^ has shown that use of flexible tris-bipy ligands and iron(II) lead to the formation of a dynamic combinatorial system^27^,^28^,^29^, where a set of circular helicates is expressed depending on the anions present during the self-assembly process.

Herein, we report an artificial assembly system that features adaptive self-assembly and induced-fit transformation properties in the presence of anionic guests. A family of metal-organic macrocycles with the general formula of Pd_*n*_L_2*n*_ (*n*=3, 4, 5, 6, 7) are selectively obtained starting from one simple ligand and square-planar Pd^II^ ions. Hydrogen bonding between the inner surface of the macrocycles and the bound guests, different anions in this case, dictates the shape and size of the final product. *In situ* anion-adaptive self-assembly gives rise to the Pd_*n*_L_2*n*_ species for *n*=3, 6, 7. For *n*=4, 5, post-synthetic transformations^26^,^30^,^31^,^32^,^33^,^34^ from other macrocycles are employed, featuring an induced-fit transformation process. Five distinct macrocycles are clearly characterized by NMR, ESI-TOF-MS, and in the case of *n*=3, 4, 5, 6 by single crystal X-ray diffraction. Moreover, a comprehensive map showing all the transformations across the macrocyclic species was drawn, with a representative reconstitution process from Pd_7_L_14_ to Pd_3_L_6_ traced in detail by titration experiments, revealing a gradual ring-shrinking mechanism.

## Results

### Syntheses and characterization of metal-organic macrocycles 2–6

**Figure 1:**
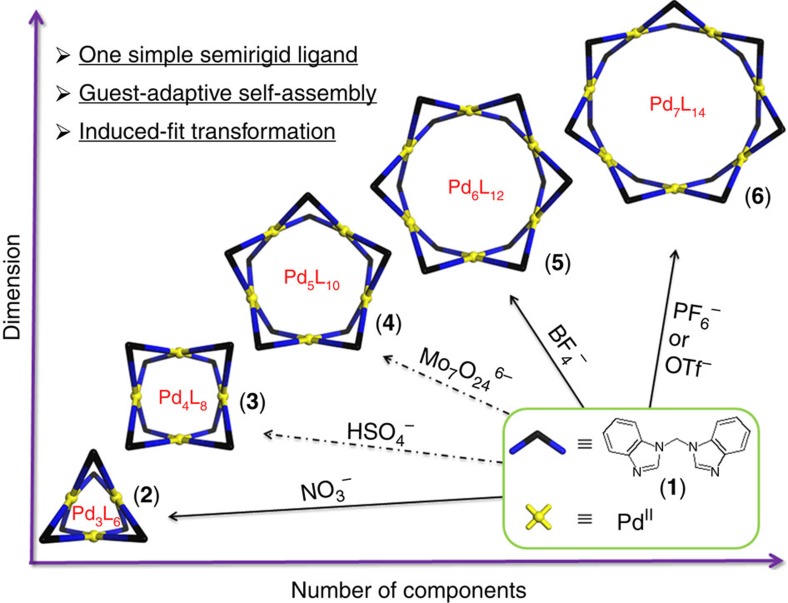
Self-assembly of anion-binding metal-organic macrocycles. Self-assembly of anion-binding metalorganic macrocycles with an empirical formula of Pd_*n*_L_2n_ (*n* = 3, 4, 5, 6, 7). Dashed arrows indicate that Pd_4_L_8_ and Pd_5_L_10_ were obtained by induced-fit transformation from other macrocycles instead of direct self-assembly.

It is well-established that self-assembly of rigid planer bidentate pyridinyl ligands with specific bent angles and square-planar Pd^II^ ions will lead to a group of Pd_*n*_L_2*n*_ molecular spheres^35^,^36^,^37^,^38^,^39^,^40^,^41^,^42^. Considering the inherent topological relationships, we propose that macrocyclic complexes may also be obtained when the ligand is nonplanar^43^. In this study, we choose a very simple nonplanar bidentate ligand (**1**) and Pd^II^ ions as our building blocks (Fig. 1). Ligand **1** has two unique features: first it is not conjugated, so that two benzoimidazole rings are reasonably free to rotate and bent, giving rise to the conformational flexibility of its coordination geometry^44^; and second benzoimidazole bears an acidic CH bond that can act as hydrogen-bond donor, especially after its coordination to a metal^45^,^46^. In fact, varieties of anion-binding hosts utilizing the imidazole motif, either of pure organic or metal-organic forms, have been reported^47^. We envisage that anions with different size and shape will dictate the arrangement of ligand **1** during the self-assembly process, thus providing the driving force for the otherwise complicated system towards thermodynamically preferable outcome.

**Figure 2:**
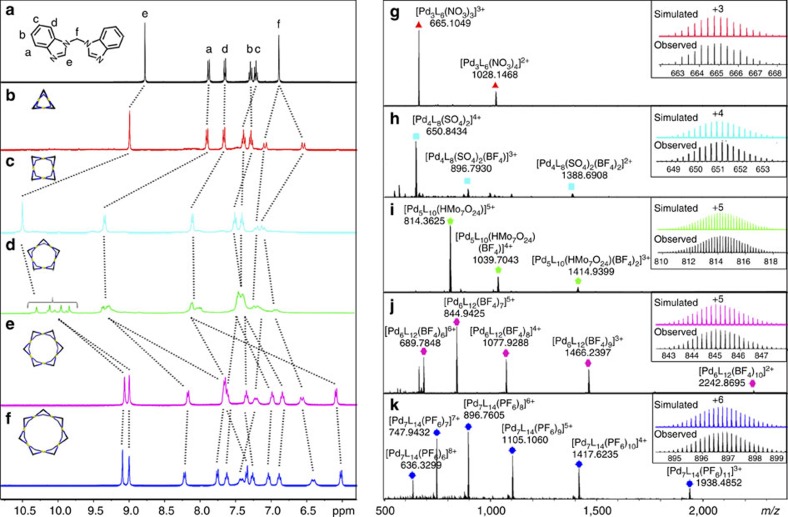
^1^H NMR and ESI-TOF-MS spectra. (**a**–**f**) are ^1^H NMR (400 MHz, [D_6_]DMSO, 298 K) spectra for free ligand **1** and the complexes **2**, **3**, **4**, **5**, **6**, respectively; (**g**–**k**) are ESI-TOF-MS spectra for complexes **2**, **3**, **4**, **5** and **6** with insets showing the representative observed and calculated isotope patterns.

Reaction of ligand **1** (20.07 μmol) with a half equivalent of Pd(NO_3_)_2_ (10.04 μmol) in 1 ml dimethyl sulfoxide ([D_6_]DMSO) at 70 °C for 5 h leads to the quantitative formation of a single compound, as confirmed by ^1^H NMR spectroscopy (Fig. 2a,b). Compared with the free ligand, the peak of H_e_ on benzimidazole of the assembly was shifted downfield from 8.77 to 9.00 p.p.m., suggesting the loss of electron density on the imidazole ring due to coordination or the involvement of Hydrogen bonding. The diastereotopic environment of the methylene protons, which split into a pair of doublets in a 1:1 ratio, indicates a *cis* conformation of the ligands in the product^44^. Diffusion-ordered NMR spectrum (DOSY) (Supplementary Fig. 4) confirmed that all peaks have the same diffusion coefficient, with an estimated diameter of 1.12 nm for the assembly. The composition of product formulated as Pd_3_L_6_(NO_3_)_6_ (**2**) was then clearly provided by high-resolution ESI-TOF-MS. Prominent peaks observed at *m/z*=1028.1468, and 665.1049 correspond to the multiple-charged [**2**–(NO_3_^−^)_*n*_]^*n*+^ (*n*=2, 3) molecular-ion signals, with consecutive loss of the NO_3_^−^ counter-ions. Moreover, the isotopic patterns of each resolved peaks were also in good agreement with the simulated values (Fig. 2g).

To our surprise, a clearly distinct product was obtained when Pd(BF_4_)_2_ was used during the self-assembly, in a similar reaction condition as described above (See Methods section for details). As shown in Fig. 2e, all the proton signals of the ligand on this complex strongly split into two sets in a 1:1 ratio, with one set of signals obviously downfield shifted and the other upfield shifted (except for the CH on imidazole) with respect to those of the free ligand. Such observation is clearly different from the trinuclear compound **2**, where only the protons on the methylene groups are split. This suggests two different chemical environments for the benzoimidazole moities in the final product. On the basis of ^1^H-^1^H COSY experiment, all the signals could be fully assigned (Supplementary Fig. 8). DOSY spectrum (Supplementary Fig. 9) reveals the formation of a new product with an estimated diameter of 2.23 nm, which is dramatically larger than that of **2**. ESI-TOF-MS (Fig. 2j) discloses that this complex is formulated as Pd_6_L_12_·(BF_4_)_12_ (**5**), with prominent peaks observed at *m/z*=689.7848, 844.9425, 1077.9288, 1466.2397 and 2242.8695, corresponding to [**5**–(BF_4_^−^)_*n*_]^*n*+^ (*n*=2–6).

On the basis of this unexpected structural switch from Pd_3_L_6_ to Pd_6_L_12_ by varing the counter ions from NO_3_^−^ to BF_4_^−^, we then postulate that other counter ions with larger size may induce the formation of higher nuclear assembly. Indeed, pure heptanuclear Pd_7_L_14_·(PF_6_)_14_ (**6a**) or Pd_7_L_14_(OTf)_14_ (**6b**) complexes were quantitatively obtained by replacing the metal source with Pd(PF_6_)_2_ or Pd(OTf)_2_, respectively, the composition of which were determined in a similar manner by NMR and ESI-TOF-MS (Fig. 2f,k and Supplementary Figs 13–21). Similar diastereotopic splitting of ^1^H NMR signals into 1:1 ratio was also observed for compound **6**.

Direct synthesis of the missing Pd_*n*_L_2*n*_ macrocycles between *n*=3 and 6 were unsuccessful. Instead, the intermediate-sized Pd_4_L_8_ and Pd_5_L_10_ could be obtained by anion-induced transformation processes. In a typical procedure, tetrabutylammonium hydrogen sulfate (10.27 mg, 30.24 μmol, 4.5 eq.) was added to a 1 ml [D_6_]DMSO solution of compound **5** (6.72 μmol) and the mixture was heat at 70 °C for 3 h. 1D (Fig. 2c) and 2D NMR (Supplementary Figs 25 and 26) indicated the total transformation of **5** into a new product. In the ^1^H NMR, the diagnostic diastereomeric splitting of the ligands signals on **5** gradually disappeared with the origin of another single set of signals (except for the methylene protons). Moreover, H_e_ on the imidazole rings were significantly shifted downfield from 8.75 p.p.m. and 7.86 p.p.m. to 10.46 p.p.m.), indicating the involvement of strong Hydrogen-bonding interactions with the bisulfate ions. DOSY revealed that the new complex was of a diameter of 1.51 nm, a value in between of the compound **2** and **5**. Even with intrinsic mixed counter anions, ESI-TOF-MS spectrum clearly confirmed the formation of the tetranuclear Pd_4_L_8_ compound (**3**), with prominent peaks observed at *m/z*=650.8434, 896.7930, 1388.6908, corrsponding to [Pd_4_L_8_(SO_4_)_2_]^4+^, [Pd_4_L_8_(SO_4_)_2_(BF_4_)_1_]^3+^, [Pd_4_L_8_(SO_4_)_2_(BF_4_)_2_]^2+^, respectively. The loss of protons on bisulfates anions was possibly due to the total 8+ charge on the host or the presence of multiple Hydrogen bonding between the host and anions. Under similar conditions, induced-fit transformation from **6** to **3** was also quantitative (see discussion below).

The conversion from M_6_L_12_ to the M_5_L_10_ structure was beyond anticipation. Our initial thought was to induce the formation of higher-nuclear Pd_*n*_L_2*n*_ complexes (for *n*>7), using bulkier counter-ions. However, treating compound **5** (6.72 μmol) with 0.8 eq. tetrabutylammonium heptamolybdate ((*t*-Bu_4_N)_6_Mo_7_O_24_, 13.48 mg, 5.38 μmol) in 1 ml [D_6_]DMSO solution converted **5** into Pd_5_L_10_ (**4**) (same conditions as above). The formation of this new complex was indicated by ^1^H NMR (Fig. 2d), where all signals become severely broadened with the H_e_ significantly split into a complicated pattern, presumably due to a mismatch of symmetry between the host and the guest anion. However, ^1^H NMR at 100 °C becomes much simplified (Supplementary Fig. 30). In particular, the complicated pattern observed for H_e_ coalesced into one single broadened peak with clear upfield-shifting, indicating the break of H bonds and increase of symmetry at elevated temperatures. DOSY revealed that the new complex was of a diameter of 1.84 nm (Supplementary Fig. 34). ESI-TOF-MS reveals that a new pentanuclear Pd_5_L_10_ compound (**4**) with an encapsulated [HMo_7_O_24_]^5−^ cluster was formed, with prominent peaks observed at *m/z* = 814.3625, 1039.7043 and 1414.9399 corresponding to [Pd_5_L_10_(HMo_7_O_24_)_1_]^5+^, [Pd_5_L_10_(HMo_7_O_24_)_1_(BF_4_)_1_]^4+^ and [Pd_5_L_10_(HMo_7_O_24_)(BF_4_)_2_]^3+^, respectively (Fig. 2i).

The structures of the self-assembled Pd_*n*_L_2*n*_ complexes for *n*=3, 4, 5, 6 were unambiguously determined by single crystal diffraction studies facilitated by high-power X-ray source at the Shanghai Synchrotron Radiation Facility (SSRF). Crystal structures reveal that all complexes shares a similar donut-shaped structure, where *2n* bent bidentate ligands are connected by *n* square-planar coordinated Pd^II^ ions in a cyclic fashion. Ligands can be divided into two layers by an imaginary plane of the metal centres. On the basis of crystal structures, we can clearly see that hydrogen-bonding interactions between the host framework and its entrapped anions played an important role in dictating the size of the macrocycles.

**Figure 3:**
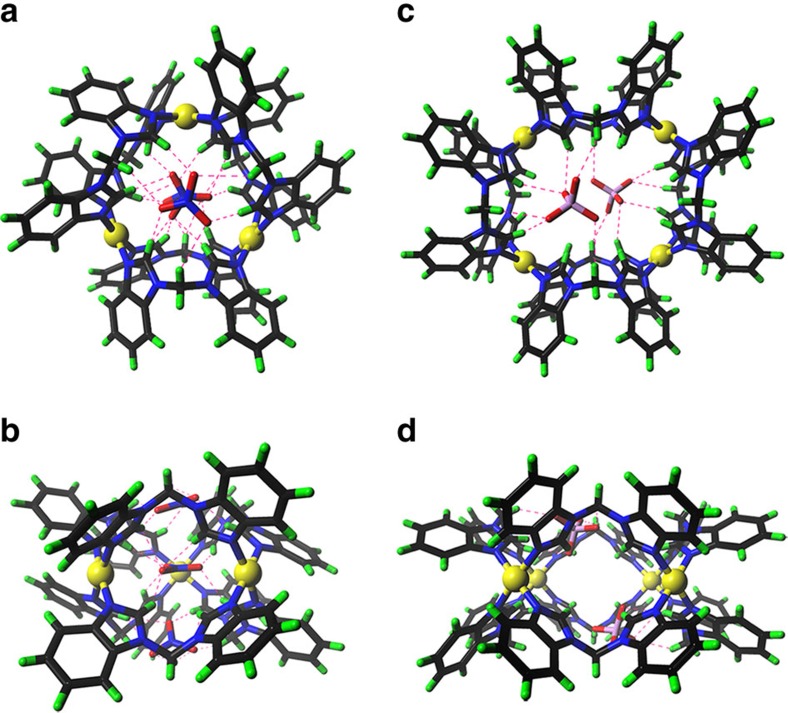
X-ray single crystal structures of compounds 2 and 3. Top (**a**,**c**) and side (**b**,**d**) views of the X-ray crystal structures of compounds **2** and **3**. (Colour scheme: H bondings, red dashed lines; Pd, yellow; C, black; N, blue; S, pink; O, red; H, green. Solvents and anions located outside the macrocycles are omitted for clarity.)

High quality single crystals of compound **2** suitable for crystallographic analysis were obtained by slow vapour diffusion of dichloromethane (DCM) into a DMSO solution of the complex over 3 weeks, which crystallized in a *P_–1_* space group with two macrocycles sitting in the unit cell. This smallest donut-shaped assembly has an out diameter of 1.74 nm and a height of 0.87 nm, which can be regarded as a trimeric structure consisting three Pd_2_L_2_ square building blocks by sharing the Pd^II^ centres (Fig. 3a,b). Interestingly, three nitrate anions were H bonded inside the cavity, which form a three-layered sandwich conformation. One NO_3_^−^ is in a perpendicular orientation to the other two parallel ones, possibly due to electrostatic repulsion. Each nitrate is involved in at least six Hydrogen-bonding interactions with the inward CH from the imidazole and methylene groups. We propose these multi-point H bonds act as glues to tie the ligand strands together. Similarly, diffusion of DCM into a DMSO solution of **3** resulted in the crystalization of this tetranuclear species (Fig. 3c,d). Two SO_4_^2−^ are encapsulated inside the cavity in this case, each of which is sextuple H bonded with three ligands on the same layer.

**Figure 4:**
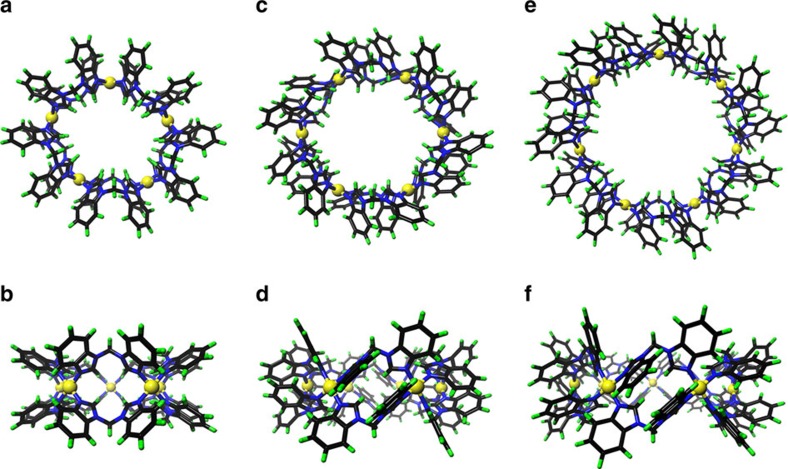
X-ray single crystal structures of compounds 4, 5 and simulated structures of complex 6. Top (**a**,**c**) and side (**b**,**d**) views for the X-ray crystal structures of complexes **4** and **5**; (**e**,**f**) are similar views for the simulated structure of complex **6**. (Same colour schemes were used as for Fig. 3.)

Crystals for the Pd_5_L_10_ complex (**4**) were also obtained by a similar method described above. The pentagonal topology of the framework was clearly confirmed (Fig. 4a,b). Possibly due to the mismatch of symmetry in this host-guest complex, modelling of the encapsulated HMo_7_O_24_^5−^ anion with the known connectivity^48^,^49^ were unsuccessful and its contribution was removed by the SQUEEZE routine^50^. We propose that the HMo_7_O_24_^5−^ guest inside the confined cavity is severely disordered or adopting an unusual geometry. It has to be pointed out that the nature of the encapsulated [HMo_7_O_24_]^5−^ cluster is unclear at this stage and detailed host-guest chemistry of these macrocycles with polyoxometalates deserves further study. After a long trail-and-error, high quality single crystals of **5** were fortunately obtained by a co-crystallization method with 1.0 eq. of NaB(ArF)_4_ (ArF=3,5-bis(trifluoromethyl)phenyl). At this condition, ^1^H or ^19^F NMR (Supplementary Figs 10 and 11) did not show obvious chemical shift for both the host and the [B(ArF)_4_]^−^ anion, indicating that there is no strong interaction between them. Compound **5** crystallized in an orthogonal R_–3c_ space group with a huge unite cell volume of 119,056 Å^3^ (Fig. 4c,d). The dimension of this self-assembled macrocycle spans an out diameter of 2.24 nm and inner diameter of 1.19 nm. Compared to the symmetrical Pd_2_L_2_ squares units existing on the crystal structures of **2**, **3** and **4**, the six Pd_2_L_2_ units on **5** were severely distorted into a rectangular conformation, which provides a good reason for the split signals observed in its ^1^H NMR. Such structural distortion also reflects the flexibility of ligand **1**. Due to the low-diffraction nature of this sample, only the Pd_6_L_12_ connectivity was confirmed and no detailed anion binding mode could be modelled. In crystal packing, two giant channels are present along the *c* axis due to layer-by-layer stacking of the ring-shaped complexes, one of which is defined by the ring cavity with a diameter of 1.19 nm, the other is 1.75 nm encircled by six adjacent molecules, where we hypothesize is filled with the highly disordered bulky [B(ArF)_4_]^−^ anions. Many attempts to crystallize the heptanuclear **6** were unsuccessful, possibly due to its low symmetry and big size. This structure was then modelled by molecular mechanical optimizations and depicted in Fig. 4e,f.

## Discussion

**Figure 5:**
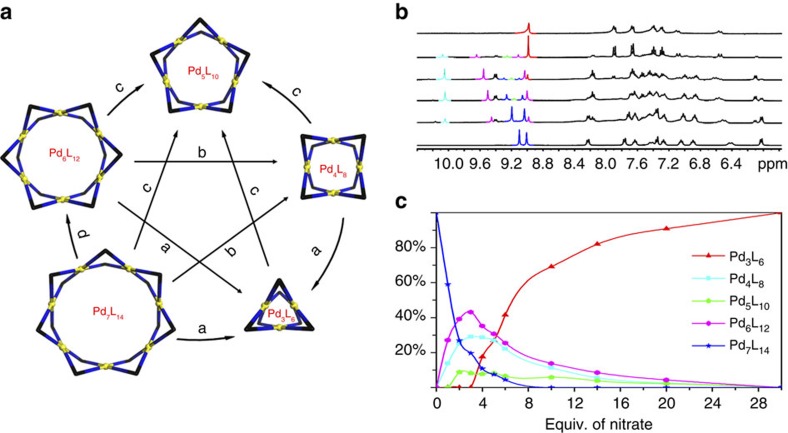
Induce-fit transformations between macrocycles. (**a**) Cross map showing all the transformations between the macrocyclic species (conditions for a: NO_3_^−^ ; b: HSO_4_^−^; c: Mo_7_O_24_^6−^; d: BF_4_^−^); (**b**) ^1^H NMR (400 MHz, [D_6_]DMSO, 298 K) titration spectra showing the gradual transformation from the heptanuclear complex **6a** to the trinuclear complex **2** by sequential addition of KNO_3_ (bottom up: 0, 1, 2, 4, 10, 30 eq.) with H_e_ signals highlighted for Pd_3_L_6_: red, Pd_4_L_8_: sky blue, Pd_5_L_10_: green, Pd_6_L_12_: pink and Pd_7_L_14_: dark blue, respectively; (**c**) Composition distributions for all the Pd_*n*_L_2*n*_ species obtained by the integrals of the H_e_ signals from the titration experiment.

Taking advantage of the anion-adaptivity observed during the self-assembly, we then managed to draw a comprehensive map detailing the induced-fit transformation processes between all species of the family (Fig. 5a and Supplementary Figs 42–58). First of all, we found that addition of 30 eq. of KNO_3_ to the Pd_7_L_14_ complex (PF_6_^−^ salt) or Pd_6_L_12_ complex **5** resulted in the quantitative transformation to Pd_3_L_6_. The (Mo_7_O_24_)@Pd_5_L_10_ was found to be the the most stable host-guest complex for this dynamic combinatorial system, which could be quantitatively obtained by additon of (*t*-Bu_4_N)_6_Mo_7_O_24_ to either compounds **2**, **3**, **5** or **6**. In fact, this host-guest complex is so stable that once formed it will never transform into other macrocycles. Similarly, Pd_3_L_6_ could be quantitatively obtained by the templation effect of the NO_3_^−^ anion starting from Pd_4_L_8_, Pd_6_L_12_ or Pd_7_L_14_. SO_4_^2−^, on the contrary, is a weaker template comparing to NO_3_^−^, possibly due to the fact that Pd_3_L_6_ can encapsulate three NO_3_^−^ while Pd_4_L_8_ can only host two SO_4_^2−^. Meanwhile, transformation from Pd_7_L_14_ to Pd_6_L_12_ was obtained in only 80% yield even if 35 eq. of BF_4_^−^ anion was used, suggesting only a subtle energy difference between them. On the basis of this map, a sequence of templating-effect like Mo_7_O_24_^6−^>NO_3_^−^>SO_4_^2−^>BF_4_^−^>PF_6_^−^=OTf^−^ was obtained for this dynamic combinatorial system.

Finally, a detailed tranformation process from Pd_7_L_14_ (**6a**) to Pd_3_L_6_ was traced by titration experiments following the sequential addition of NO_3_^−^ (Fig. 5b,c). ^1^H NMR and ESI-TOF-MS showed that the transformation took place via a gradual shrinking of the macrocyclic framework. On the basis of a careful assignment of the NMR and ESI-TOF-MS signals (Supplementary Figs 59–70 and Supplementary Tables 5 and 6), it was found that after adding 1 eq. of NO_3_^−^ ([NO_3_^−^]:[PF_6_^−^]=1:14) the Pd_7_L_14_ macrocycle starts to transform into Pd_6_L_12_ and Pd_4_L_8_, whose contents reach maxima when 3 eq. of NO_3_^−^ were added. Meanwhile, formation of Pd_5_L_10_ was also detected starting from the addition of 2 eq. of NO_3_^−^. All of these intermediates start to drop in contents after the most stable Pd_3_L_6_ complex evolved in the system. We hypothesize that NO_3_^−^ anions are acting as hydrogon-bonding anchors which pull together the neighbouring Pd_2_L_2_ square units on the framework, leading to a gradually shrinking of the ring size. The contents of Pd_6_L_12_ and Pd_4_L_8_ are aboundent during the transformation proecess, possibly due to the fact that they have even numbers of Pd_2_L_2_ square units, which can maximize the number of H bonding with the NO_3_^−^ anchors. In contrast, the concentration of the Pd_5_L_10_ intermediate, which has odd number of Pd_2_L_2_ square units, was rather low (Supplementary Fig. 71).

In summary, a dynamic anion-adaptive self-assembly system was constructed consisting of a very simple ligand and Pd^II^ ions. The anion-induced transformation between the Pd_*n*_L_2*n*_ species are reminiscent of the induced-fit guest-binding mechanism observed in nature. Moreover, this new class of donut-shaped assemblies provide unique tunable hydrogen-binding pockets, where we envisage molecular sensing/catalysis is possible to take place.

## Methods

### Materials

Unless otherwise noted, all chemicals and solvents were purchased from commercial corporations and used without further purification. Deuterated solvents were purchased from Admas and J&K scientific.

### NMR measurements

1D and 2D NMR spectra were measured on a Bruker-BioSpin AVANCE Ш HD (400 MHz) spectrometer. ^1^H NMR chemical shifts were determined with respect to residual solvent signals of the deuterated solvents used. DOSY spectra were applied to estimate the dynamic radius for the compounds **2**, **3**, **4**, **5** and **6** according to the Stokes-Einstein equation (1). Where: *D* is diffusion coefficient obtained from DOSY spectrum, *K*_*B*_ is Boltzmann constant, *T* is temperature, viscosity η was tested to be 2.2 mPa s and *r* is the estimated dynamic radius.


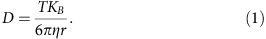


### MS measurements

ESI-Q-TOF mass spectra were recorded on an Impact II UHR-TOF mass spectrometry from Bruker, with ESI-L low concentration tuning mix (from Agilent Technologies) as the internal standard (Accuracy <3 p.p.m.). Data analyses and simulations of ESI-TOF mass spectra were processed on a Bruker Data Analysis software (Version 4.3). Molecular mechanical structure simulations were performed on a Material Studio v6.0 software using the build-in geometry optimization task based on the Universal forcefield.

### Synthesis and characterization

**(Pd**_**3**_**L**_**6**_**)(NO**_**3**_)_**6**_ **(2)** Ligand **1** (ref. 51) (4.98 mg, 20.07 μmol) was treated with Pd(NO_3_)_2_ (10.04 μmol) in [D_6_]DMSO (1 ml) at 70 °C for 5 h. ^1^H NMR confirmed the quantitative formation of complex **2**. ^1^H NMR (400 MHz, [D_6_]DMSO, 298 K) *δ* 8.99 (s, 12H), 7.90 (d, *J*=8.3 Hz, 12H), 7.66 (d, *J*=8.3 Hz, 12H), 7.39 (t, *J*=7.8 Hz, 12H), 7.28 (t, *J*=7.8 Hz, 12H), 7.09 (d, *J*=13.7 Hz, 6H), 6.55 (d, *J*=14.0 Hz, 6H). ^13^C NMR (100 MHz, [D_6_]DMSO, 298 K) *δ* 145.39, 138.88, 130.67, 125.99, 124.90, 117.77, 111.82 and 54.51(Supplementary Figs 1–3). ESI-TOF-MS (NO_3_^−^salt, CH_3_CN): the following picked signals are those at the highest intensities. *m/z* Calcd for [Pd_3_L_6_(NO_3_^−^)_4_]^2+^ 1028.1513, found 1028.1468; Calcd for [Pd_3_L_6_(NO_3_^−^)_3_]^3+^ 665.1049, found 665.1049 (Supplementary Fig. 5).

**(Pd**_**6**_**L**_**12**_**)(BF**_**4**_)_**12**_ **(5)** PdCl_2_ (17.73 mg, 0.10 mmol) was dissolved in [D_6_]DMSO (2 ml) and stirred for 10 h at room temperature with AgBF_4_ (38.94 mg, 0.20 mmol). After removal of AgCl by filtration, the Pd(BF_4_)_2_ stock solution in [D_6_]DMSO was obtained quantitatively. Ligand **1** (20.00 mg, 80.60 μmol) was treated with Pd(BF_4_)_2_ (40.30 μmol) in [D_6_]DMSO (1 ml) at 70 °C for 5 h. After addition of 0.3 ml DCM into the solution, a small amount of precipitation appeared which was then filtrated off and pure compound **5** in [D_6_]DMSO was obtained after removal of DCM. The isolation yield of this compound is about 65%. ^1^H NMR (400 MHz, [D_6_]DMSO, 298 K) *δ* 9.06 (s, 12H), 9.00 (s, 12H), 8.17 (d, *J*=8.2 Hz, 12H), 7.63 (dd, *J*=17.0, 8.0 Hz, 36H), 7.35 (t, *J*=7.8 Hz, 12H), 7.22 (d, *J*=14.6 Hz, 12H), 6.99 (t, *J*=7.8 Hz, 12H), 6.84 (t, *J*=7.5 Hz, 12H), 6.57 (d, *J*=14.8 Hz, 12H), 6.09 (d, *J*=8.2 Hz, 12H). ^19^F NMR (376 MHz, [D_6_]DMSO, 298 K) *δ* -147.31. ^13^C NMR (100 MHz, [D_6_]DMSO, 298 K) *δ* 146.12, 144.82, 138.32, 138.20, 132.34, 128.63, 126.92, 126.53, 126.02, 125.64, 117.88, 117.39, 111.96, 110.55 and 54.36 (Supplementary Figs 6–11). ESI-TOF-MS (BF_4_^−^salt, CH_3_CN): the following picked signals are those at the highest intensities. *m/z* Calcd for [(Pd_6_L_12_)(BF_4_)_9_]^3+^ 1466.2455, found 1466.2397; Calcd for [(Pd_6_L_12_)(BF_4_)_8_]^4+^ 1077.9331, found 1077.9288; Calcd for [(Pd_6_L_12_)(BF_4_)_7_]^5+^ 844.9456, found 844.9425; Calcd for [(Pd_**6**_L_12_)(BF_4_)_6_]^6+^ 689.7873, found 689.7848 (Supplementary Fig. 12).

**(Pd**_**7**_**L**_**14**_**)(PF**_**6**_)_**14**_ **(6a)** PdCl_2_ (17.73 mg, 0.10 mmol) was dissolved in DMSO (2 ml) and stirred for 10 h at room temperature in dark with AgPF_6_ (50.57 mg, 0.20 mmol). After removal of AgCl by filtration, stock solution of Pd(PF_6_)_2_ in [D_6_]DMSO was obtained quantitatively. Then ligand **1** (20 mg, 80.60 μmol) was treated with Pd(PF_6_)_2_ (40.30 μmol) in [D_6_]DMSO (1 ml) at 70 °C for 5 h. ^1^H NMR confirmed the quantitative formation of complex. ^1^H NMR confirmed the quantitative formation of complex **6a**. ^1^H NMR (400 MHz, [D_6_]DMSO, 298 K) *δ* 9.09 (s, 14H), 9.00 (s, 14H), 8.22 (d, *J*=8.4 Hz, 14H), 7.76 (d, *J*=8.2 Hz, 14H), 7.63 (t, *J*=7.5 Hz, 14H), 7.42 (d, *J*=12.7 Hz, 14H), 7.35 (d, *J*=8.4 Hz, 14H), 7.30–7.22 (m, 14H), 7.04 (t, *J*=7.6 Hz, 14H), 6.89 (t, *J*=7.6 Hz, 14H), 6.41 (d, *J*=13.7 Hz, 14H), 6.02 (d, *J*=8.2 Hz, 14H). ^13^C NMR (100 MHz, [D_6_]DMSO, 298 K) *δ* 145.32, 144.72, 138.06, 137.49, 132.14, 128.38, 126.81, 125.89, 117.40, 116.90, 111.79, 110.31 and 54.16 (Supplementary Figs 13–15). ESI-TOF-MS (PF_6_^−^salt, CH_3_CN): the following picked signals are those at the highest intensities. *m/z* Calcd for [(Pd_7_L_14_)(PF_6_)_11_]^3+^ 1938.4758, found 1938.4852; Calcd for [(Pd_7_L_14_)(PF_6_)_10_]^4+^ 1417.6157, found 1417.6235; Calcd for [(Pd_7_L_14_)(PF_6_)_9_]^5+^ 1105.0996, found 1105.1060; Calcd for [(Pd_7_L_14_)(PF_6_)_8_]^6+^ 896.7555, found 896.7605; Calcd for [(Pd_7_L_14_)(PF_6_)_7_]^7+^ 747.9384, found 747.9432; Calcd for [(Pd_7_L_14_)(PF_6_)_6_]^8+^ 636.3255, found 636.3299 (Supplementary Fig. 20).

**(Pd**_**7**_**L**_**14**_**)(CF**_**3**_**SO**_**3**_)_**14**_ **(6b)** PdCl_2_ (17.73 mg, 0.10 mmol) was dissolved in [D_6_]DMSO (2 ml) and stirred for 10 h at room temperature in dark with AgCF_3_SO_3_ (51.39 mg, 0.20 mmol). After removal of AgCl by filtration, the stock solution of Pd(CF_3_SO_3_)_2_ in [D_6_]DMSO was obtained quantitatively. Then ligand **1** (10 mg, 40.29 μmol) was treated with Pd(CF_3_SO_3_)_2_ (20.15 μmol) in [D_6_]DMSO (1 ml) at 70 °C for 5 h. ^1^H NMR confirmed the quantitative formation of complex **6b**. ^1^H NMR (400 MHz, [D_6_]DMSO, 298 K) *δ* 9.19 (d, *J*=8.6 Hz, 28H), 8.21 (d, *J*=8.2 Hz, 14H), 7.72 (d, *J*=7.9 Hz, 14H), 7.65–7.55 (m, 14H), 7.40–7.28 (m, 28H), 7.27–7.17 (m, 14H), 7.07–6.96 (m, 14H), 6.91–6.80 (m, 14H), 6.53 (d, *J*=14.5 Hz, 14H), 6.00 (d, *J*=7.8 Hz, 14H). ^13^C NMR (100 MHz, [D_6_]DMSO, 298 K) *δ* 145.32, 144.72, 138.06, 137.49, 132.14, 128.38, 126.81, 125.89, 117.40, 116.90, 111.79, 110.31 and 54.16 (Supplementary Figs 16–18). ESI-TOF-MS (CF_3_SO_3_^−^ salt, CH_3_CN): the following picked signals are those at the highest intensities. *m/z* Calcd for [(Pd_7_L_14_)(CF_3_SO_3_)_11_]^3+^ 1953.4308, found 1953.4414; Calcd for [(Pd_7_L_14_)(CF_3_SO_3_)_10_]^4+^ 1427.8350, found 1427.8426; Calcd for [(Pd_7_L_14_)(CF_3_SO_3_)_9_]^5+^ 1112.4775, found 1112.4827; Calcd for [(Pd_7_L_14_)(CF_3_SO_3_)_8_]^6+^ 902.2392, found 902.2435; Calcd for [(Pd_7_L_14_)(CF_3_SO_3_)_7_]^7+^ 751.9261, found 751.9297; Calcd for [(Pd_7_L_14_)(CF_3_SO_3_)_6_]^8+^ 751.9261, found 751.9297; Calcd for [(Pd_7_L_14_)(CF_3_SO_3_)_5_]^9+^ 639.3163, found 639.3199 (Supplementary Fig. 21).

**(Pd**_**4**_**L**_**8**_**)(SO**_**4**_)_**2**_**(BF**_**4**_)_**4**_ **(3)** the solution of **5** (6.72 μmol) was treated with tetrabutylammonium hydrogen sulfate (10.27 mg, 30.24 μmol, 4.50 eq.) at 70 °C for 3 h. ^1^H NMR confirmed the quantitative formation of complex **3**. ^1^H NMR (400 MHz, [D_6_]DMSO, 298 K) *δ* 10.46 (s, 16H), 9.30 (d, *J*=8.4 Hz, 16H), 8.06 (d, *J*=8.5 Hz, 16H), 7.46 (t, *J*=7.7 Hz, 16H), 7.36 (t, *J*=7.6 Hz, 16H), 7.16 (d, *J*=15.4 Hz, 8H), 7.07 (d, *J*=14.8 Hz, 8H). ^13^C NMR (100 MHz, DMSO, 298 K) *δ* 145.61, 138.53, 132.16, 125.77, 124.92, 118.68, 112.05 and 57.76 (Supplementary Figs 22–25). ESI-TOF-MS (SO_4_^2−^/BF_4_^−^salt, CH_3_CN): the following picked signals are those at the highest intensities. *m/z* Calcd for [(Pd_4_L_8_)(SO_4_)_2_(BF_4_)_2_]^2+^ 1388.6898, found 1388.6908; Calcd for [(Pd_4_L_8_)(SO_4_)_2_(BF_4_)]^3+^ 896.7918, found 896.7930; Calcd for [(Pd_4_L_8_)(SO_4_)_2_]^4+^ 650.8428, found 650.8434 (Supplementary Figs 27 and 28).

**(Pd**_**5**_**L**_**10**_**)(HMo**_**7**_**O**_**24**_**)(BF**_**4**_)_**5**_ **(4)** the solution of **5** (6.72 μmol) was treated with tetrabutylammonium heptamolybdate (13.48 mg, 5.38 μmol, 0.80 eq.) at 70 °C for 3 h. ^1^H NMR confirmed the quantitative formation of complex **4**. ^1^H NMR (400 MHz, [D_6_]DMSO, 298 K) *δ* 10.01 (ddd, *J*=65.3, 61.9, 39.5 Hz, 20H), 9.28 (t, *J*=28.2 Hz, 20H), 8.21–7.91 (m, 20H), 7.42 (d, *J*=8.0 Hz, 20H), 7.37 (s, 20H), 7.18 (s, 10H), 6.91 (s, 10H). ^13^C NMR (100 MHz, DMSO,298 K) *δ* 144.61, 138.26, 130.82, 125.10, 124.49, 119.10 and 111.60 (Supplementary Figs 29–33). ESI-TOF-MS (HMo_7_O_24_^5−^, BF_4_^−^salt, CH_3_CN): the following picked signals are those at the highest intensities. *m/z* Calcd for [(Pd_5_L_10_)(HMo_7_O_24_)]^5+^ 814.3625, found 814.3622; Calcd for [(Pd_5_L_10_)(HMo_7_O_24_)(BF_4_)]^4+^ 1039.7043, found 1039.7051; Calcd for [(Pd_5_L_10_)(HMo_7_O_24_)(BF_4_)_2_]^3+^ 1414.9399, found 1414.9400 (Supplementary Figs 35 and 36).

Transformation of **6a** into **5** by **BF**_**4**_^**−**^: 35 eq. of N(C_4_H_9_)_4_BF_4_ was added into the prepared solution of compound **6a,** followed by stirring for 3 h at 70 °C. ^1^H NMR and ESI-TOF-MS spectra revealed that compound **6a** changed into **5** (with mixed counter anions of PF_6_^−^ and BF_4_^−^) in 80% yield (Supplementary Figs 42–44 and Supplementary Table 5).

Transformation of **6a** into **4** by **Mo**_**7**_**O**_**24**_^**6−**^: 0.8 eq. of [N(C_4_H_9_)_4_]_6_Mo_7_O_24_ was added into the prepared solution of compound **6a,** followed by stirring for 3 h at 70 °C. ^1^H NMR and ESI-TOF-MS spectra revealed that compound **6a** quantitatively changed into **4** (with mixed counter anions of PF_6_^−^ and HMo_7_O_24_^5−^) (Supplementary Figs 36 and 45).

Transformation of **6a** into **3** by **HSO**_**4**_^**−**^: 5.25 eq. of N(C_4_H_9_)_4_HSO_4_ was added into the prepared solution of compound **6a,** followed by stirring for 3 h at 70 °C. The ^1^H NMR and ESI-TOF-MS spectra revealed that the compound **6a** quantitatively changed into **3** (with mixed counter anions of PF_6_^−^ and SO_4_^−^) completely (Supplementary Figs 28 and 46).

Transformation of **6a** into **2** by **NO**_**3**_^**−**^: 30 eq. of KNO_3_ was added into the prepared solution of compound **6a,** followed by stirring for 3 h at 70 °C. The ^1^H NMR and ESI-TOF-MS spectra revealed the compound **6a** quantitatively changed into **2** (with mixed counter anions of PF_6_^−^ and NO_3_^−^) completely (Supplementary Figs 47 and 48).

Transformation of **5** into **4** by **Mo**_**7**_**O**_**24**_^**6−**^: 0.8 eq. of [N(C_4_H_9_)_4_]_6_Mo_7_O_24_ was added into the prepared solution of compound **5,** followed by stirring for 3 h at 70 °C. The ^1^H NMR and ESI-TOF-MS spectra revealed that compound **5** quantitatively changed into **4** (with mixed counter anions of BF_4_^−^ and HMo_7_O_24_^5−^) (Supplementary Figs 49–35).

Transformation of **5** into **3** by **HSO**_**4**_^**−**^: 4.5 eq. of N(C_4_H_9_)_4_HSO_4_ was added into the prepared solution of compound **5,** followed by stirring for 3 h at 70 °C. The ^1^H NMR and ESI-TOF-MS spectra revealed the compound **5** quantitatively changed into **3** (with mixed counter anions of BF_4_^−^ and SO_4_^−^) (Supplementary Figs 27 and 50).

Transformation of **5** into **2** by **NO**_**3**_^**−**^: 12 eq. of KNO_3_ was added into the prepared solution of compound **5,** followed by stirring for 3 h at 70 °C. The ^1^H NMR and ESI-TOF-MS spectra revealed the compound **5** quantitatively changed into **2** (with mixed counter anions of BF_4_^−^ and NO_3_^−^) (Supplementary Figs 51 and 52).

Transformation of **3** into **4** by **Mo**_**7**_**O**_**24**_^**6−**^: 2.4 eq. of [N(C_4_H_9_)_4_]_6_Mo_7_O_24_ was added into the prepared solution of compound **3,** followed by stirring for 3 h at 70 °C. The ^1^H NMR and ESI-TOF-MS spectra revealed the compound **3** quantitatively changed into **4** (with mixed counter anions of HSO_4_^−^, BF_4_^−^ and HMo_7_O_24_^5−^) (Supplementary Figs 53 and 54).

Transformation of **3** into **2** by **NO**_**3**_^**−**^: 13.3 eq. of KNO_3_ was added into the prepared solution of compound **3,** followed by stirring for 3 h at 70 °C. The ^1^H NMR and ESI-TOF-MS spectra revealed the compound **3** quantitatively changed into **2** (with mixed counter anions of HSO_4_^−^, BF_4_^−^ and NO_3_^−^) (Supplementary Figs 56 and 55).

Transformation of **2** into **4** by **Mo**_**7**_**O**_**24**_^**6−**^: 2 eq. of [N(C_4_H_9_)_4_]_6_Mo_7_O_24_ was added into the prepared solution of compound **2,** followed by stirring for 3 h at 70 °C. The ^1^H NMR and ESI-TOF-MS spectra revealed that compound **2** quantitatively changed into **4** (with mixed counter anions of NO_3_^−^ and HMo_7_O_24_^5−^) (Supplementary Figs 57 and 58).

### Single crystal X-ray diffractions

The X-ray diffraction studies for complexes **2**, **3**, **4**, **5** were carried out at the BL17B macromolecular crystallography beamline in SSRF. The collected diffraction data were processed with the HKL2000 software program^52^. All the structures were solved by direct methods and refined by full-matrix least-squares on *F*^2^ with anisotropic displacement using the SHELX software package^53^.

The crystals of these kinds of giant supramolecular assemblies often diffract very weekly in nature. Some of the final *R* factors were converged to very high values, because the crystal was diffracting very weakly due to a large amount of disordered/amorphous solvents and anions that could not be fully located. The electron residuals in such cases were removed by the SQUEEZE routine^54^. We tried our best to finish the refinement but still some A-alerts are found by the (IUCr) checkCIF routine, all of which are due to the poor diffraction nature of the crystals or the disorder of the solvents and anions. Details on crystal data collection and refinement were summarized in Supplementary Tables 1–4.

Crystal data for **2**: space group *P-1*, *a*=18.088(4) Å, *b*=18.299(4) Å, *c*=20.871(4) Å, *V*=6,099(3) Å^3^, *Z*=2, *T*=80 K. Anisotropic least-squares refinement for the framework atoms and isotropic refinement for the other atoms on 30,186 independent merged reflections (*R*_int_=0.0292) converged at residual *wR*_2_=0.3805 for all data; residual *R*_1_=0.1126 for 59494 observed data [*I*>2*σ*(*I*)], and goodness of fit (GOF)=1.048.

Crystal data for **3**: space group *P21c*, *a*=18.178(4) Å, *b*=32.042(6) Å, *c*=18.582(4) Å, *V*=10,616(4) Å^3^, *Z*=2, *T*=293 K. Anisotropic least-squares refinement for the framework atoms and isotropic refinement for the other atoms on 26,904 independent merged reflections (*R*_int_=0.0809) converged at residual *wR*_2_=0.2780 for all data; residual *R*_1_=0.0765 for 93,650 observed data [*I*>2*σ*(*I*)], and goodness of fit (GOF)=1.042.

Crystal data for **4**: space group *Pmmn*, *a=*20.3994(4) Å, *b*=21.2991(4) Å, *c*=29.5319(6) Å, *V*=12,831.3(4) Å^3^, *Z*=2, *T*=293 K. Anisotropic least-squares refinement for the framework atoms and isotropic refinement for the other atoms on 13,683 independent merged reflections (*R*_int_=0.0591) converged at residual *wR*_2_=0.5449 for all data; residual *R*_1_=0.1540 for 18,8179 observed data [*I*>2*σ*(*I*)], and goodness of fit (GOF)=2.991.

Crystal data for **5**: space group *R-3c*, *a*=71.246(4) Å, b=71.246(4) Å, c=27.0831(16) Å, *V=*119056(15) Å^3^, *Z*=18, *T*=293 K. Anisotropic least-squares refinement for the framework atoms and isotropic refinement for the other atoms on 10,282 independent merged reflections (*R*_int_=0.145) converged at residual *wR*_2_=0.4507 for all data; residual *R*_1_=0.1369 for 96,189 observed data [*I*>2*σ*(*I*)], and goodness of fit (GOF)=2.044.

### Data availability

The X-ray crystallographic coordinates for structures reported in this article have been deposited at the Cambridge Crystallographic Data Centre (CCDC), under deposition nos CCDC 1529252-1529255. These data can be obtained free of charge (http://www.ccdc.cam.ac.uk/data_request/cif). All other data are either provided in the Article and its Supplementary Information or are available on request.

## Additional information

**How to cite this article:** Zhang, T. *et al*. Adaptive self-assembly and induced-fit transformations of anion-binding metal-organic macrocycles. *Nat. Commun.* **8**, 15898 doi: 10.1038/ncomms15898 (2017).

**Publisher’s note:** Springer Nature remains neutral with regard to jurisdictional claims in published maps and institutional affiliations.

## Supplementary Material

Supplementary Information
