# Supplementary material for: Adaptive self-assembly and induced-fit transformations of anion-binding metal-organic macrocycles
Source: Nat Commun. 2017 Jun 16;8:15898. doi: 10.1038/ncomms15898 (PMC5481752; doi:10.1038/ncomms15898)
Supplement: Supplementary Information [file ncomms15898-s1.pdf]

Type of file: PDF
Size of file: 0 KB
Title of file for HTML: Supplementary Information
Description: Supplementary Figures and Supplementary Tables

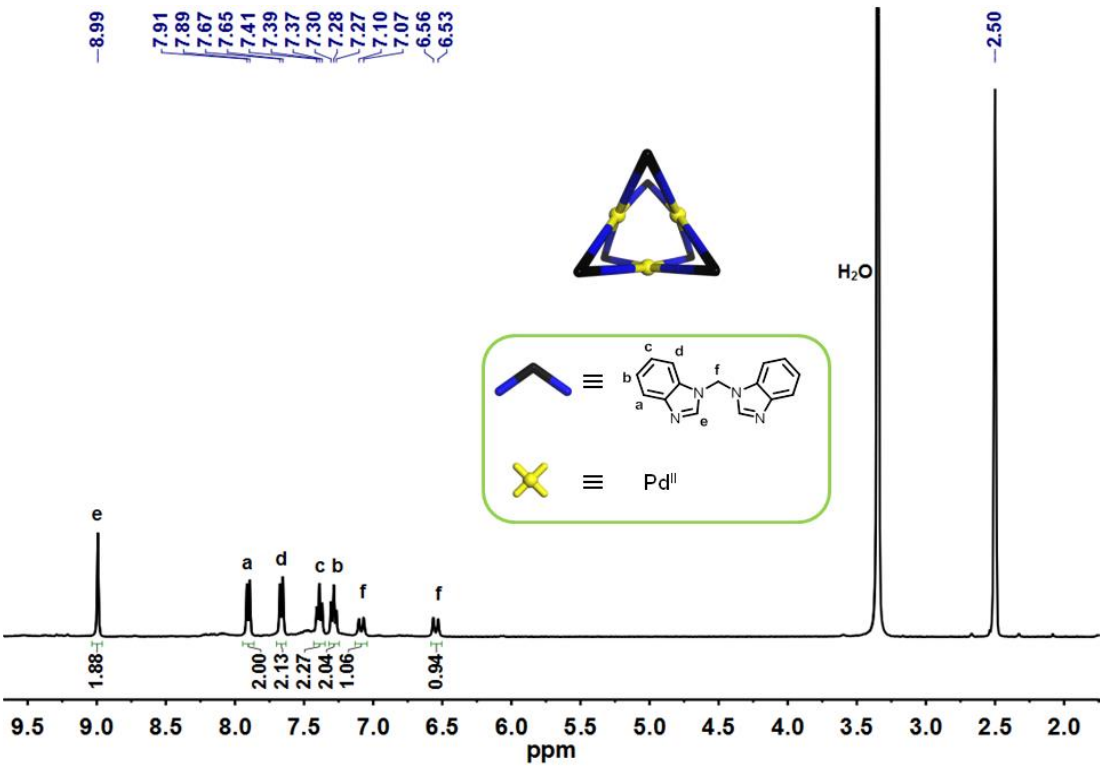

Supplementary Figure 1[1]H NMR spectrum of $(Pd_3L_6)(NO_3)_6$ (400 MHz, $[D_6]$DMSO, 298K).

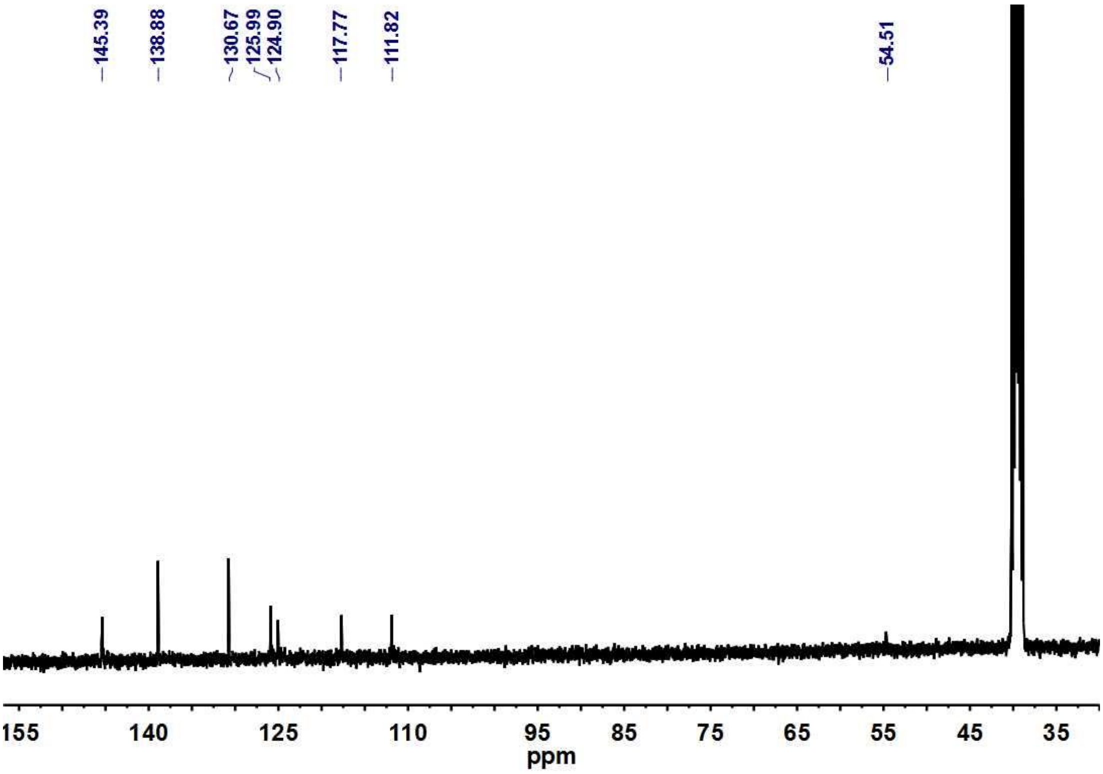

Supplementary Figure 2 ^{13}C NMR spectrum of $(Pd_3L_6)(NO_3)_6$ (100 MHz, $[D_6]$DMSO, 298K).

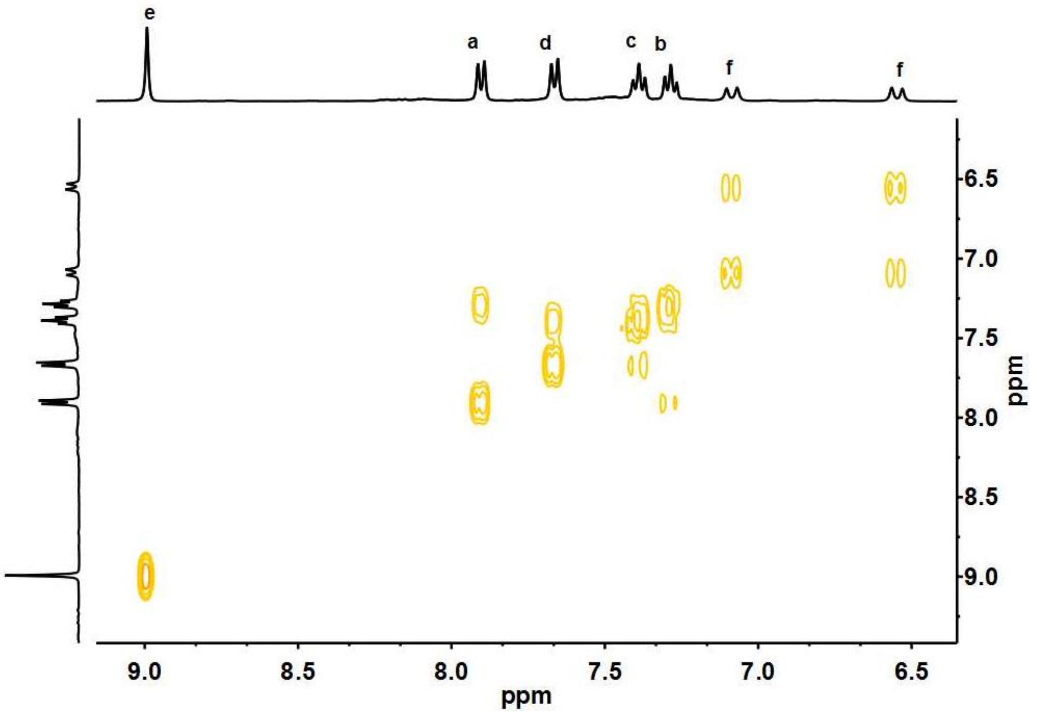

Supplementary Figure 3 ^{1}H-^{1}H COSY NMR spectrum of $(Pd_3L_6)(NO_3)_6$ (400 MHz, [D$_6$]DMSO, 298K).

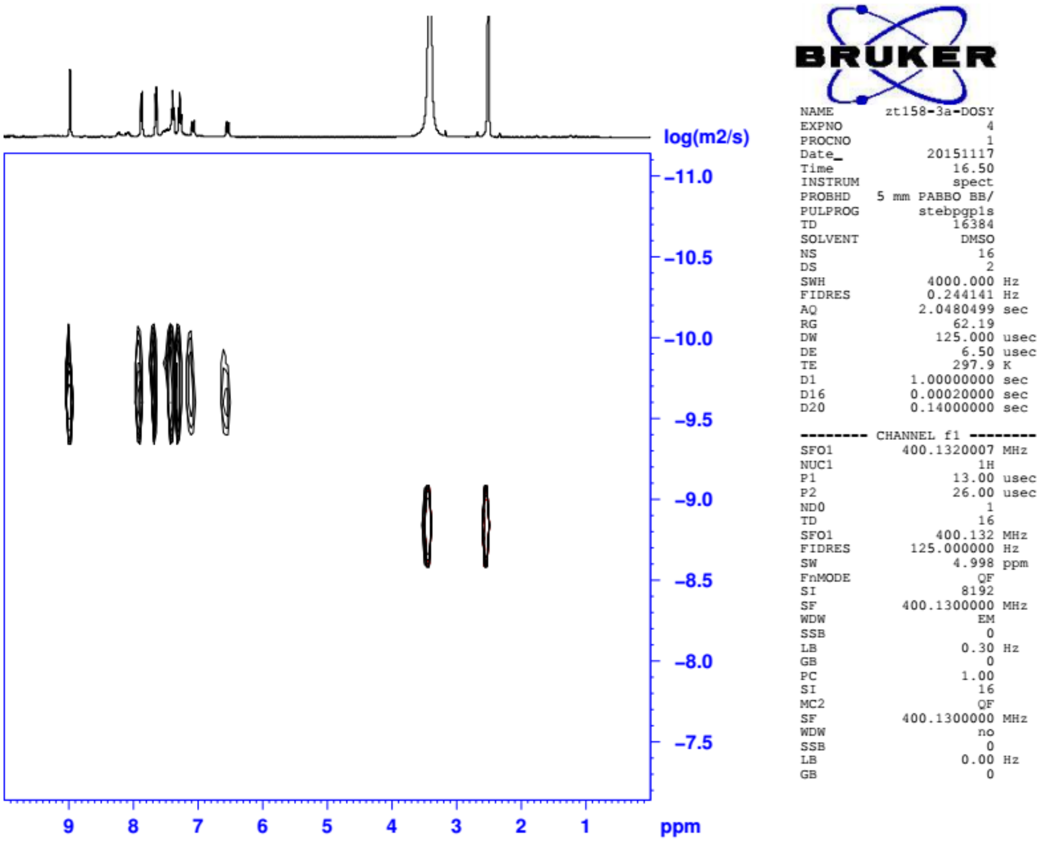

Diffusion Constant = 1.778E-10 m^2/S d=1.12 nm

Supplementary Figure 4 ^{1}H DOSY spectrum of the $(Pd_3L_6)(NO_3)_6$ (400 MHz, [D$_6$]DMSO, 298K).

Display Report

Analysis Info		Acquisition D	2016/4/30 ĐÇÆÚÁù ÏÂÎç 13:29:49
Analysis Name	C:\Users\Administrator\Desktop\project1-pictrue\m3\MS\zt242-6_2-a,8_01_1240.d		
Method	lc-ms-acet-pos-high500-4000.m	Operator	BDAL@DE
Sample Name	zt242-6	Instrumen	impact II 1825265.10133
Comment			

Acquisition Paramet

Source Type	ESI	Ion Polarity	Positive	Set Nebulizer	0.3 Bar
Focus	Active	Set Capillary	4500 V	Set Dry Heater	200 °C
Scan Begin	500 m/z	Set End Plate Offset	-500 V	Set Dry Gas	4.0 l/min
Scan End	4000 m/z	Set Charging Voltage	2000 V	Set Divert Valve	Source
		Set Corona	0 nA	Set APCI Heater	0 °C

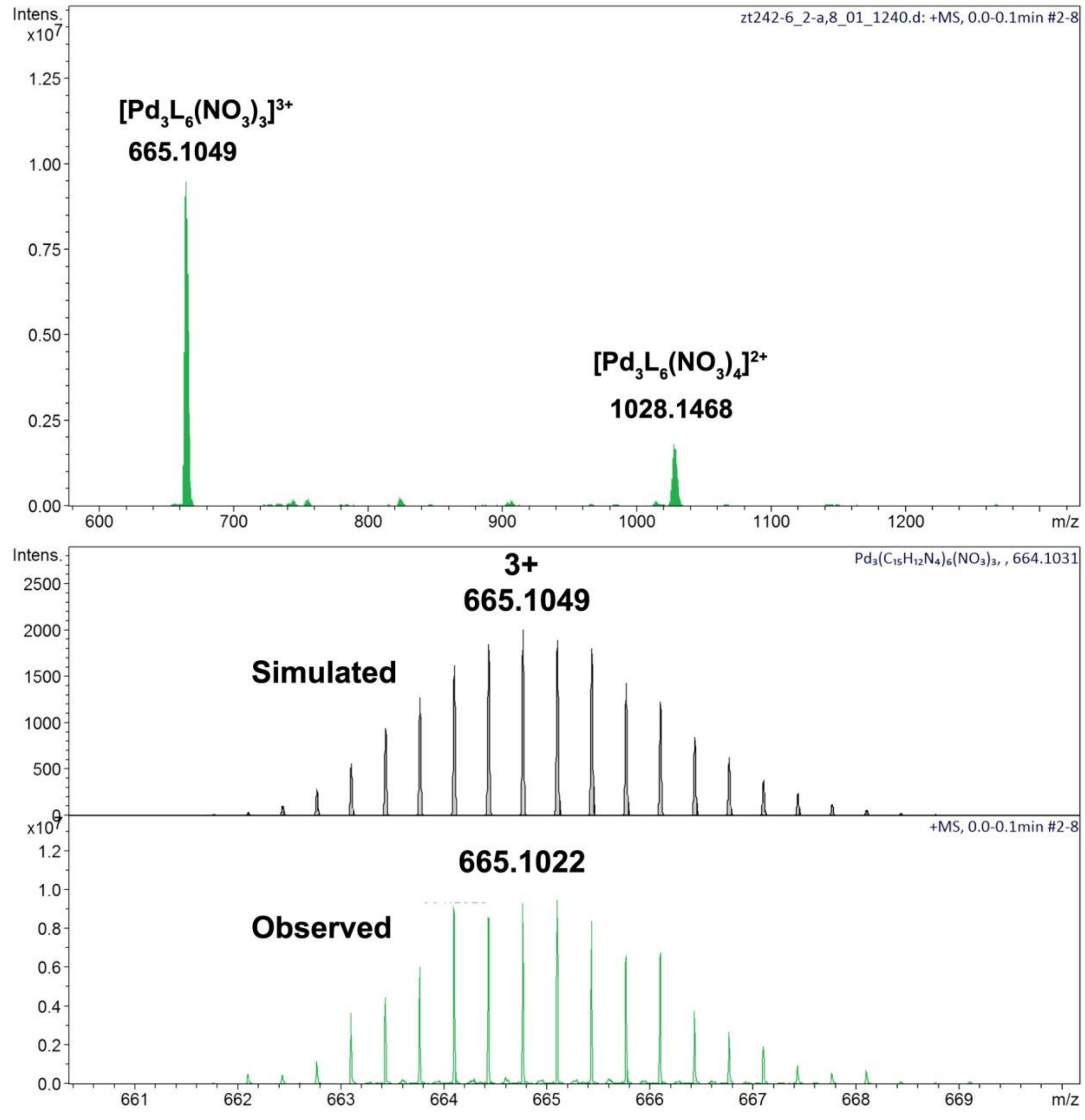

zt242-6_2-a,8_01_1240.d

Bruker Compass DataAnalysis 4.3 printe 2016/11/24 ĐÇÆÚËÄ ÏÂÎç 13:12:04 Page 1 of 1

Supplementary Figure 5 ESI-Q-TOF mass spectrum of $Pd_3\mathbf{L}_6$ (NO_3^- salt) and observed and calculated isotope patterns of 3+.

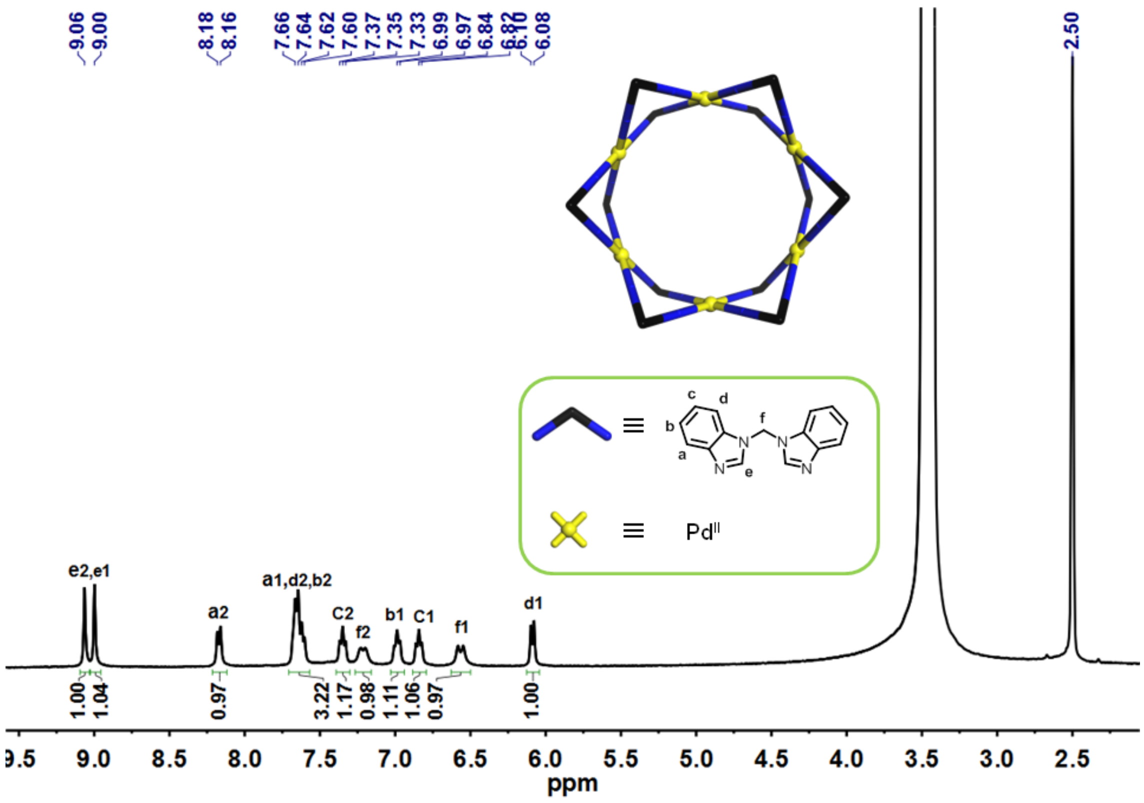

Supplementary Figure 6 ^{1}H NMR spectrum of the $Pd_6L_{12}(BF_4)_{12}$ (400 MHz, $[D_6]$DMSO, 298K).

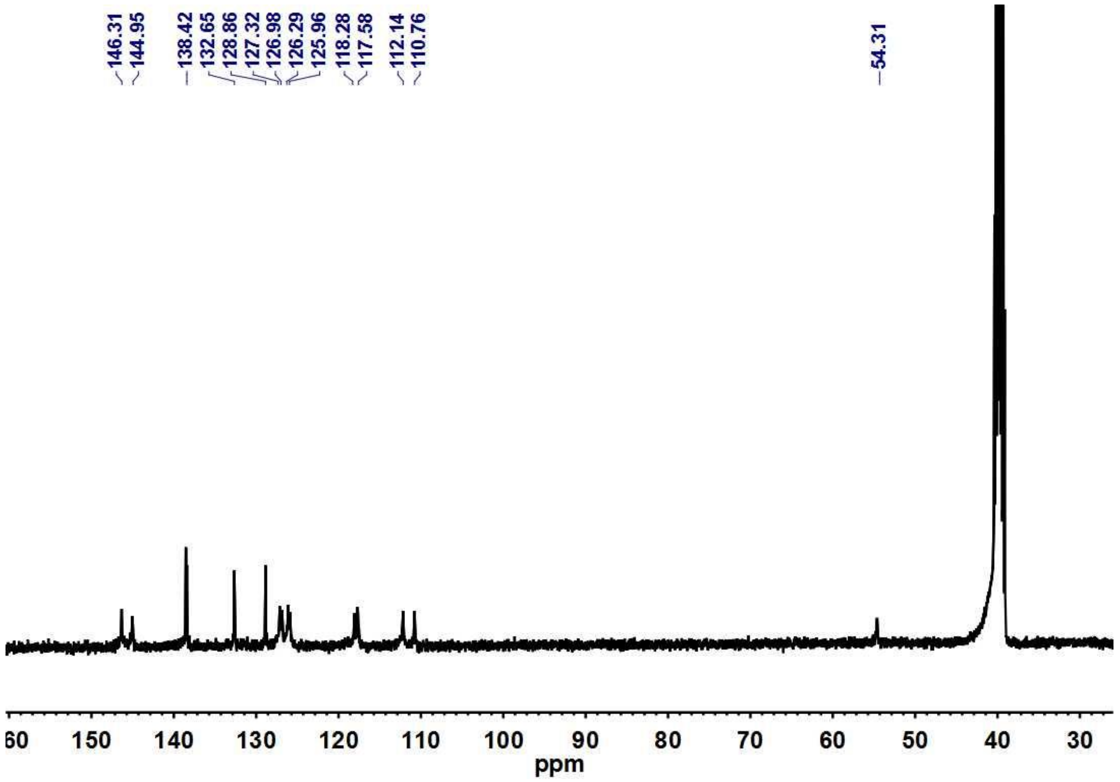

Supplementary Figure 7 ^{13}C NMR spectrum of the $Pd_6\mathbf{L}_{12}(BF_4)_{12}$ (100 MHz, $[D_6]$DMSO,298K).

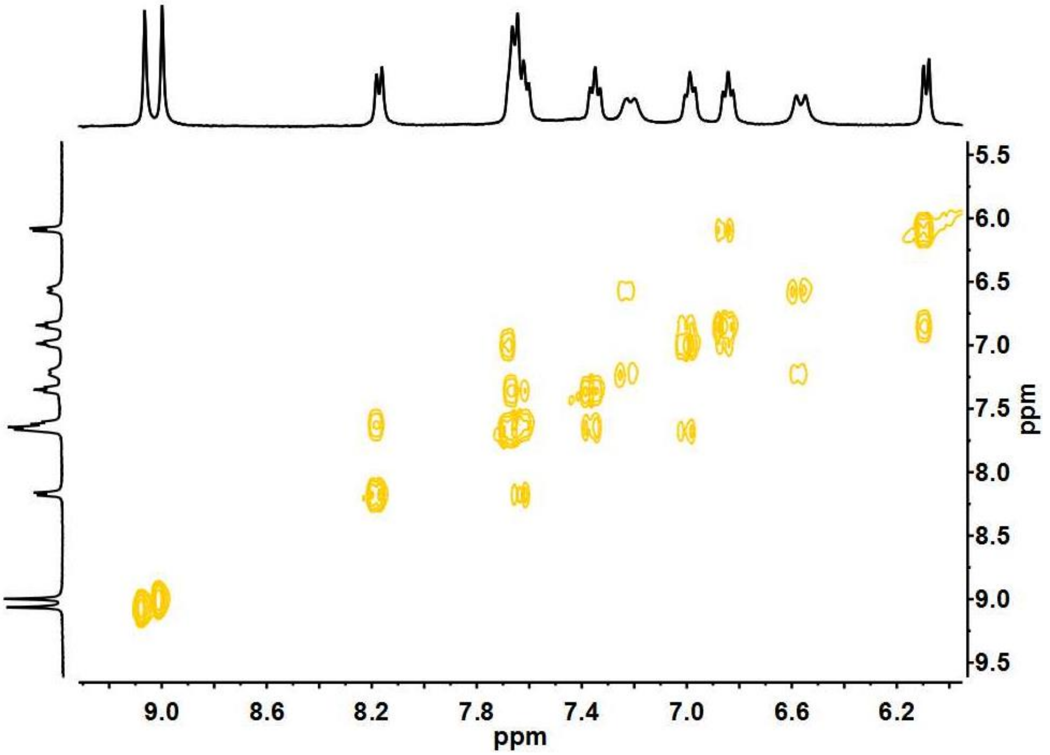

Supplementary Figure 8 ^{1}H-^{1}H COSY NMR spectrum of the $Pd_6L_{12}(BF_4)_{12}$ (400 MHz, [D_6]DMSO, 298K).

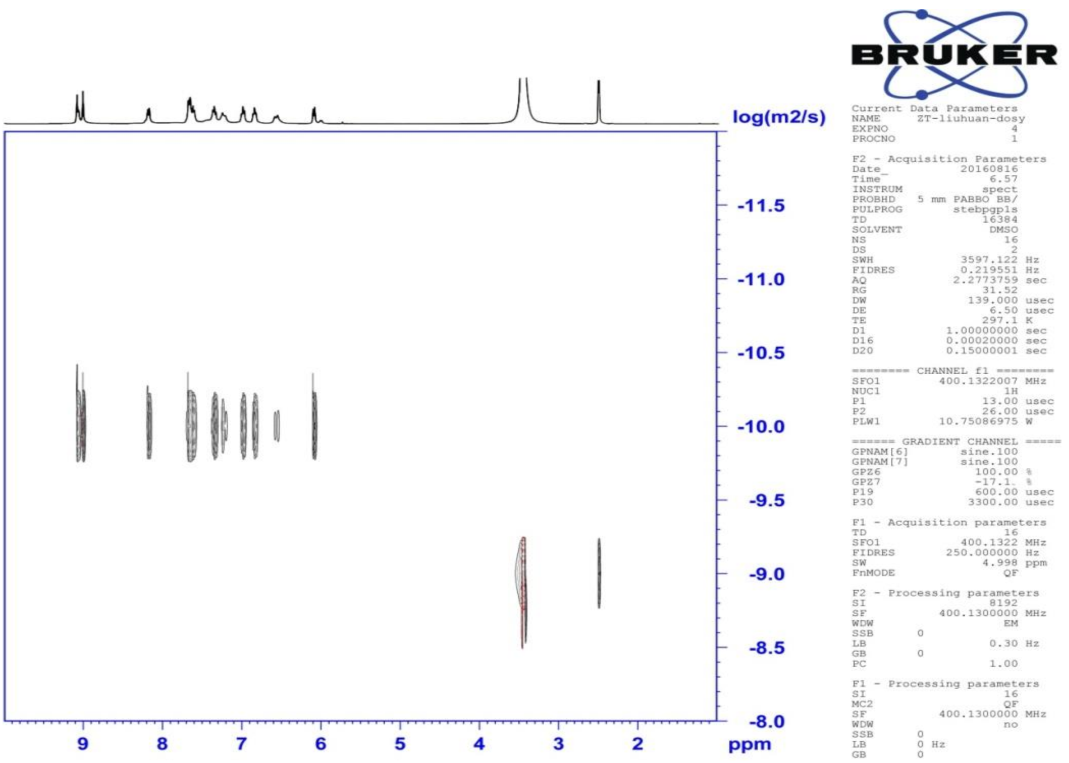

Diffusion Constant = 8.902E-11 m^2/S d=2.23 nm

Supplementary Figure 9 ^{1}H DOSY NMR spectrum of the $(Pd_6L_{12})(BF_4)_{12}$ (400 MHz, [D_6]DMSO, 298K).

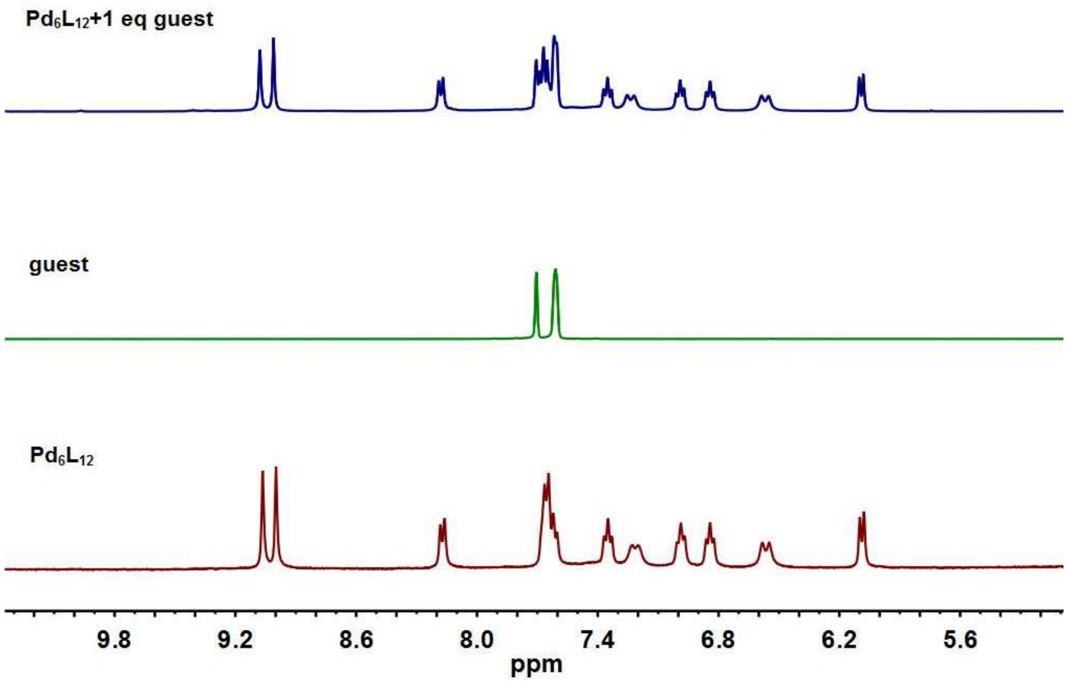

Supplementary Figure 10 ^{1}H NMR spectrum of the $(Pd_6L_{12})(BF_4)_{12}$ (Host), Sodium tetrakis [3,5-bis(trifluoromethyl)phenyl]borate (Guest), and the 1:1 mixture of Host:Guest (from bottom to up) (400 MHz, [D$_6$]DMSO, 298K).

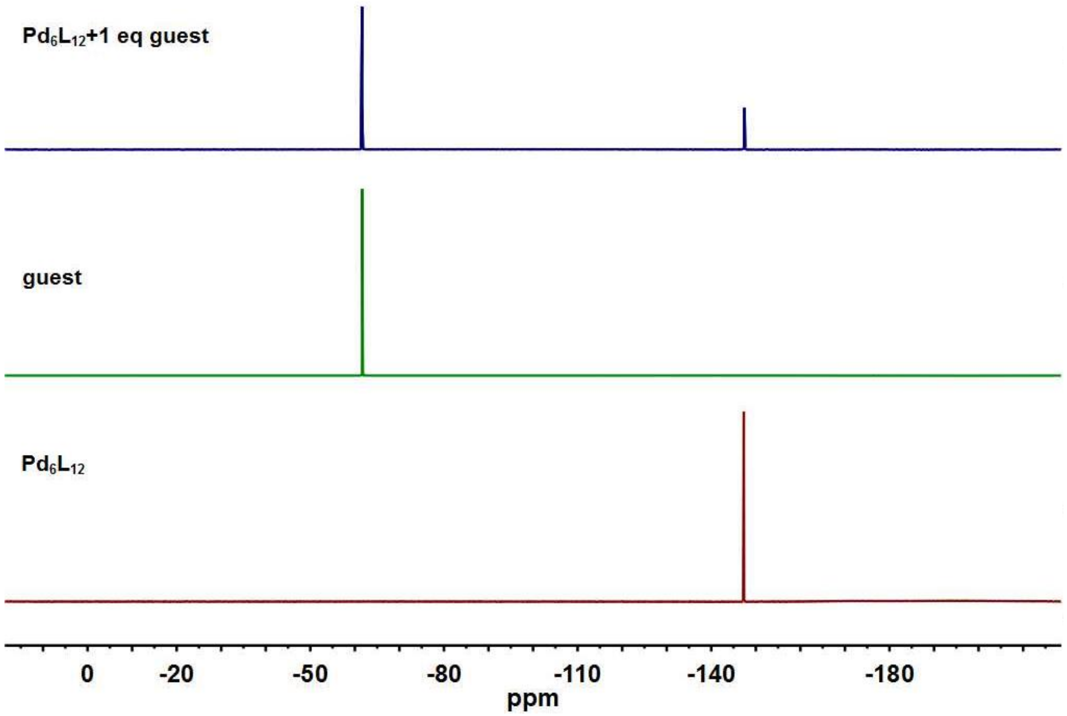

Supplementary Figure 11 ^{19}F NMR spectrum of the $(Pd_6L_{12})(BF_4)_{12}$ (Host), Sodium tetrakis[3,5-bis(trifluoromethyl)phenyl]borate (Guest), and the 1:1 mixture of Host:Guest (from bottom to up) (376 MHz, [D$_6$]DMSO, 298K).

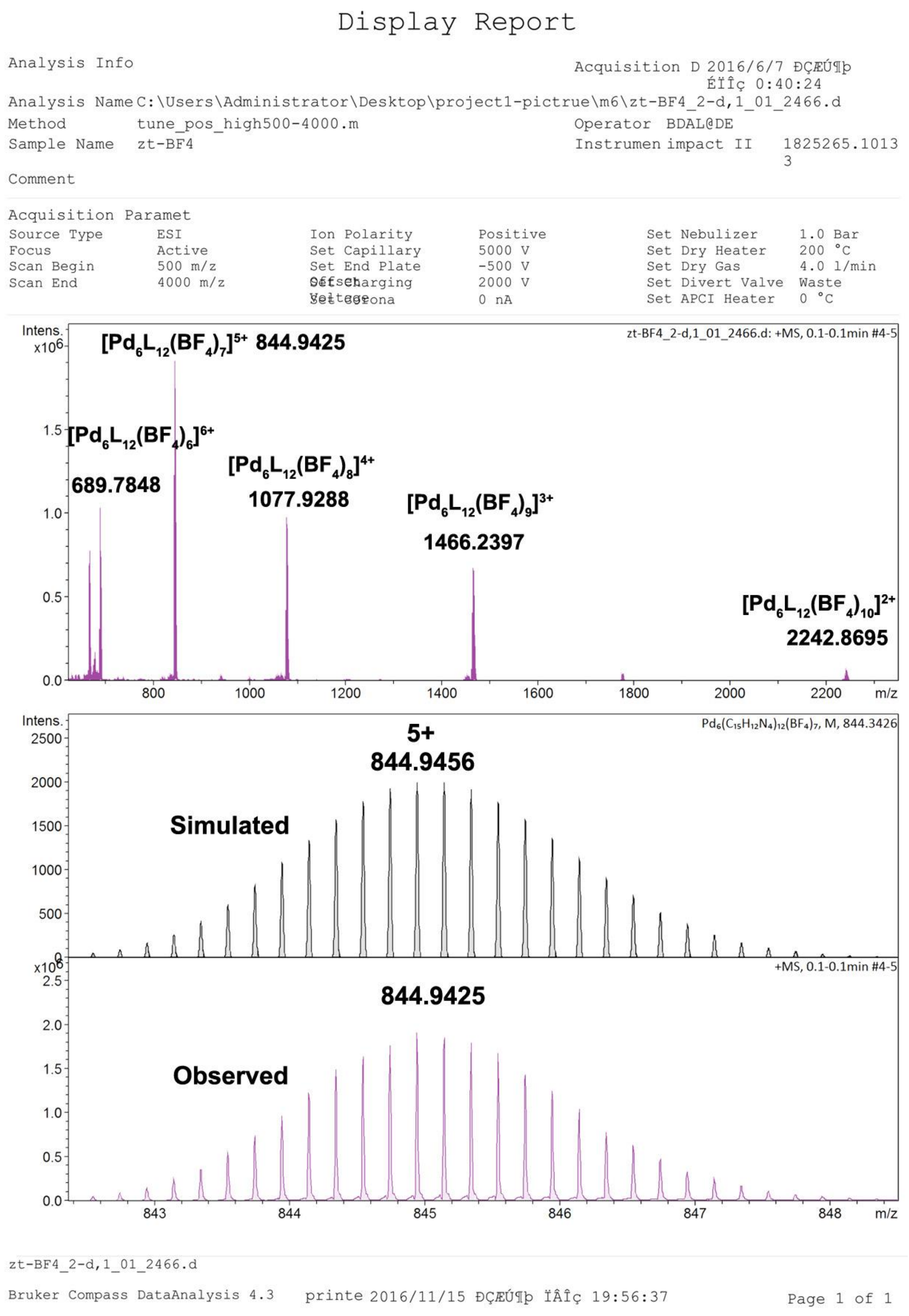

Supplementary Figure 12 ESI-Q-TOF mass spectrum of Pd_6L_{12} (BF_4^- salt) and observed and calculated isotope patterns of 5+

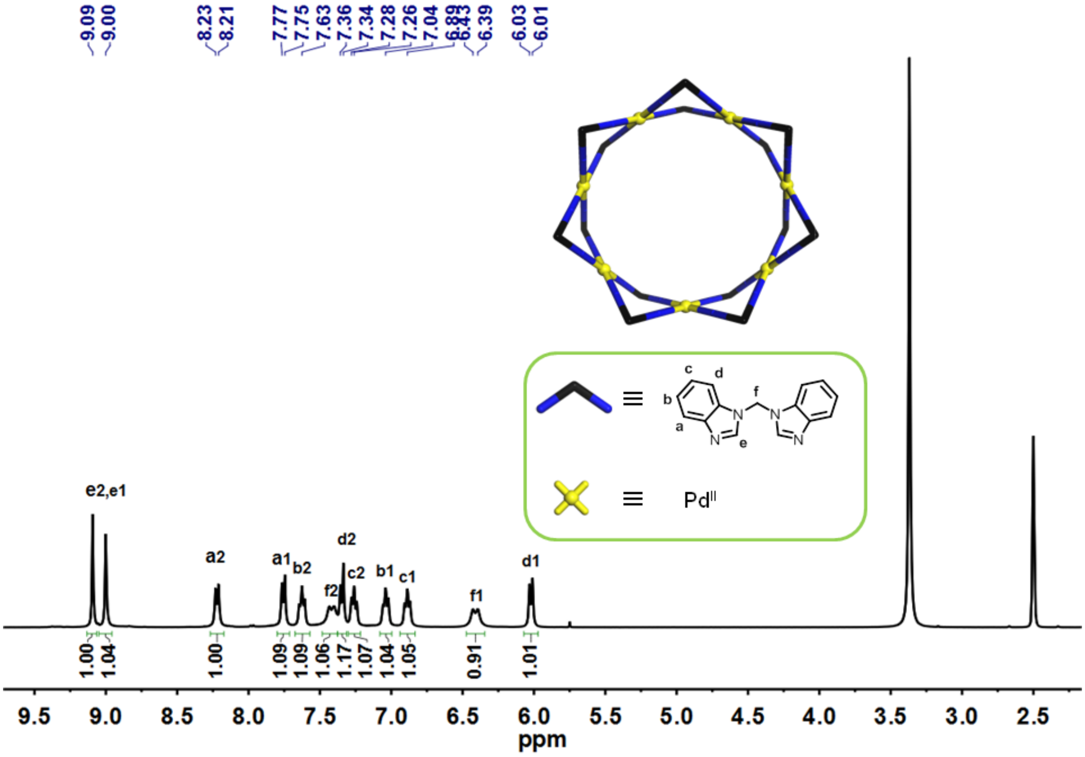

Supplementary Figure 13 ^{1}H NMR spectrum of the $(Pd_7\mathbf{L}_{14})(PF_6)_{14}$ (400 MHz, [D$_6$]DMSO, 298K)**.**

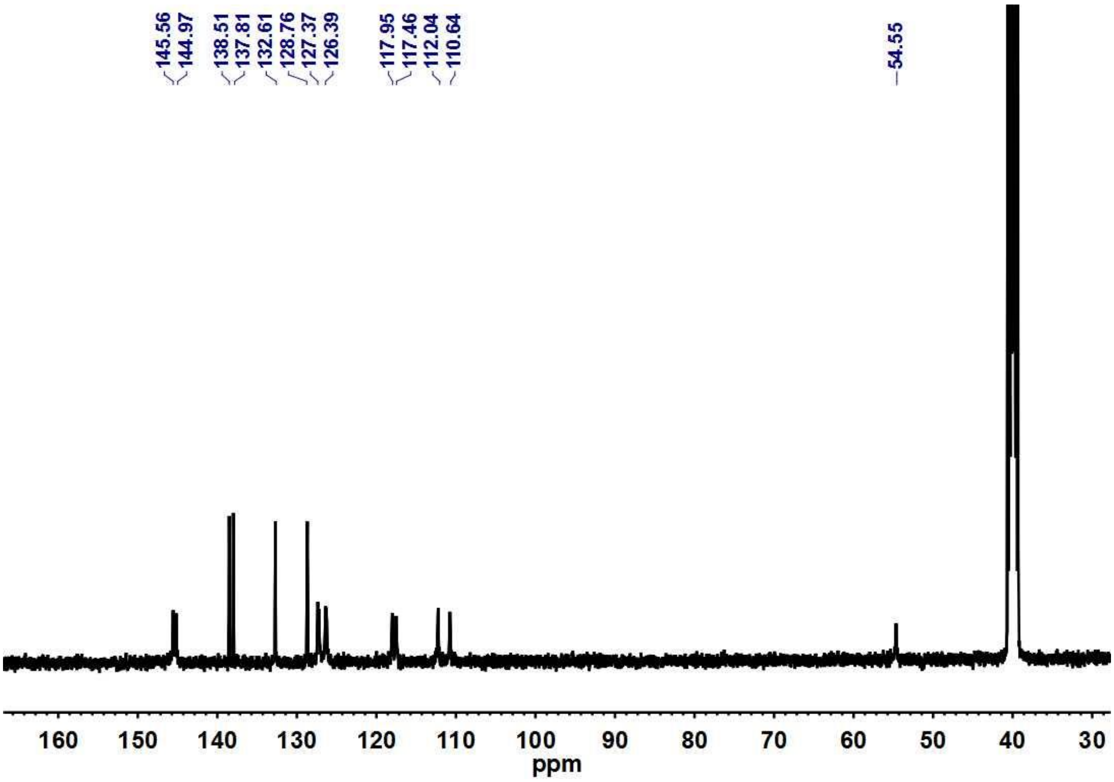

Supplementary Figure 14 ^{13}C NMR spectrum of the $(Pd_7L_{14})(PF_6)_{14}$ (100 MHz, [D$_6$]DMSO, 298K).

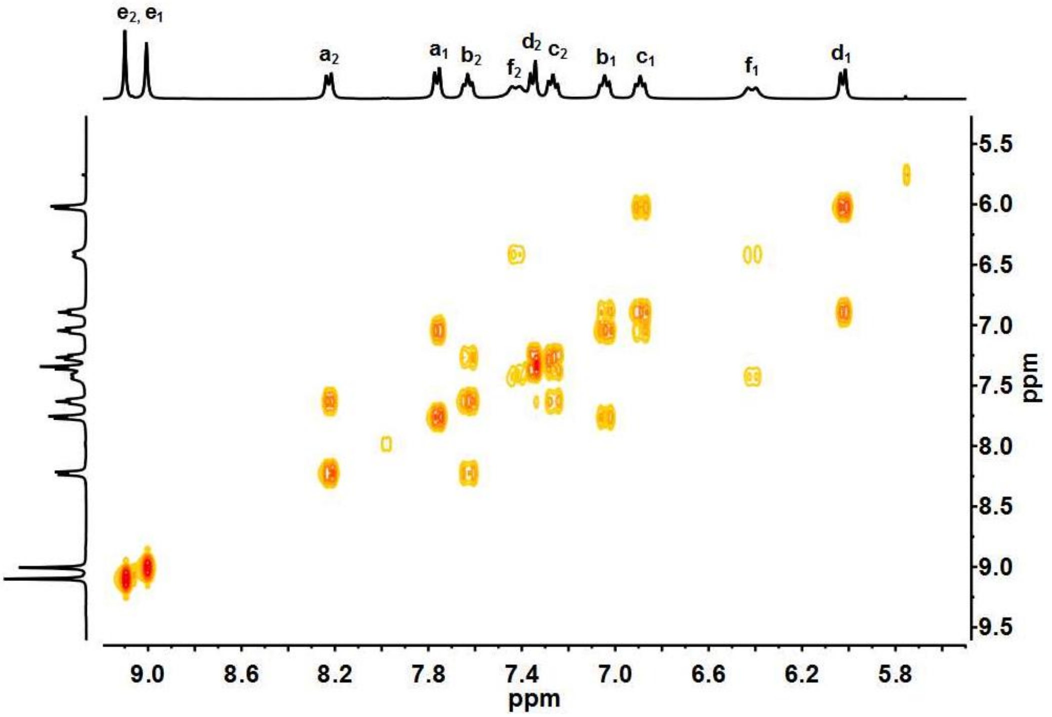

Supplementary Figure 15 ^{1}H-^{1}H COSY NMR spectrum of the $(Pd_7L_{14})(PF_6)_{14}$ (400 MHz, [D6]DMSO, 298K).

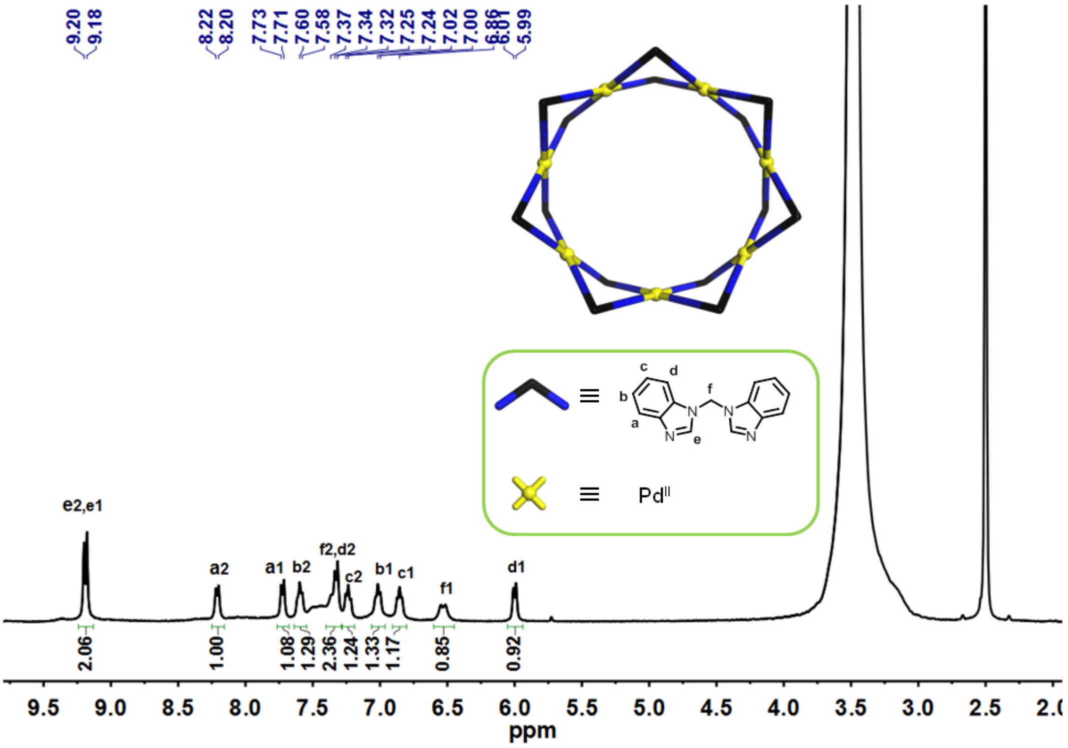

Supplementary Figure 16 ^{1}H NMR spectrum of the $(Pd_7L_{14})(CF_3SO_3)_{14}$ (400 MHz, [D6]DMSO, 298K).

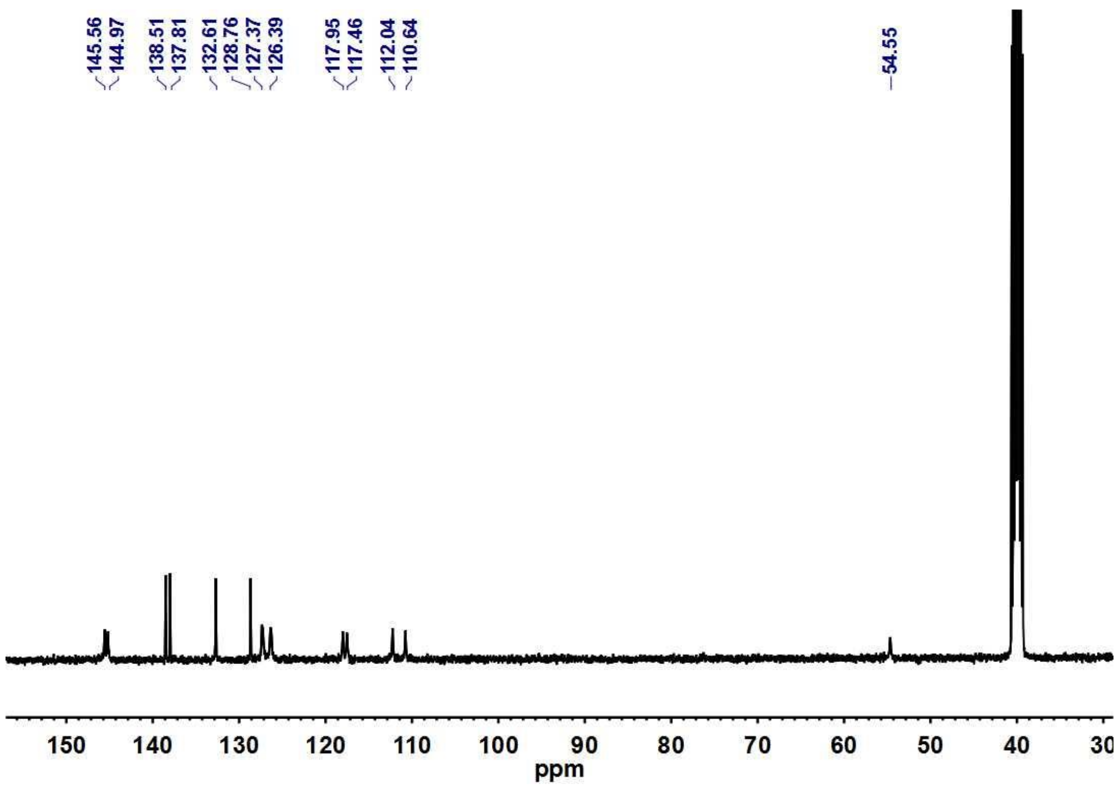

Supplementary Figure 17 ^{13}C NMR spectrum of the $(Pd_7L_{14})(CF_3SO_3)_{14}$ (100 MHz, [D_6]DMSO, 298K).

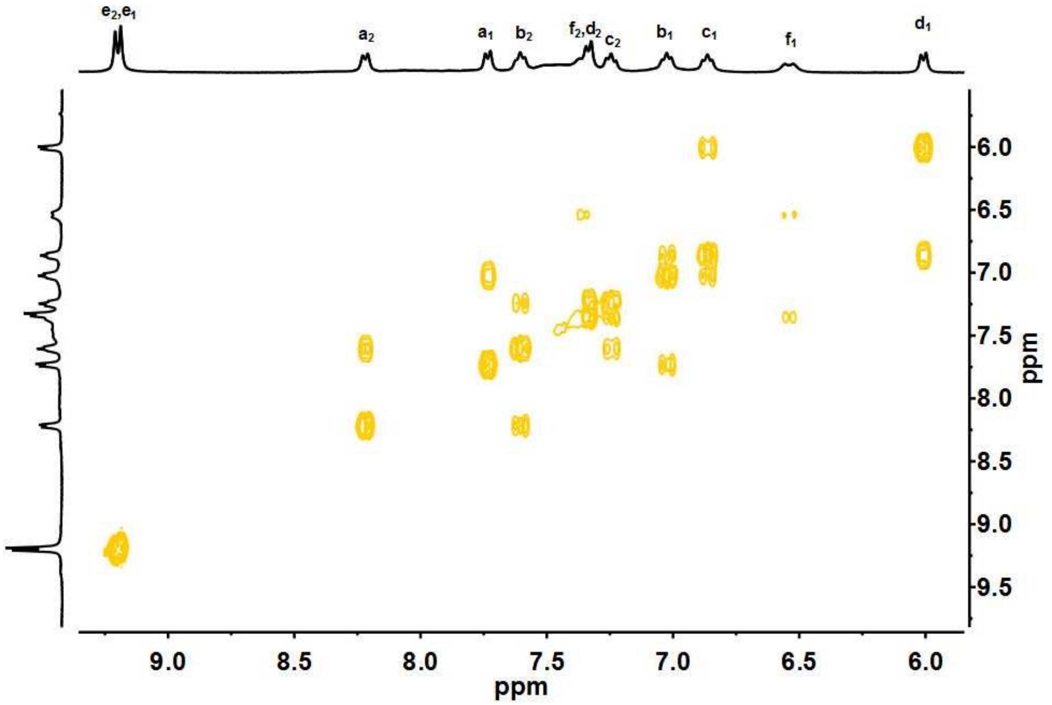

Supplementary Figure 18 ^{1}H-^{1}H COSY NMR spectrum of the $(Pd_7L_{14})(CF_3SO_3)_{14}$ (400MHz, [D_6]DMSO, 298K).

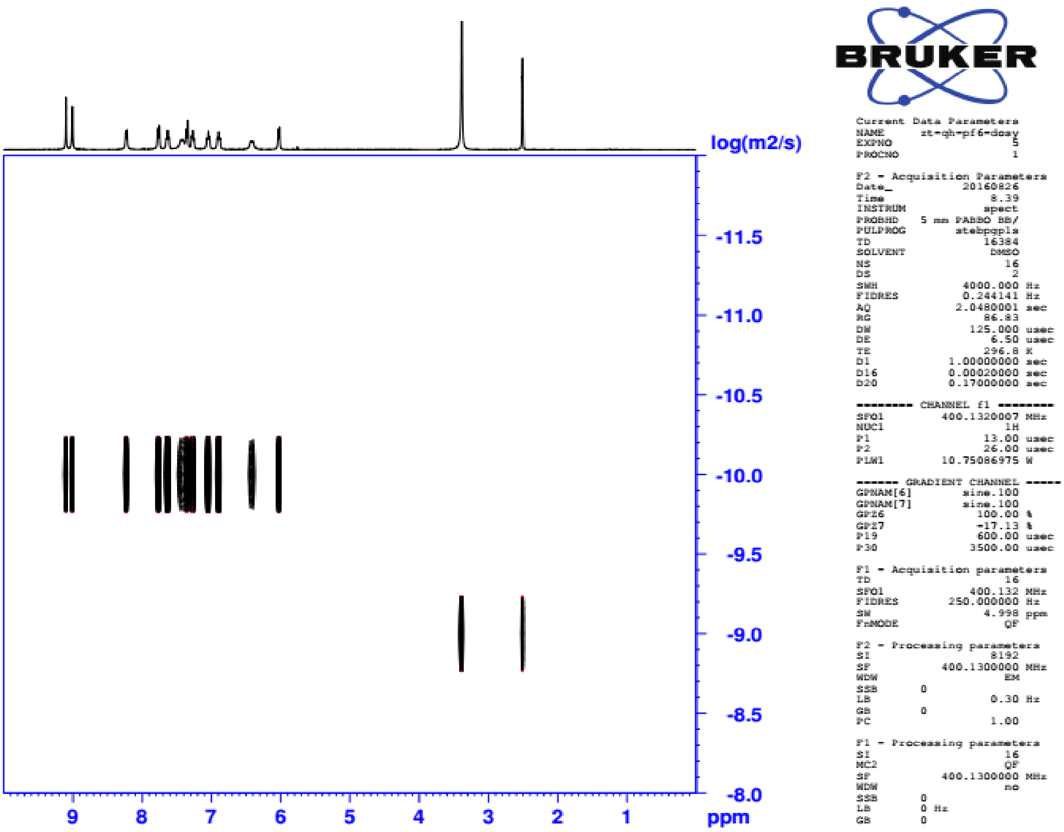

Diffusion Constant = 8.318E-11 m^2/S d=2.38 nm

Supplementary Figure 19 ^{1}H DOSY NMR spectrum of the $(Pd_7L_{14})(PF_6)_{14}$ (400 MHz, $[D_6]$DMSO, 298K).

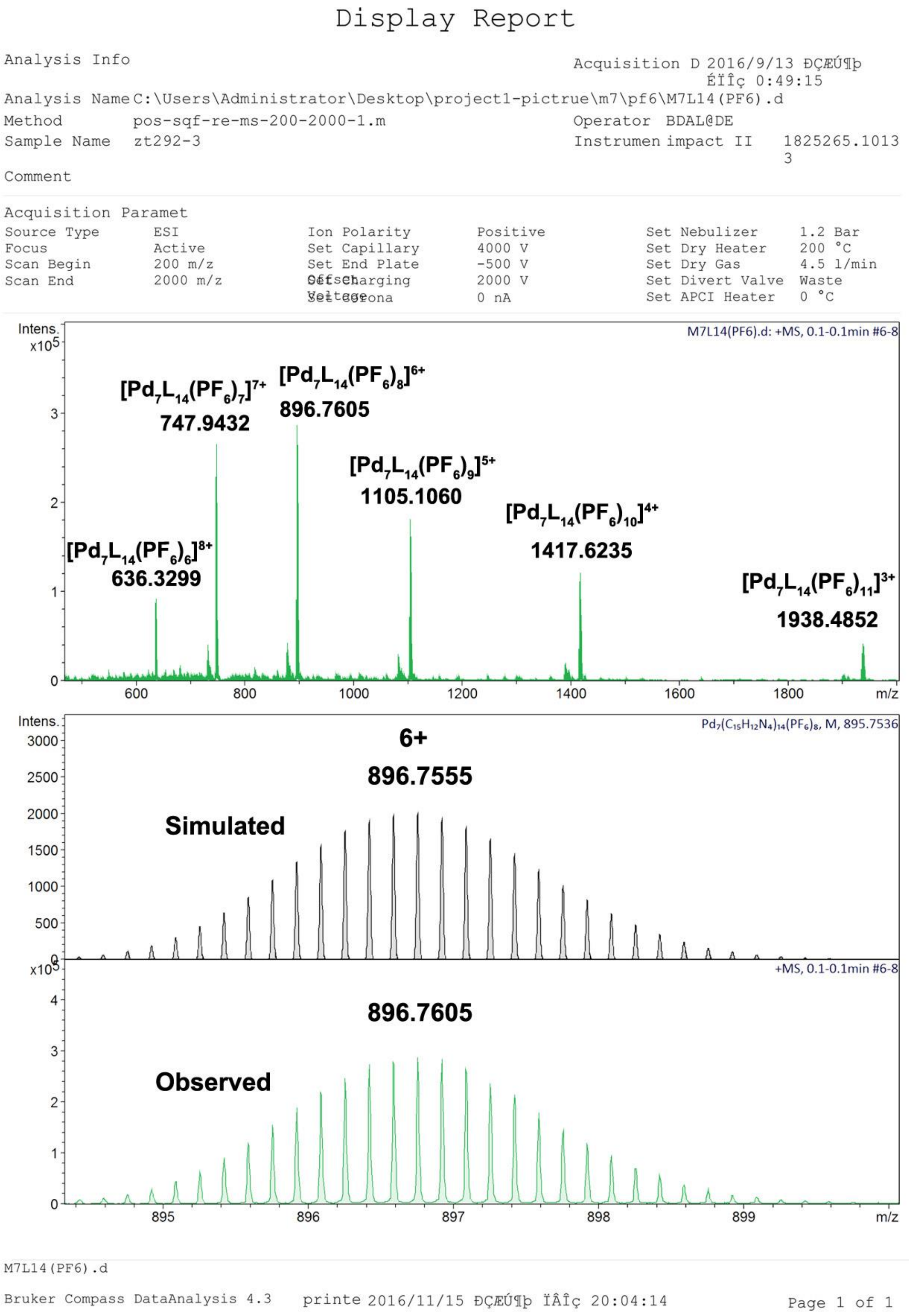

Supplementary Figure 20 ESI-Q-TOF mass spectrum of Pd_7L_{14} (PF_6^- salt) and observed and calculated isotope patterns of 6+

Display Report

Analysis Info				Acquisition D	2015/12/28 ÐÇÆÚÒ» ÉÏÎç 9:23:12
Analysis Name	C:\Users\Administrator\Desktop\project1-pictrue\m7\OTF\ESI+M7L14(OTf).d				
Method	pos_300-3000.m			Operator	YZU
Sample Name				Instrumen	maXis 255552.10164
Comment					

Acquisition Paramet

Source Type	ESI	Ion Polarity	Positive	Set Nebulizer	0.3 Bar
Focus	Not active	Set Capillary	4500 V	Set Dry Heater	180 °C
Scan Begin	300 m/z	Set End Plate Offset	-500 V	Set Dry Gas	4.0 l/min
Scan End	3000 m/z	n/a	n/a	Set Divert Valve	Waste
		Set Corona	0 nA	Set APCI Heater	0 °C

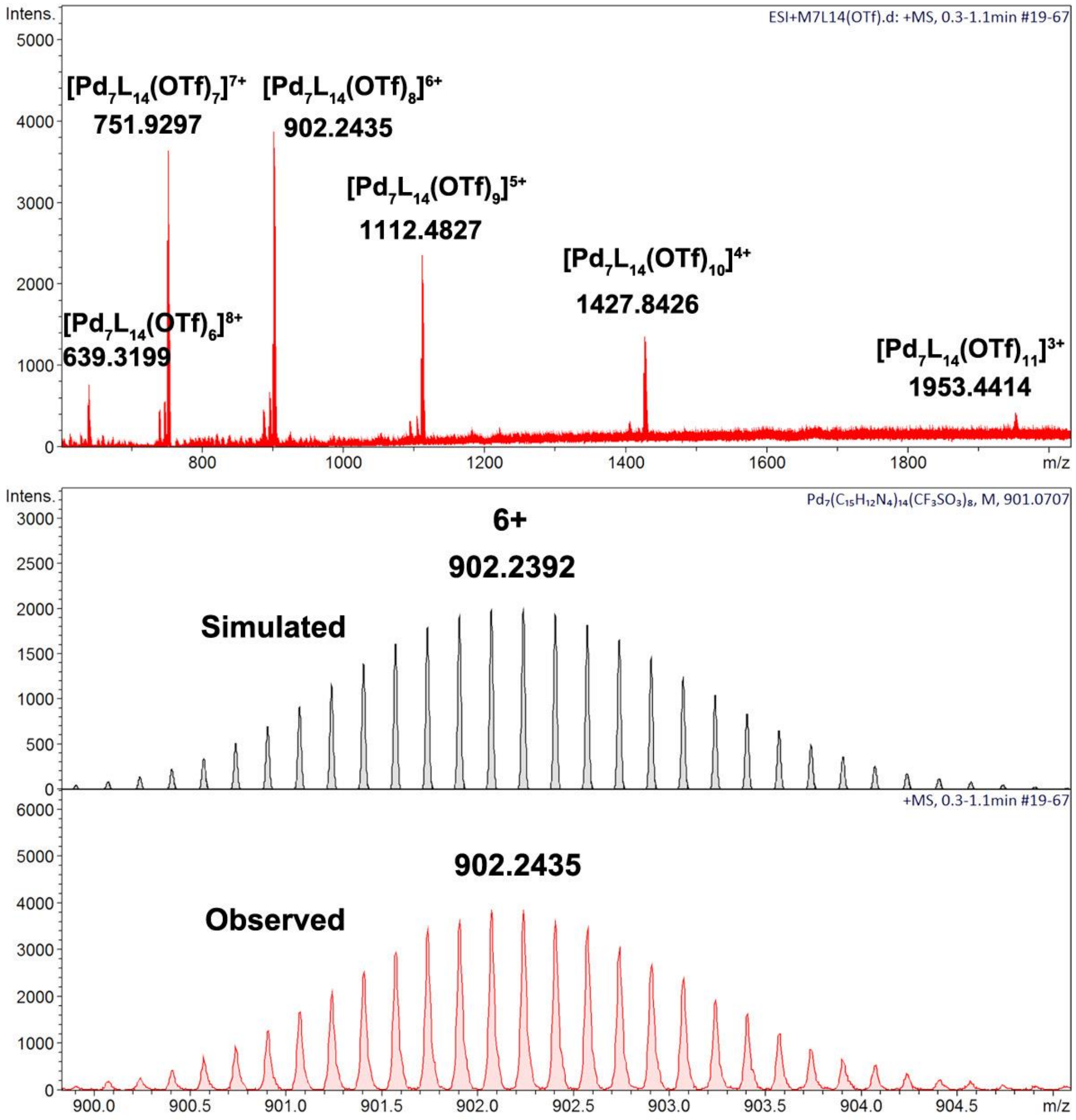

ESI+M7L14(OTf).d

Bruker Compass DataAnalysis 4.3 printe 2016/11/15 ÐÇÆÚ¶þ ÏÂÎç 20:17:18 Page 1 of 1

Supplementary Figure 21 ESI-Q-TOF mass spectrum of Pd_7L_{14} ($CF_3SO_3^-$ salt) and observed and calculated isotope patterns of 6+

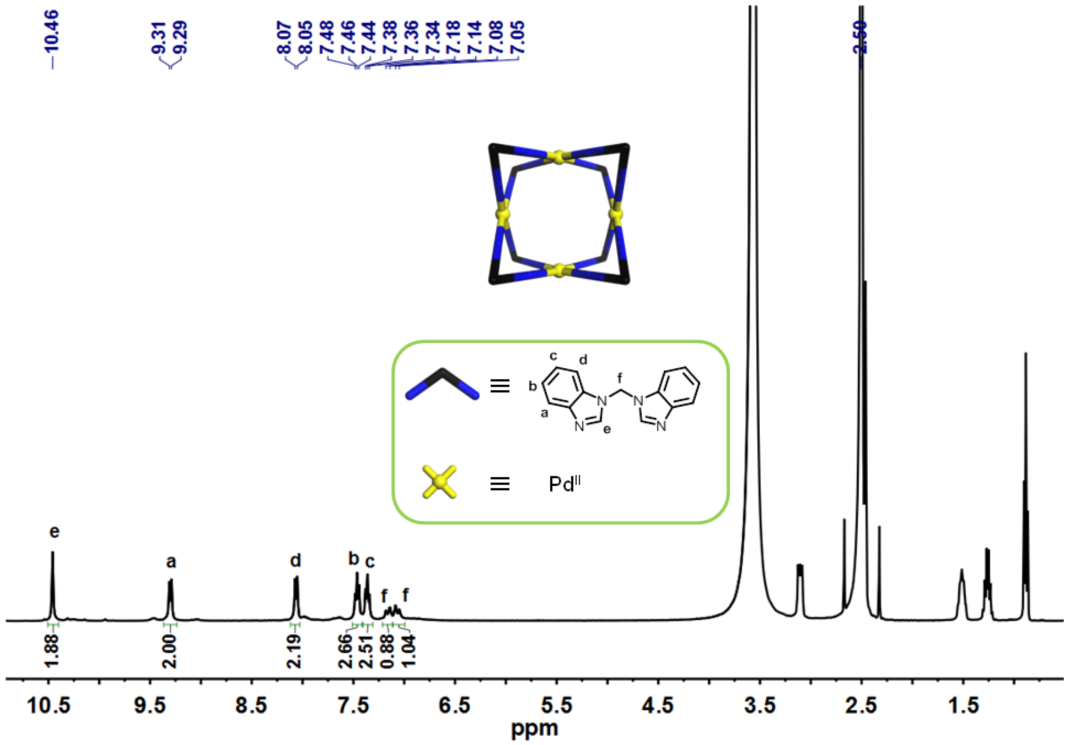

Supplementary Figure 22 ^{1}H NMR spectrum of $(Pd_4\mathbf{L}_8)(SO_4)_2(BF_4)_4$ (400 MHz, $[D_6]$DMSO, 298K).

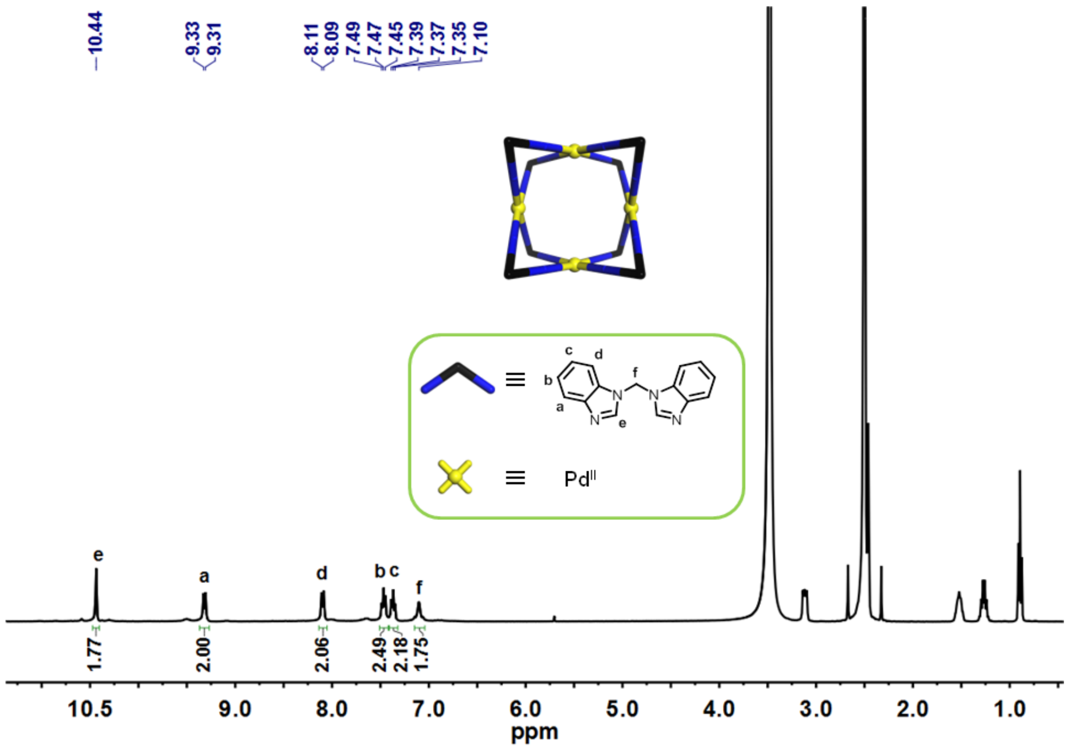

Supplementary Figure 23 ^{1}H NMR spectrum of $(Pd_4L_8)(SO_4)_2(PF_6)_4$ (400 MHz, $[D_6]$DMSO, 298K).

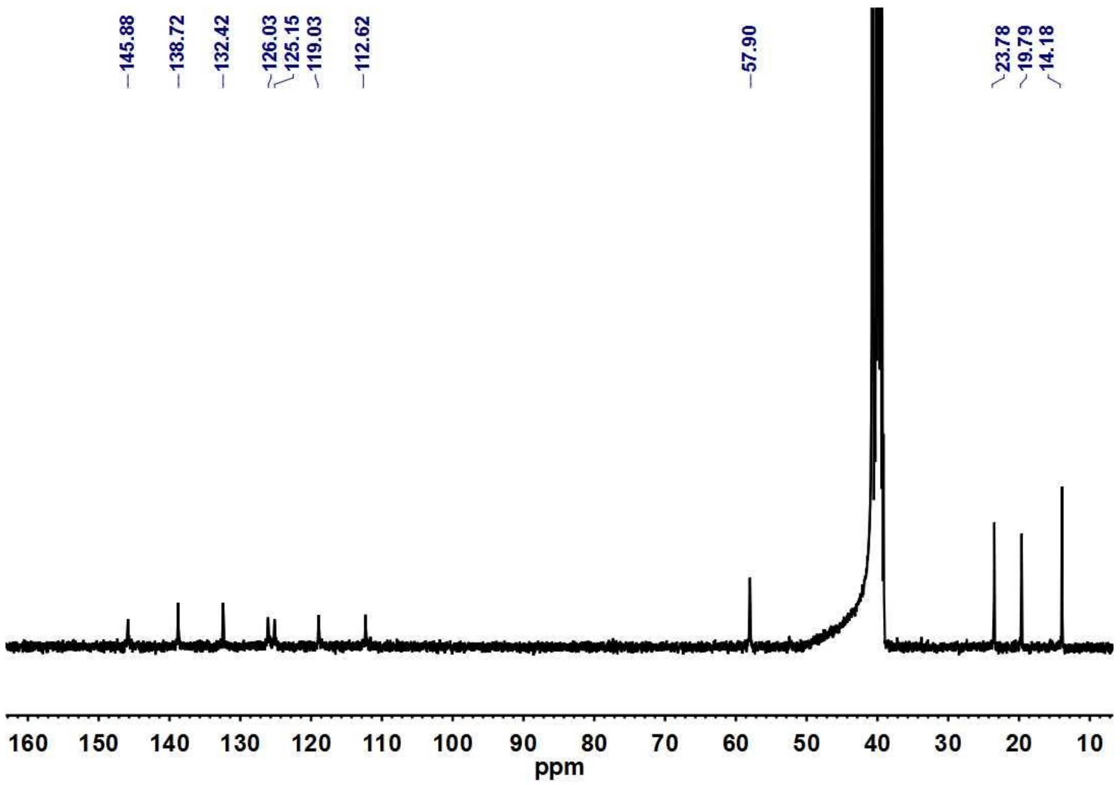

Supplementary Figure 24 ^{13}C NMR spectrum of $(Pd_4L_8)(SO_4)_2(PF_6)_4$ (100 MHz, $[D_6]$DMSO, 298K).

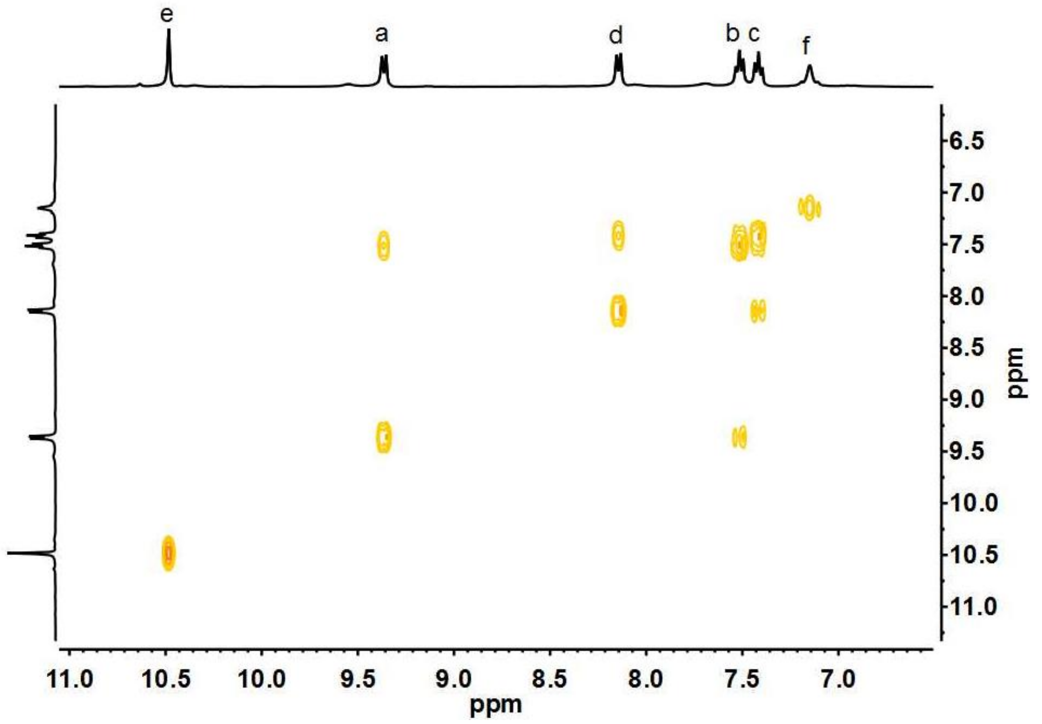

Supplementary Figure 25 ^1H-^1H COSY NMR spectrum of $Pd_4L_8(SO_4)_2(PF_6)_4$ (400 MHz, $[D_6]$DMSO, 298K).

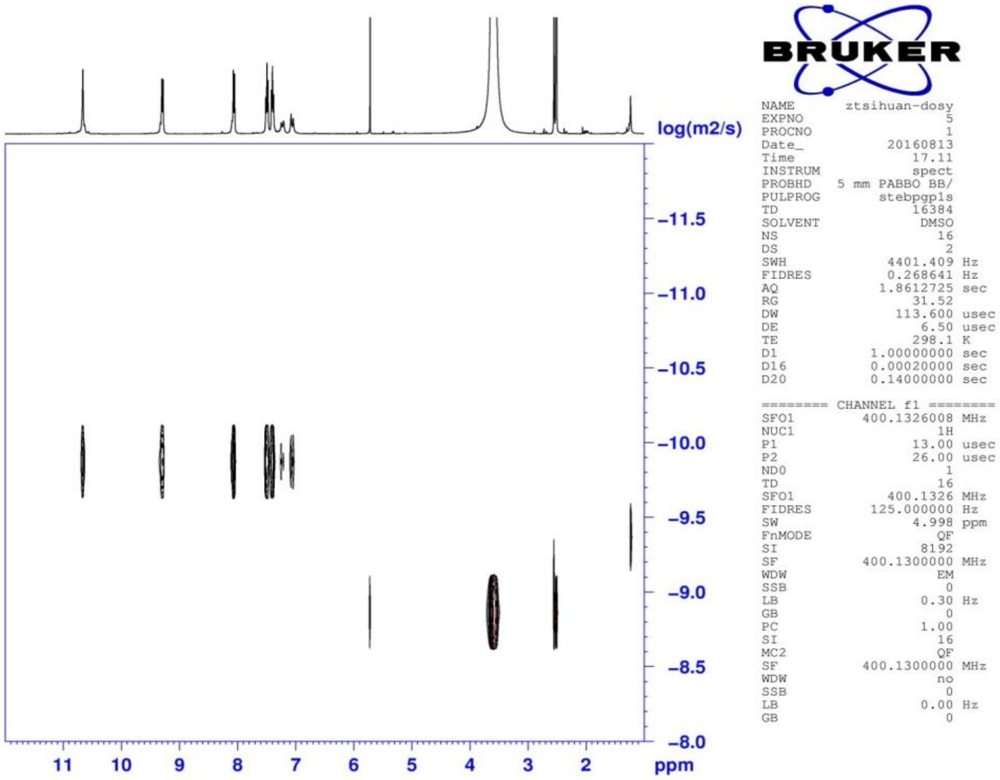

Diffusion Constant = 1.309E-10 m^2/S d=1.51 nm

Supplementary Figure 26 ^{1}H DOSY spectrum of the ($Pd_4$$\mathbf{L}_8$)(SO_4)$_2(BF_4)_4$ (400 MHz, [D_6]DMSO, 298K)**.**

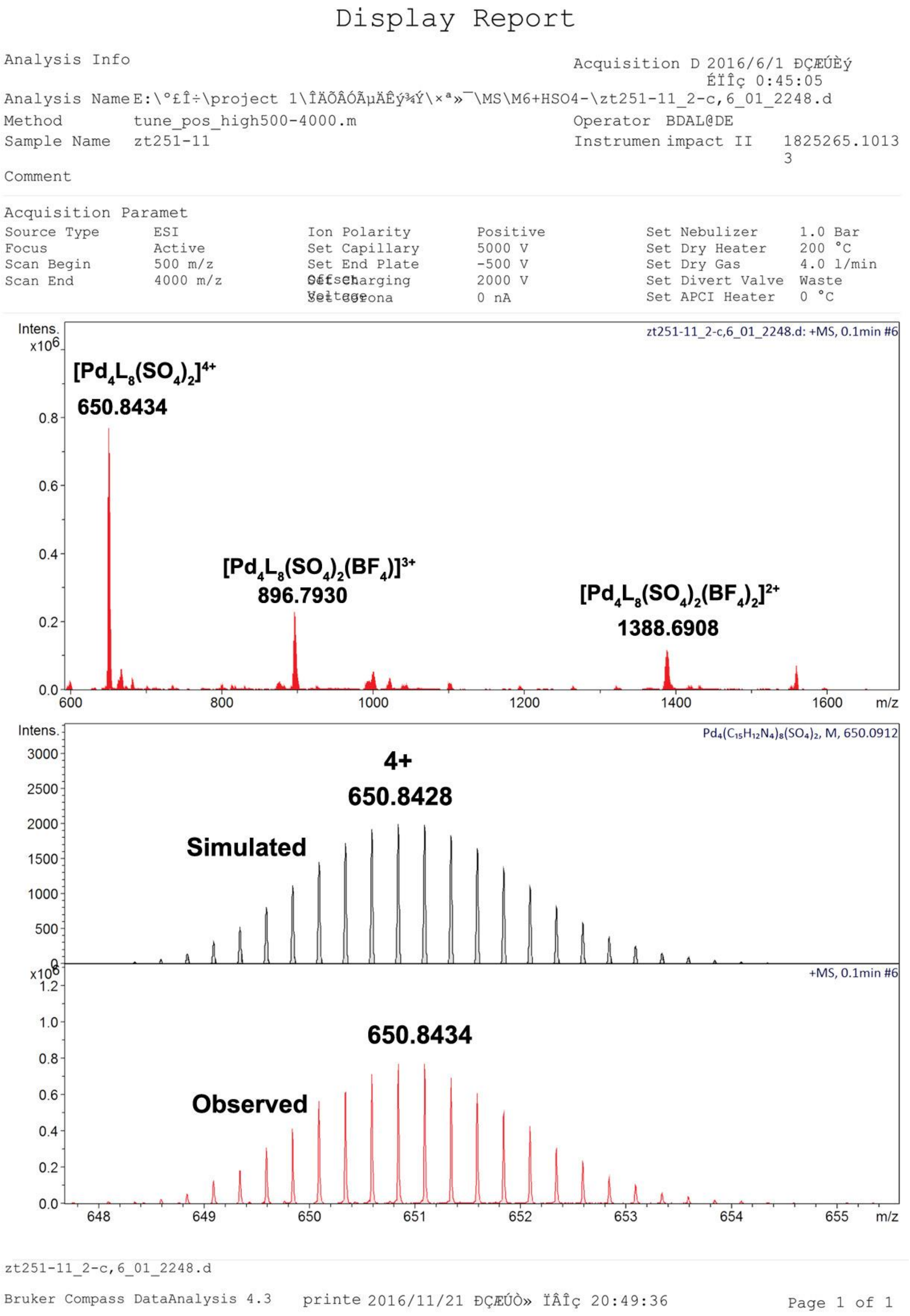

Supplementary Figure 27 ESI-Q-TOF mass spectrum of complex $Pd_6L_{12}(BF_4)_{12}$ after the addition of 4.5 eq. of $N(C_4H_9)_4HSO_4$ and observed and calculated isotope patterns of 4+

Display Report

Analysis Info		Acquisition D	2016/11/21 ĐÇÆÚÒ» ÏÂÎç 20:58:25
Analysis Name	C:\Users\Administrator\Desktop\project1-pictrue\Zt330-4M7+HSO4.d		
Method	pos-sqf-re-ms-200-2000-1.m	Operator	BDAL@DE
Sample Name	Zt330-4	Instrumen	impact II 1825265.10133
Comment			

Acquisition Paramet

Source Type	ESI	Ion Polarity	Positive	Set Nebulizer	1.2 Bar
Focus	Active	Set Capillary	4000 V	Set Dry Heater	200 °C
Scan Begin	200 m/z	Set End Plate Offset	-500 V	Set Dry Gas	4.5 l/min
Scan End	2000 m/z	Set Charging Voltage	2000 V	Set Divert Valve	Waste
		Set Corona	0 nA	Set APCI Heater	0 °C

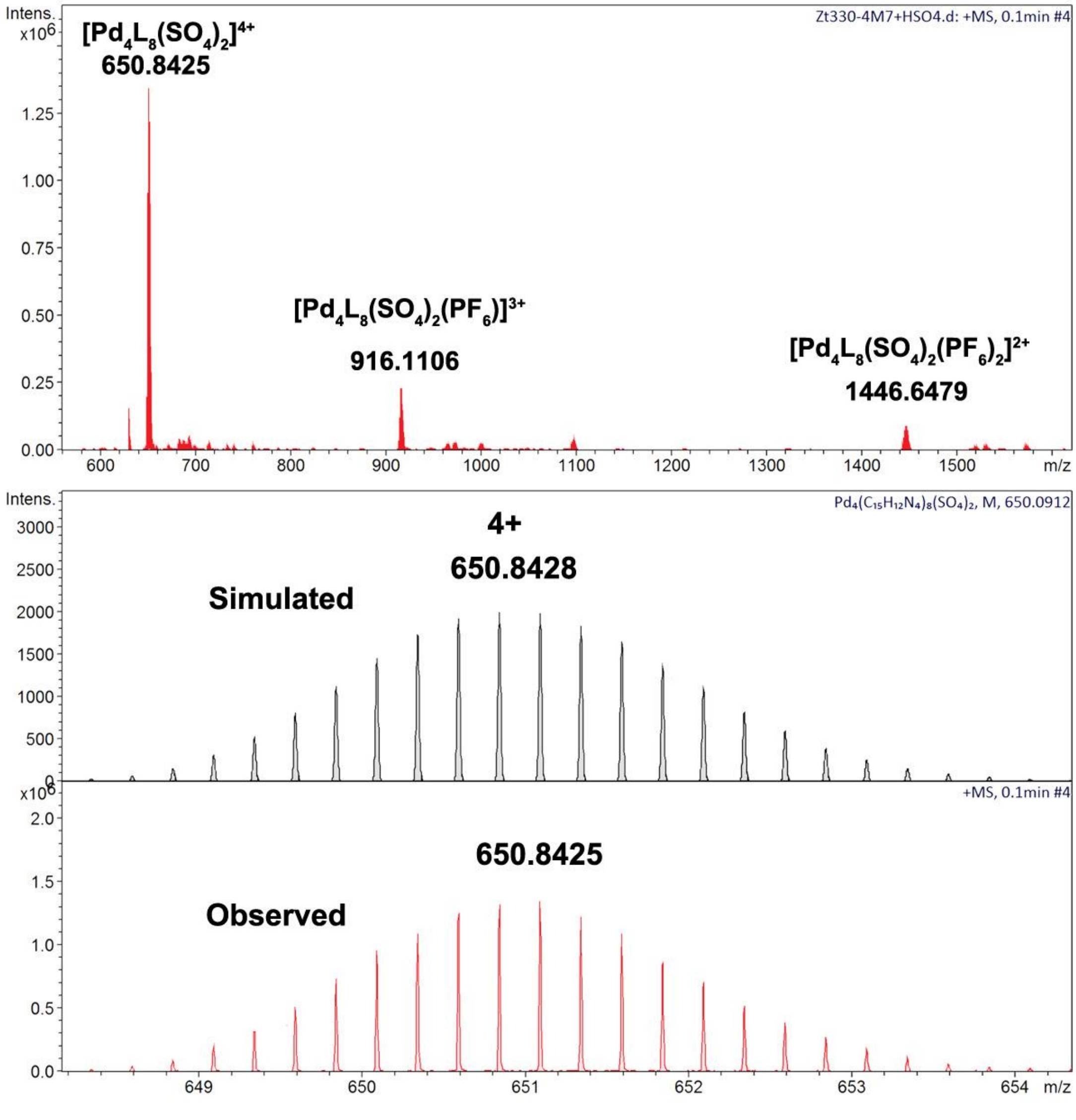

Zt330-4M7+HSO4.d

Bruker Compass DataAnalysis 4.3 printe 2016/11/22 ĐÇÆÚ¶þ ÏÂÎç 15:25:17 Page 1 of 1

Supplementary Figure 28 ESI-Q-TOF mass spectrum of complex $Pd_7L_{14}(PF_6)_{14}$ after the addition 5.25 eq. of $N(C_4H_9)_4HSO_4$ and observed and calculated isotope patterns of 4+.

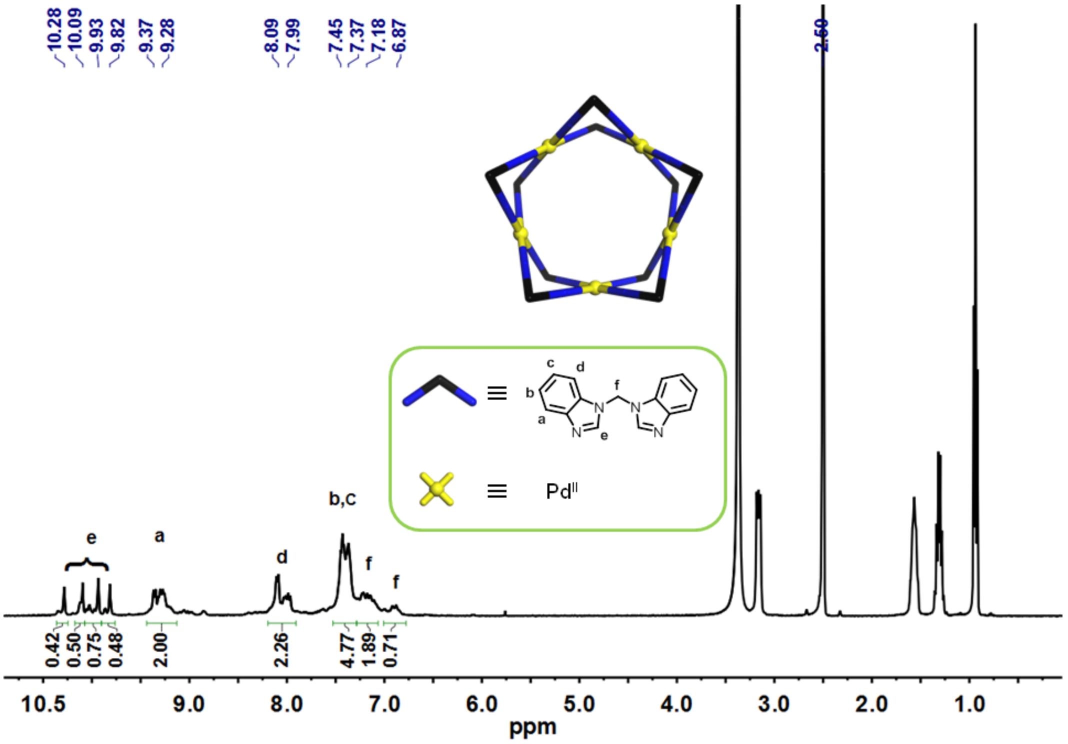

Supplementary Figure 29 ^{1}H NMR spectrum of $Pd_5L_{10}(HMo_7O_{24})(BF_4)_5$ (400 MHz, [D_6]DMSO, 298K).

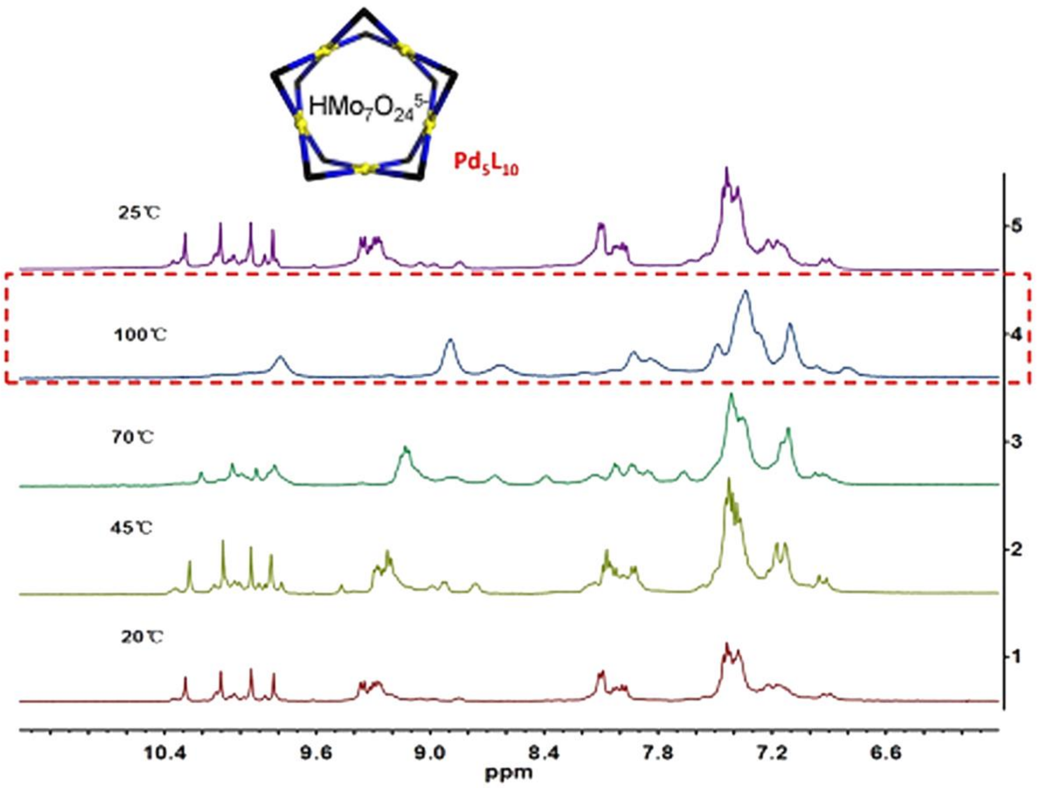

Supplementary Figure 30 ^{1}H VT-NMR spectrum of $Pd_5L_{10}(HMo_7O_{24})(BF_4)_5$ (400 MHz, [D_6]DMSO, 293-373-298K).

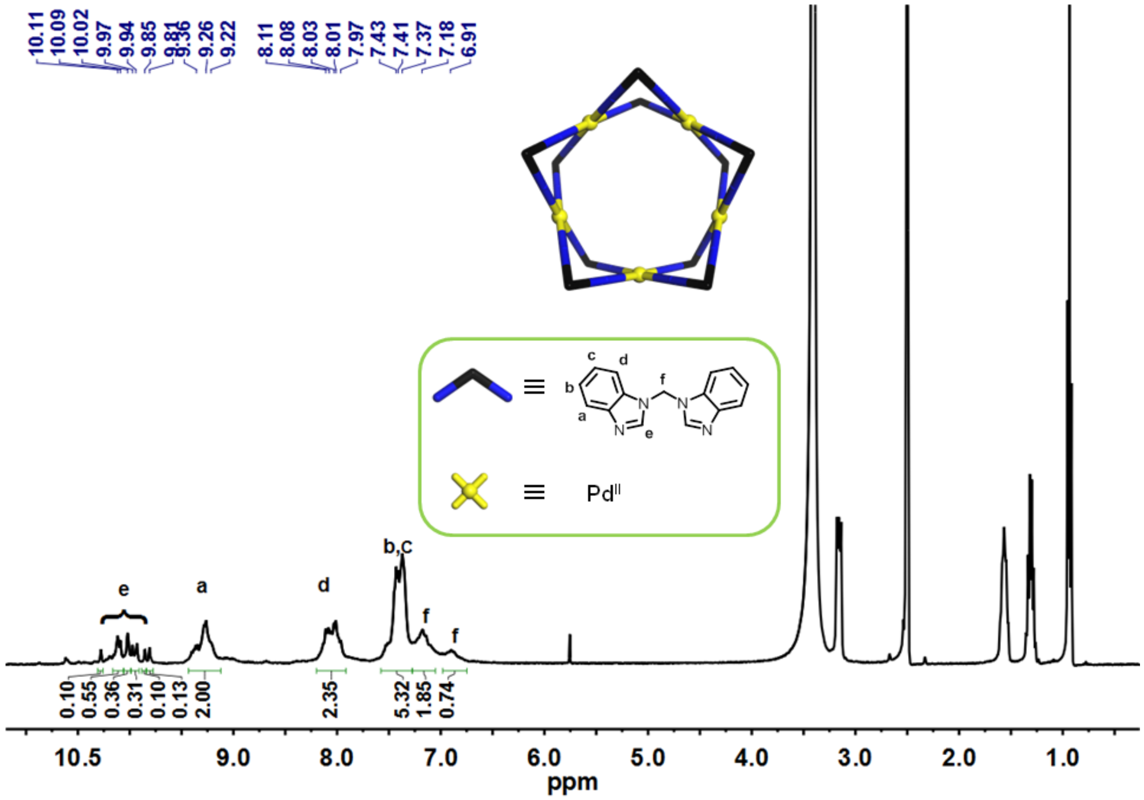

Supplementary Figure 31 ^{1}H NMR spectrum of the $Pd_5\mathbf{L}_{10}(HMo_7O_{24})(PF_4)_5$ (400 MHz, [D_6]DMSO, 298K).

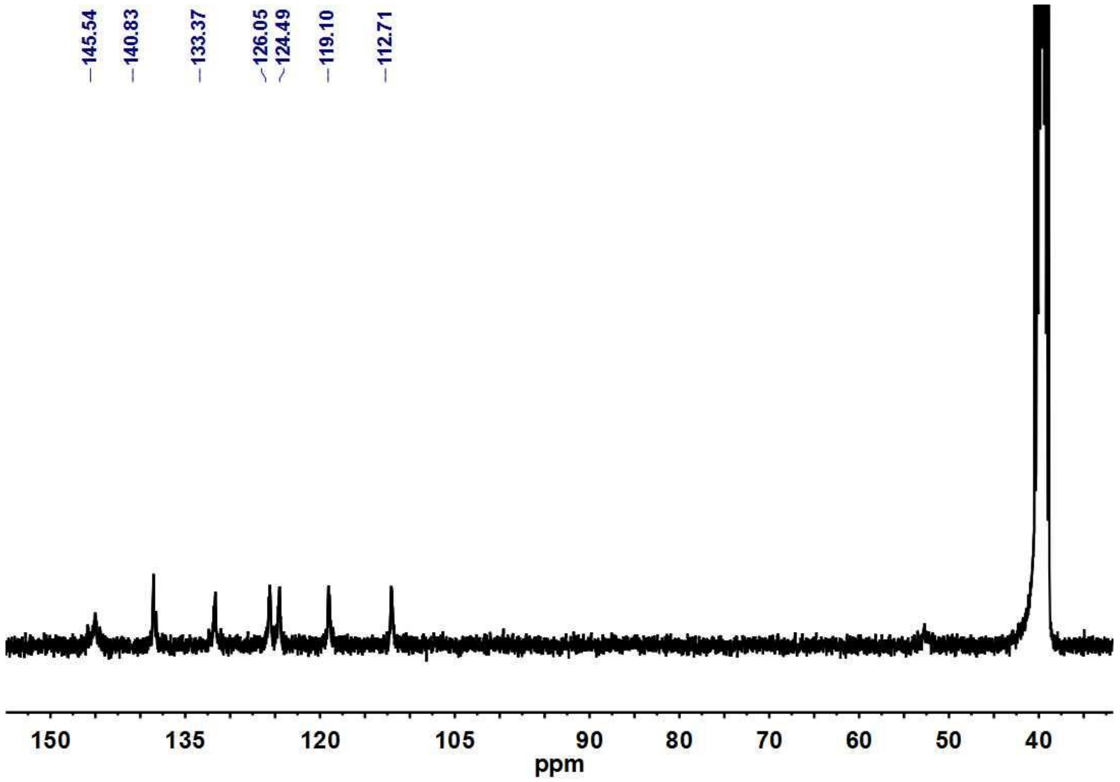

Supplementary Figure 32 ^{13}C NMR spectrum of the $(Pd_5L_{10})(HMo_7O_{24})(BF_4)_5$ (100 MHz, [D_6]DMSO, 298K).

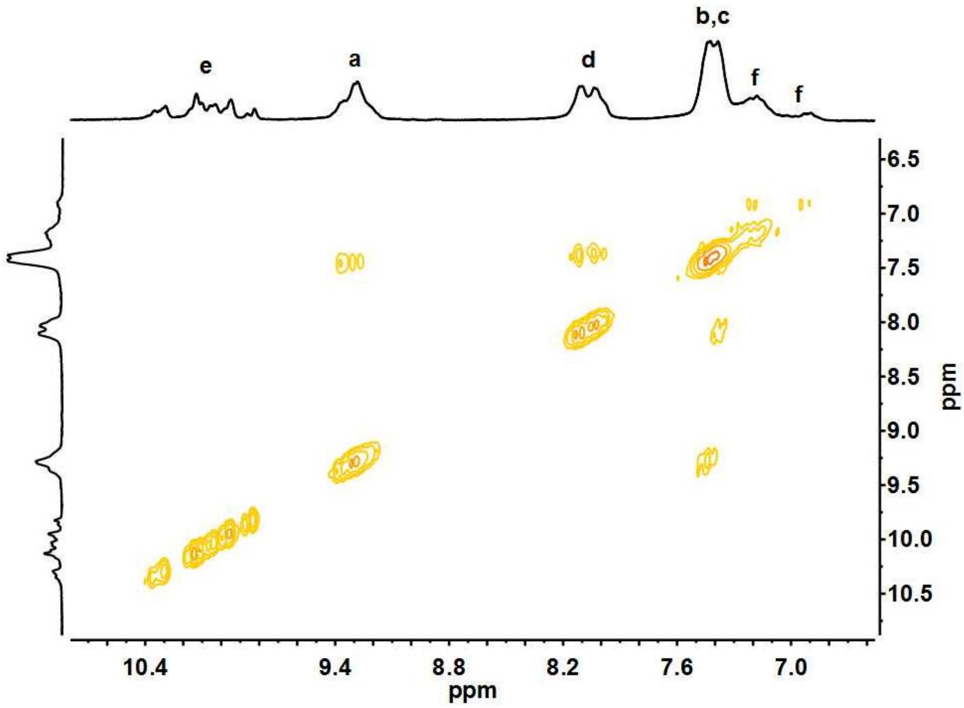

Supplementary Figure 33 ^1H-^1H COSY NMR spectrum of the $Pd_5L_{10}(HMo_7O_{24})(PF_4)_5$ (400 MHz, $[D_6]DMSO$, 298K).

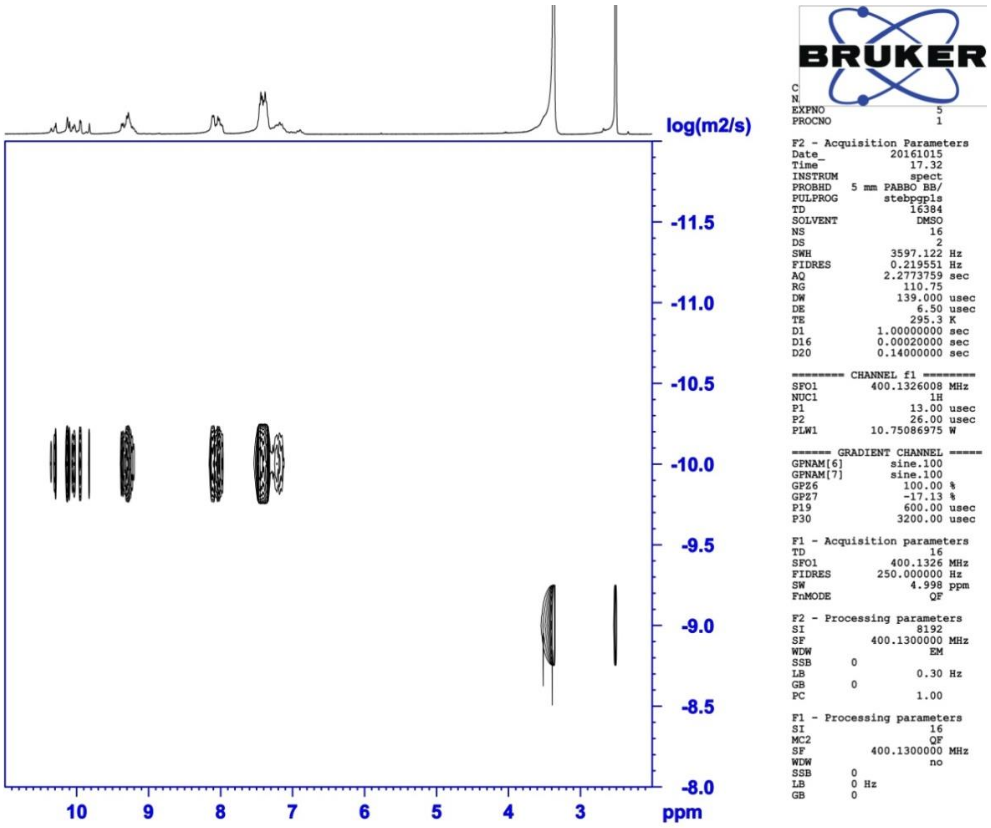

Diffusion Constant = 1.000E-10 m²/S d=1.84 nm

Supplementary Figure 34 ^1H DOSY spectrum of the $(Pd_5L_{10})(HMo_7O_{24})(PF_6)_5$ (400 MHz, $[D_6]DMSO$, 298K).

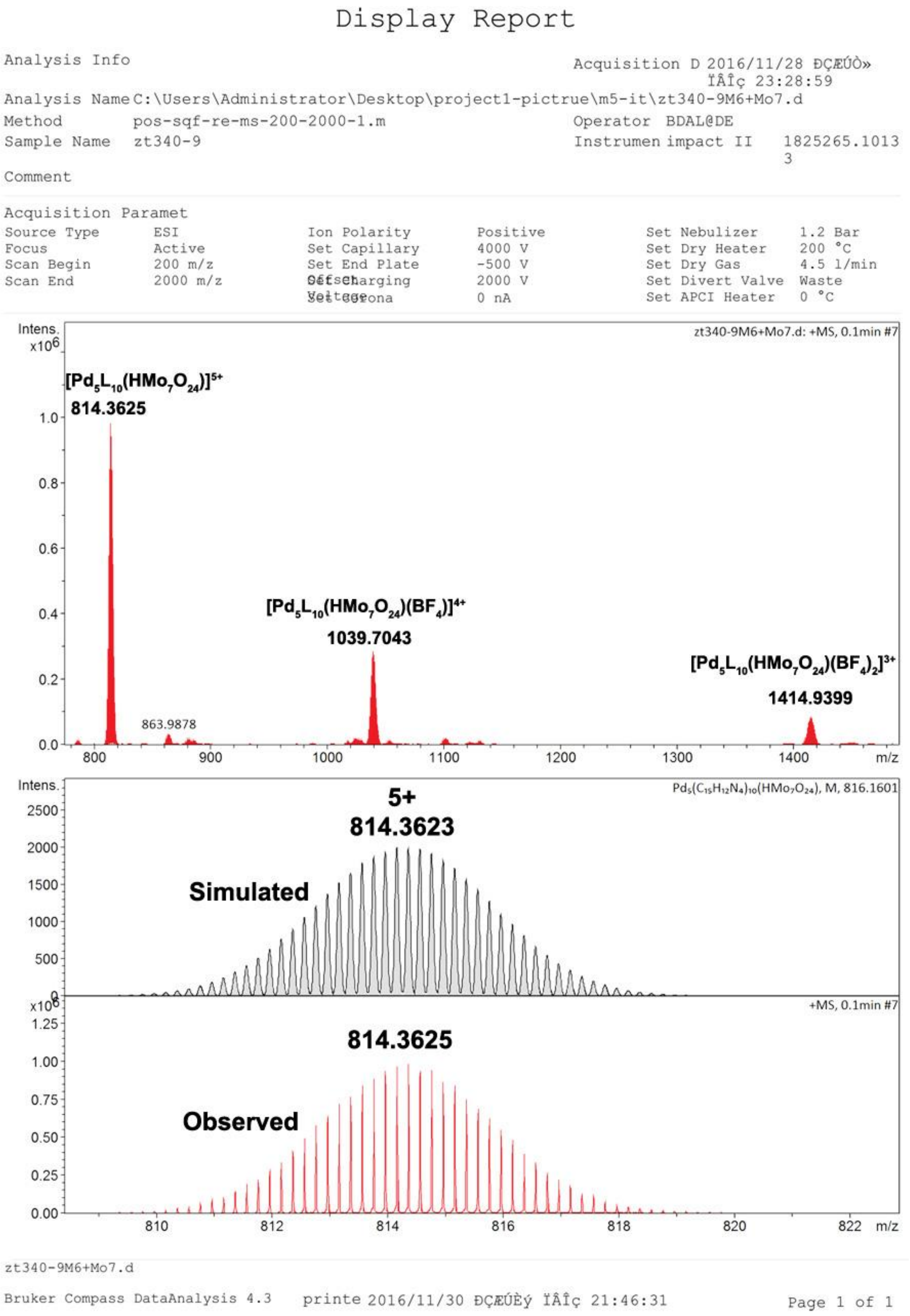

Supplementary Figure 35 ESI-Q-TOF mass spectrum of complex $(Pd_6L_{12})(BF_4)_{12}$ after the addition 0.8 eq. of $[N(C_4H_9)_4]_6Mo_7O_{24}$ and observed and calculated isotope patterns of 5+.

Display Report

Analysis Info

Acquisition D 2016/11/21 ÐÇÆÚÒ» ÏÂÎç 21:11:46

Analysis Name	C:\Users\Administrator\Desktop\project1-pictrue\Zt331-15M7+Mo7.d		
Method	pos-sqf-re-ms-200-2000-1.m	Operator	BDAL@DE
Sample Name	Zt331-15	Instrumen	impact II 1825265.10133
Comment			

Acquisition Paramet

Source Type	ESI	Ion Polarity	Positive	Set Nebulizer	1.2 Bar
Focus	Active	Set Capillary	4000 V	Set Dry Heater	200 °C
Scan Begin	200 m/z	Set End Plate Offset	-500 V	Set Dry Gas	4.5 l/min
Scan End	2000 m/z	Set Charging Voltage	2000 V	Set Divert Valve	Waste
		Set Corona	0 nA	Set APCI Heater	0 °C

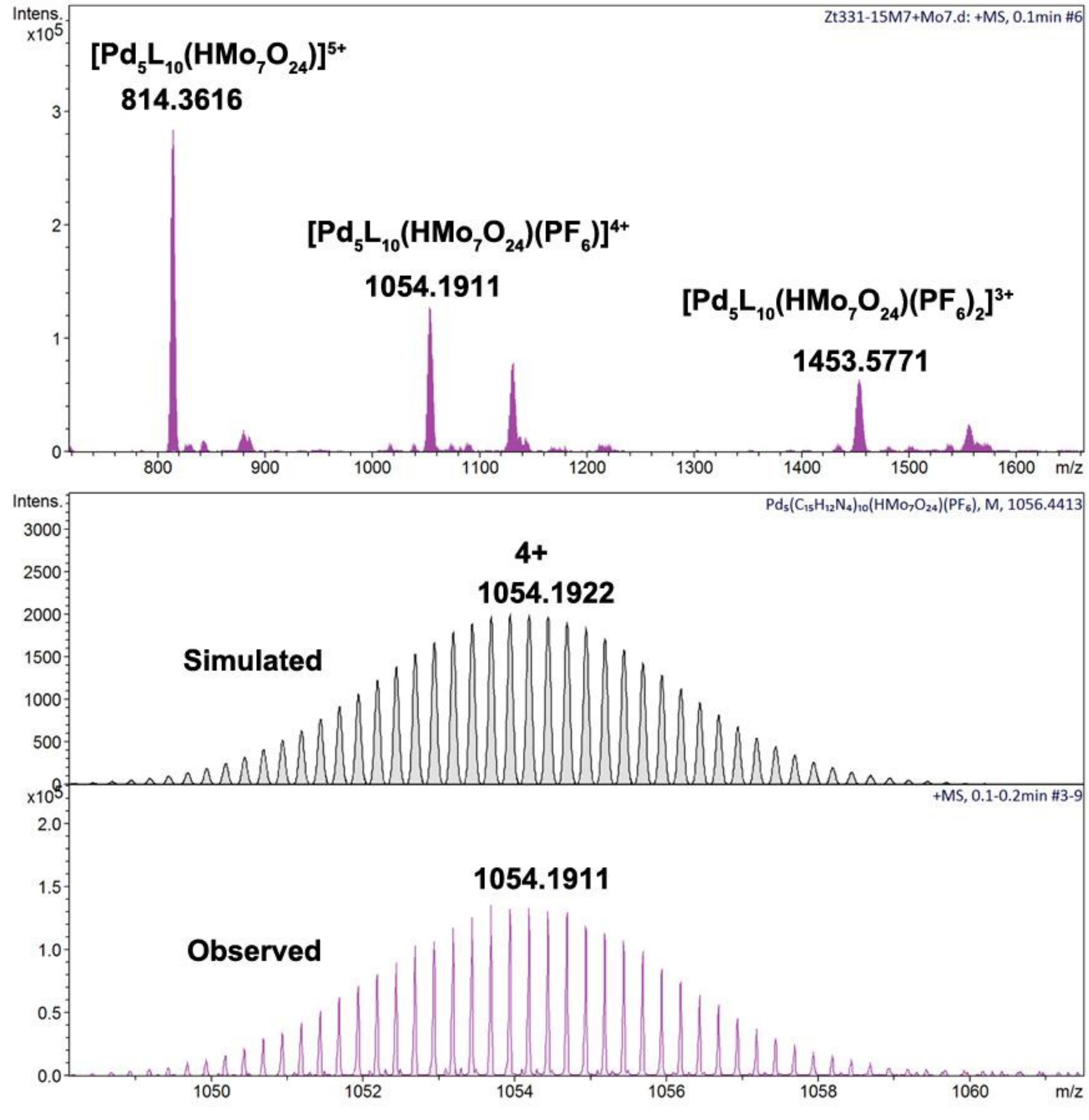

Zt331-15M7+Mo7.d

Bruker Compass DataAnalysis 4.3 printe 2016/11/22 ÐÇÆÚ¶þ ÏÂÎç 15:53:55 Page 1 of 1

Supplementary Figure 36 ESI-Q-TOF mass spectrum of complex $(Pd_7L_{14})(PF_6)_{14}$ after the addition 0.8 eq. of $[N(C_4H_9)_4]_6Mo_7O_{24}$ and observed and calculated isotope patterns of 4+.

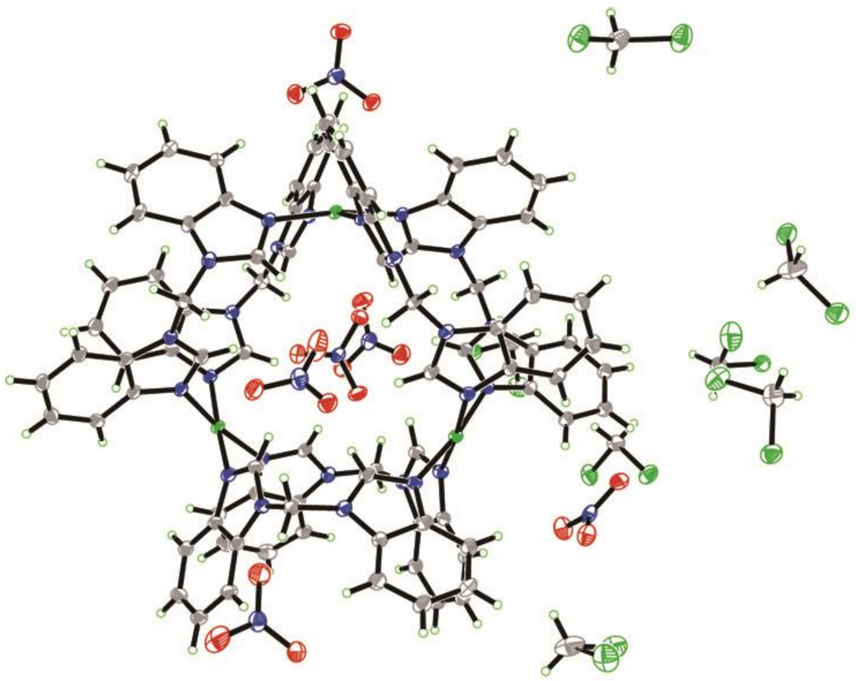

Supplementary Figure 37 Ortep-drawing of the asymmetric unit in the crystal structure of Pd_3L_6 at 30% probability level

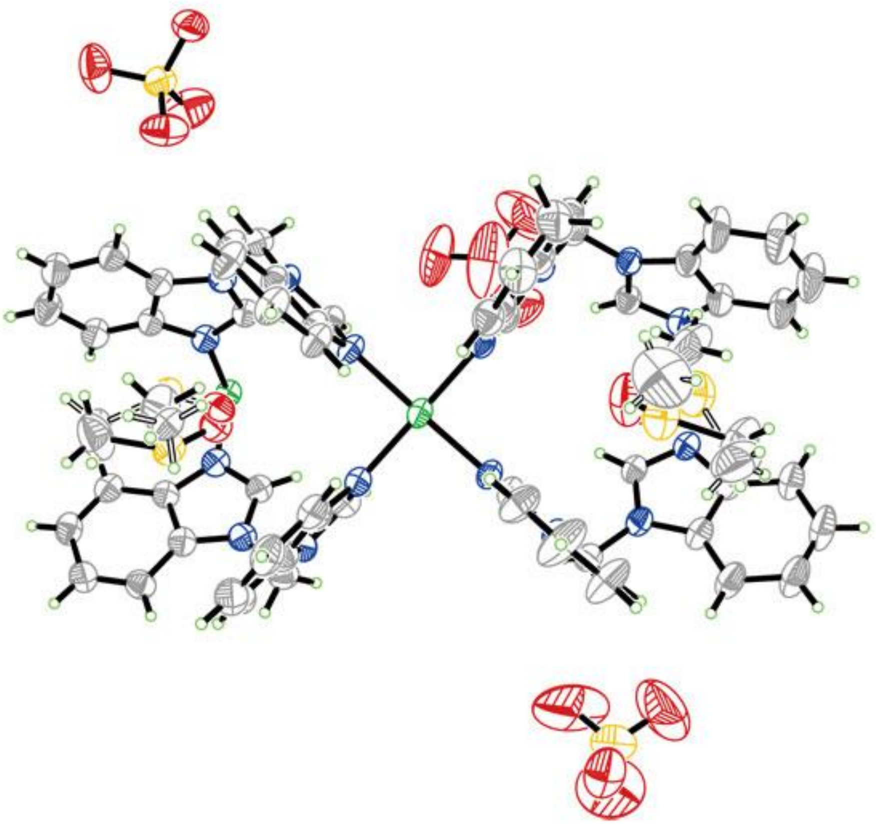

Supplementary Figure 38 Ortep drawing of the asymmetric unit in the crystal structure of $Pd_4L_8(SO_4^{2-}$ salt) at 30% probability level

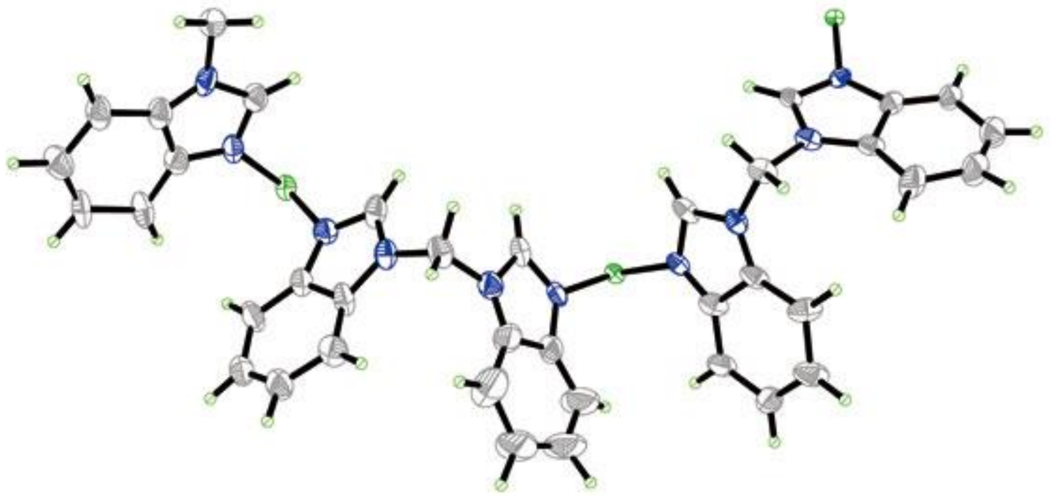

Supplementary Figure 39 Ortep drawing of the asymmetric unit in the crystal structure of Pd_5L_{10} at 30% probability level

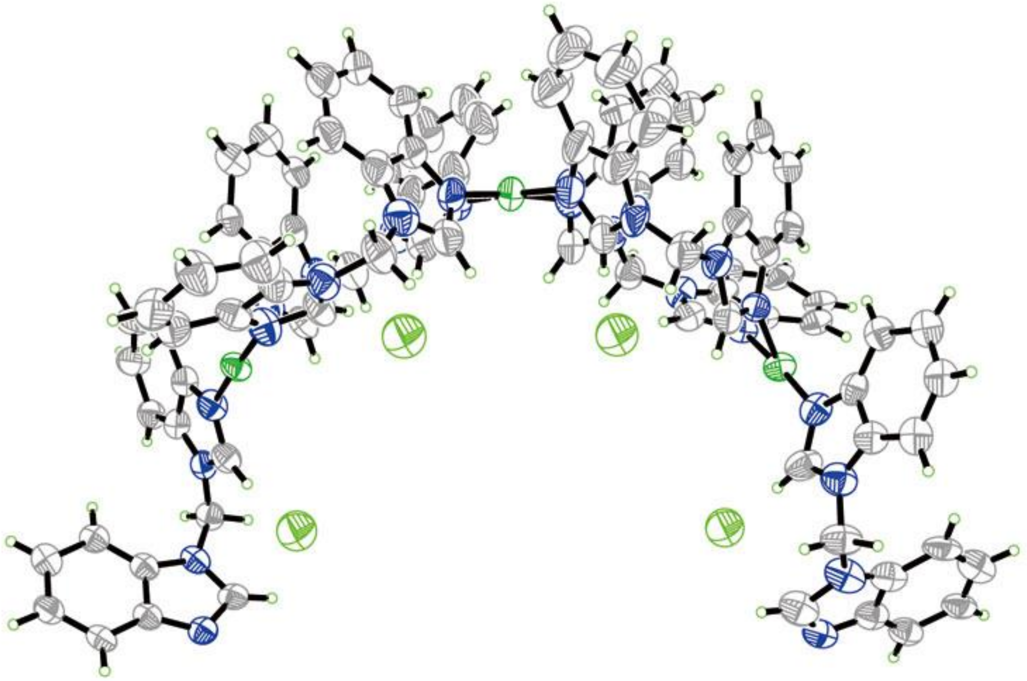

Supplementary Figure 40 Ortep drawing of the asymmetric unit in the crystal structure of Pd_6L_{12} at 30% probability level

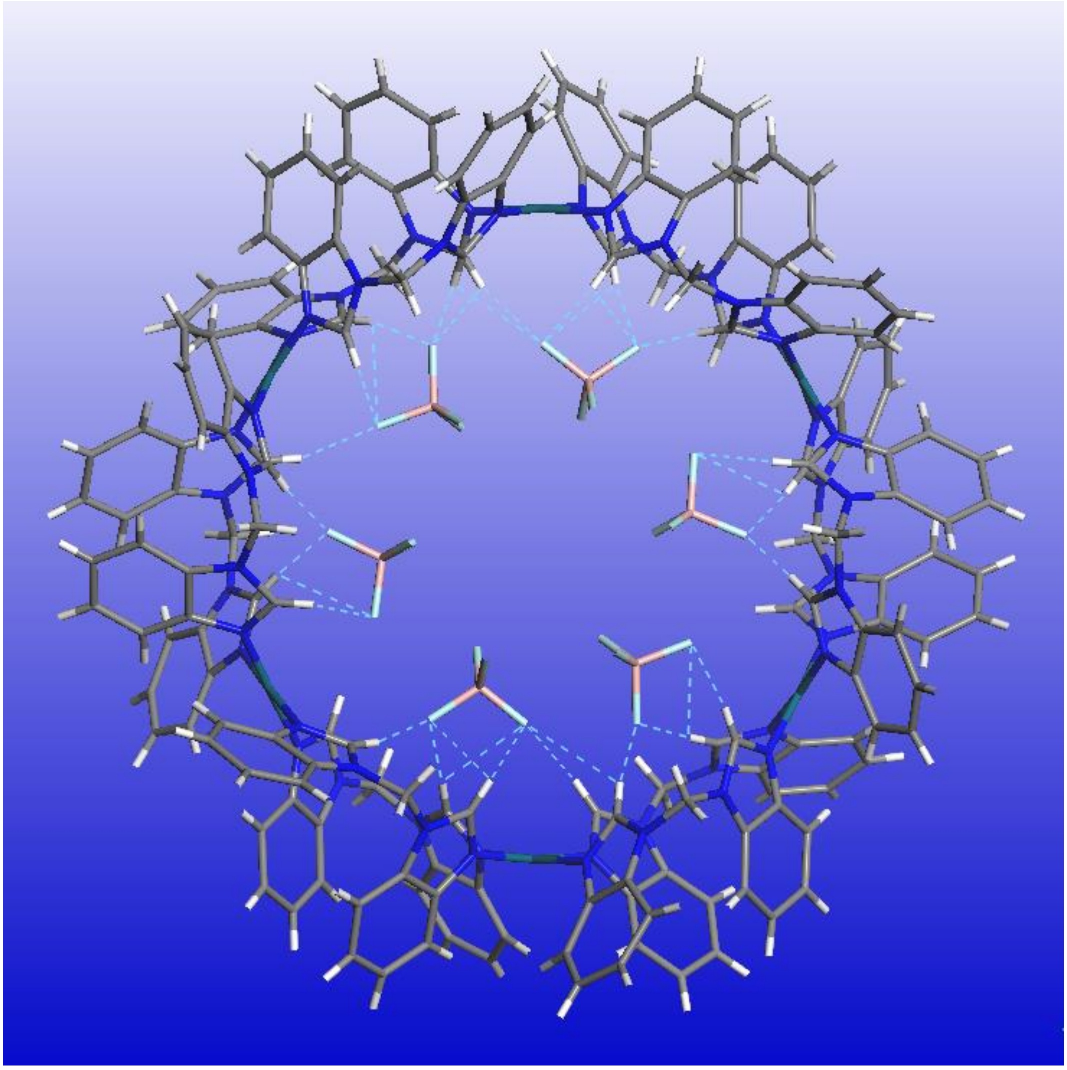

Supplementary Figure 41 One of the possible conformations for the six BF_4^- anions inside Pd_6L_{12} obtained from geometrical optimizations. (Atom coordinates from the crystal structure are fixed and only the conformations of BF_4^- are optimized. H-bonding interactions are highlighted with dashed lines)

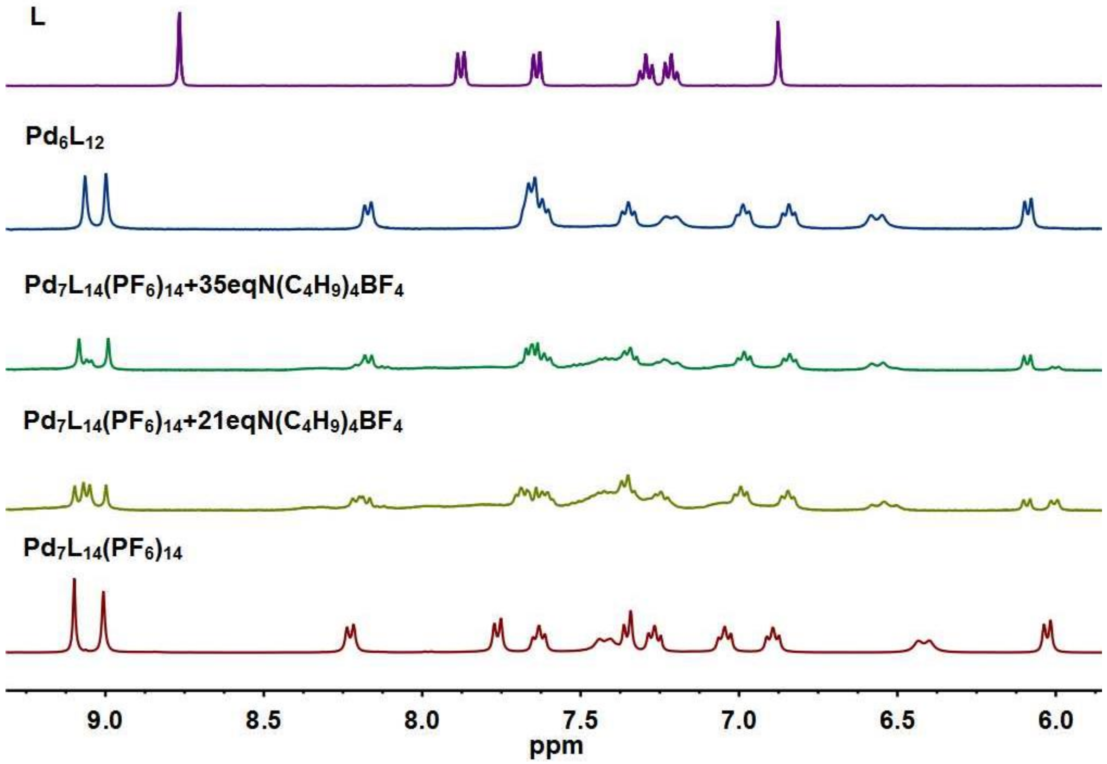

Supplementary Figure 42 ^{1}H NMR spectrum of complex $(Pd_7L_{14})(PF_6)_{14}$ after the addition of 21 eq. and 35 eq. of $N(C_4H_9)_4BF_4$, respectively (400 MHz, [D$_6$]DMSO, 298K)

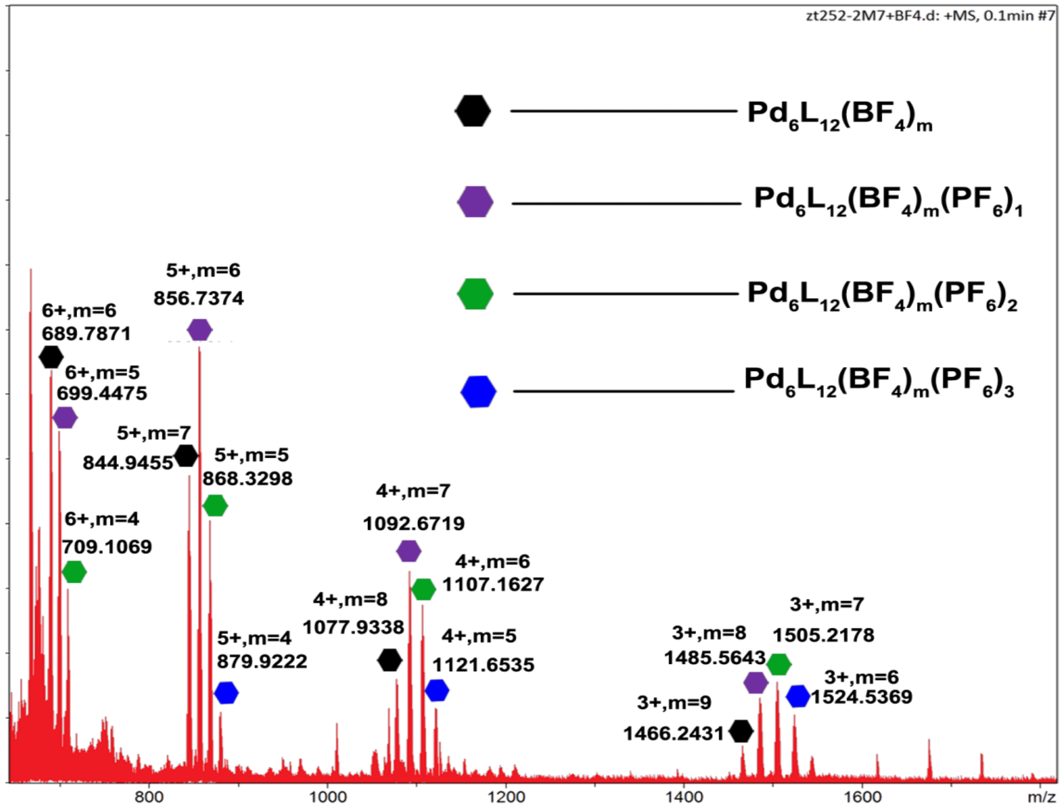

Supplementary Figure 43 ESI-Q-TOF mass spectrum for the mixed-anion complexes after the addition 35 eq. of $N(C_4H_9)_4BF_4$ into $(Pd_7L_{14})(PF_6)_{14}$

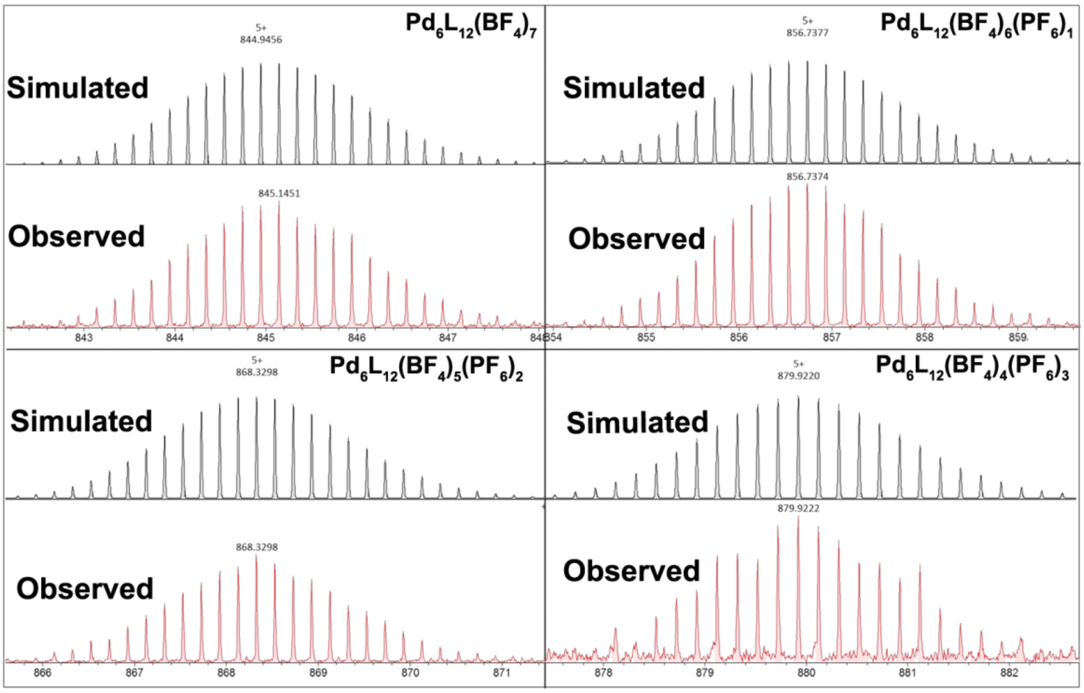

Supplementary Figure 44 The representative observed and simulated isotope patterns for the mixed-anion complexes shown in Supplementary Figure44

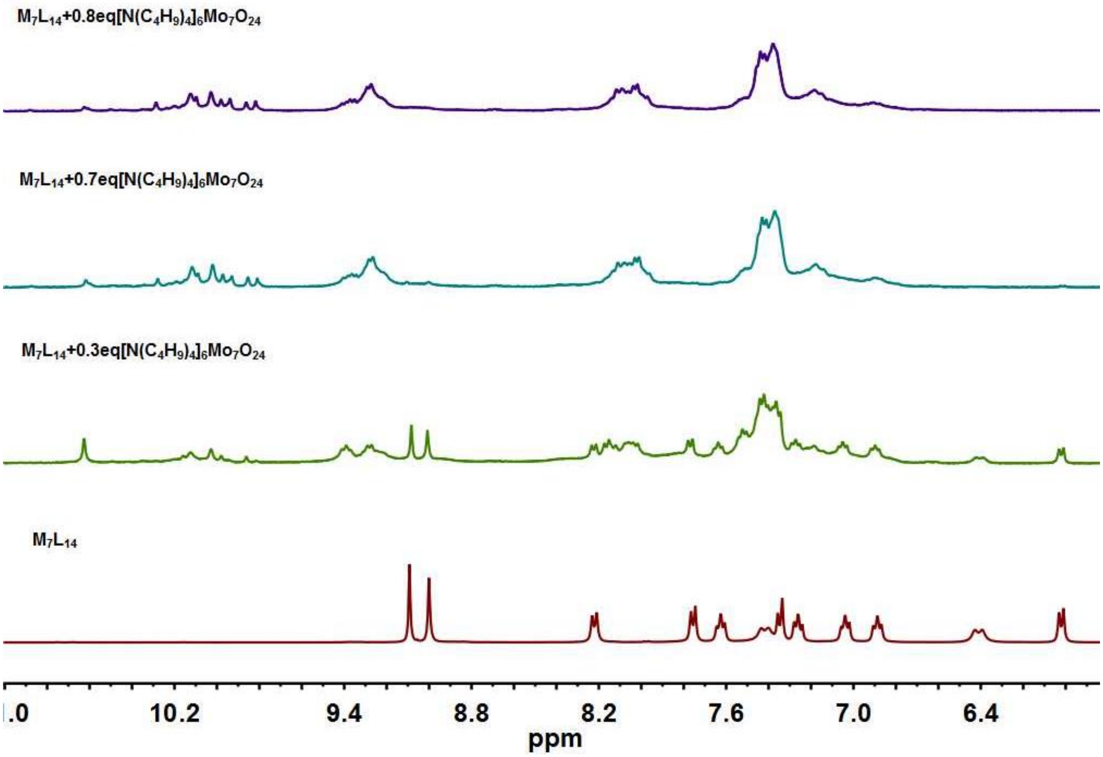

Supplementary Figure 45 ^{1}H NMR spectrum of complex $(Pd_7L_{14})(PF_6)_{14}$ after the addition of 0.3 ,0.7 eq. and 0.8 eq. of[$N(C_4H_9)_4]_6Mo_7O_{24}$, respectively (400 MHz, [D_6]DMSO, 298K)

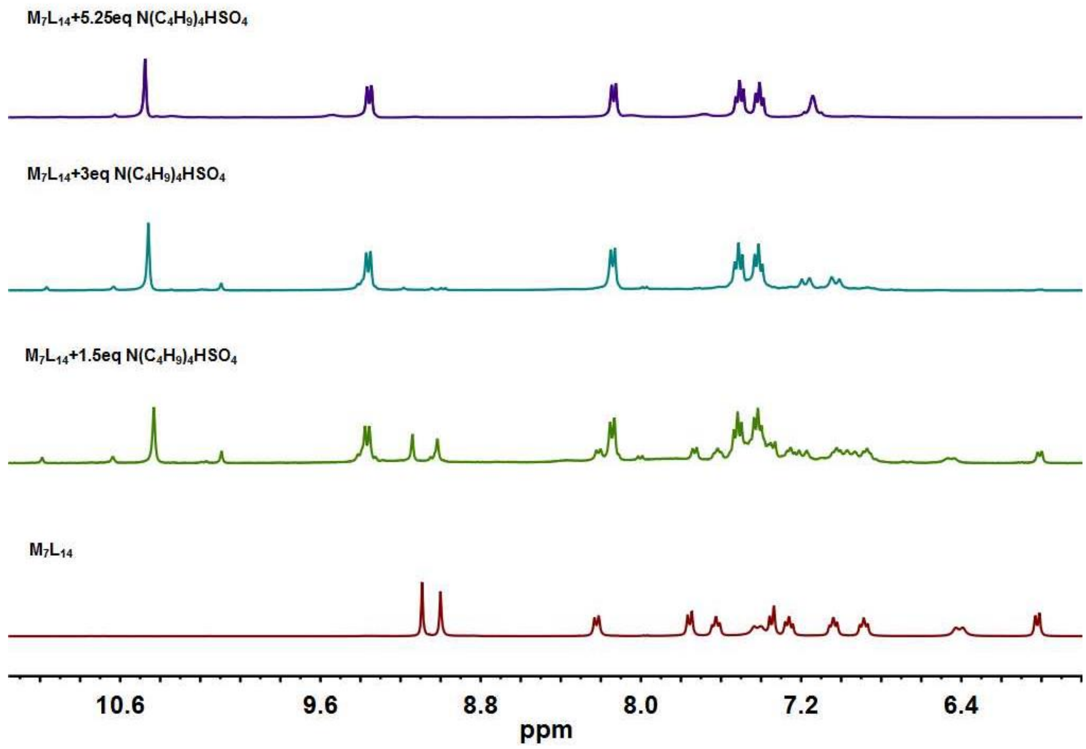

Supplementary Figure 46 ^{1}H NMR spectrum of complex $(Pd_7L_{14})(PF_6)_{14}$ after the addition of 1.5, 3 and 5.25 eq. of $N(C_4H_9)_4HSO_4$, respectively (400 MHz, $[D_6]$DMSO, 298K)

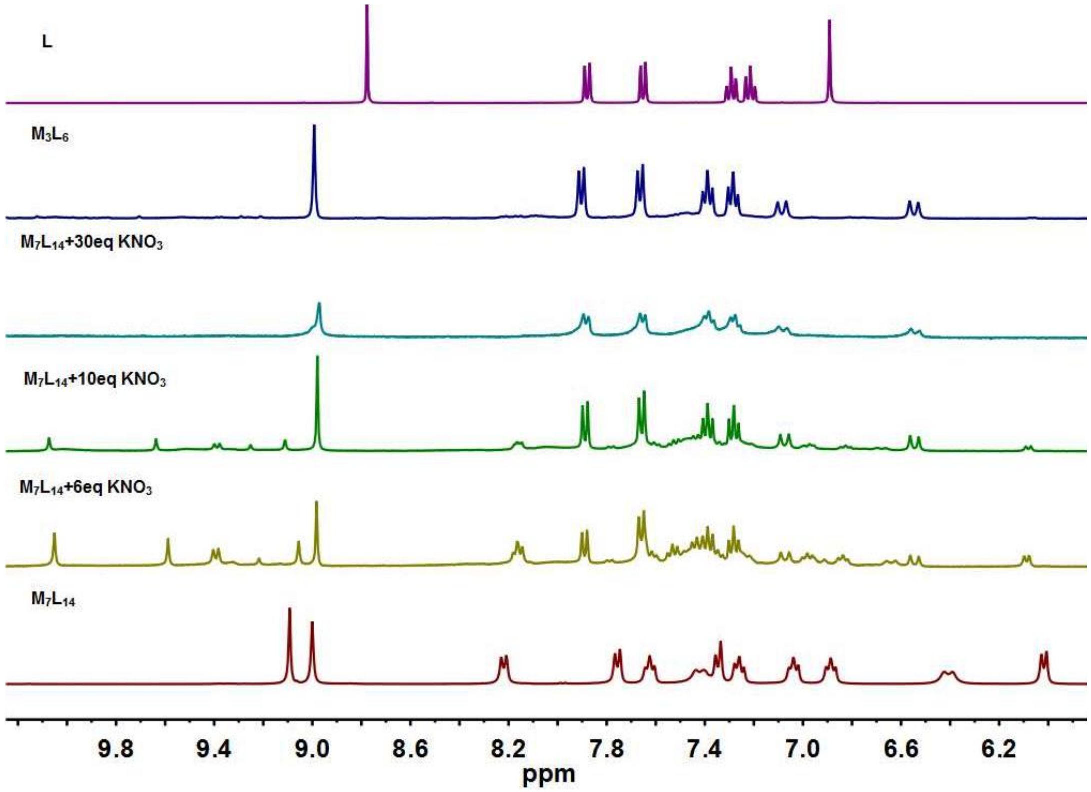

Supplementary Figure 47 ^{1}H NMR spectrum of complex $(Pd_7L_{14})(PF_6)_{14}$ after the addition of 5,10 and 30 eq. of KNO_3, respectively (400 MHz, $[D_6]$DMSO, 298K)

Display Report

Analysis Info		Acquisition D	2016/10/17 ĐÇÆÚÒ» ÏÂÎç 22:10:22
Analysis Name	C:\Users\Administrator\Desktop\project1-pictrue\M7+NO3\zt325-11_2-b,6_01_5667.d		
Method	pos-sqf-pd-500-3000_high.m	Operator	BDAL@DE
Sample Name	zt325-11	Instrumen	impact II 1825265.10133
Comment			

Acquisition Paramet

Source Type	ESI	Ion Polarity	Positive	Set Nebulizer	1.0 Bar
Focus	Active	Set Capillary	4500 V	Set Dry Heater	200 °C
Scan Begin	500 m/z	Set End Plate Offset	-500 V	Set Dry Gas	4.5 l/min
Scan End	3000 m/z	Set Charging Voltage	2000 V	Set Divert Valve	Waste
		Set Corona	0 nA	Set APCI Heater	0 °C

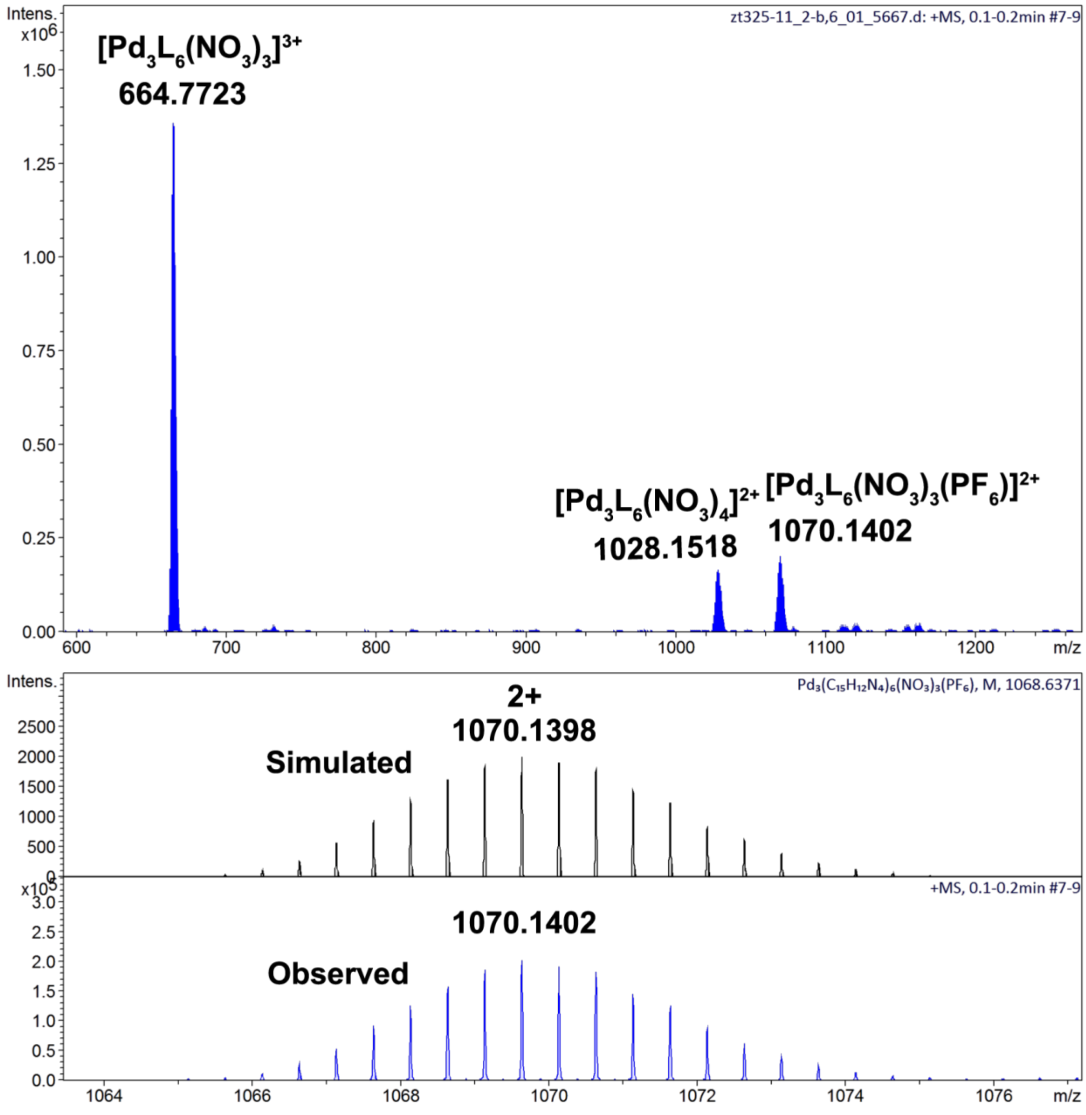

zt325-11_2-b,6_01_5667.d

Bruker Compass DataAnalysis 4.3 printe 2016/11/25 ĐÇÆÚÎå ÉÏÎç 0:45:13 Page 1 of 1

Supplementary Figure 48 ESI-Q-TOF mass spectrum of complex $(Pd_7L_{14})(PF_6)_{14}$ after the addition 30 eq. of KNO_3 and observed and calculated isotope patterns of 2+.

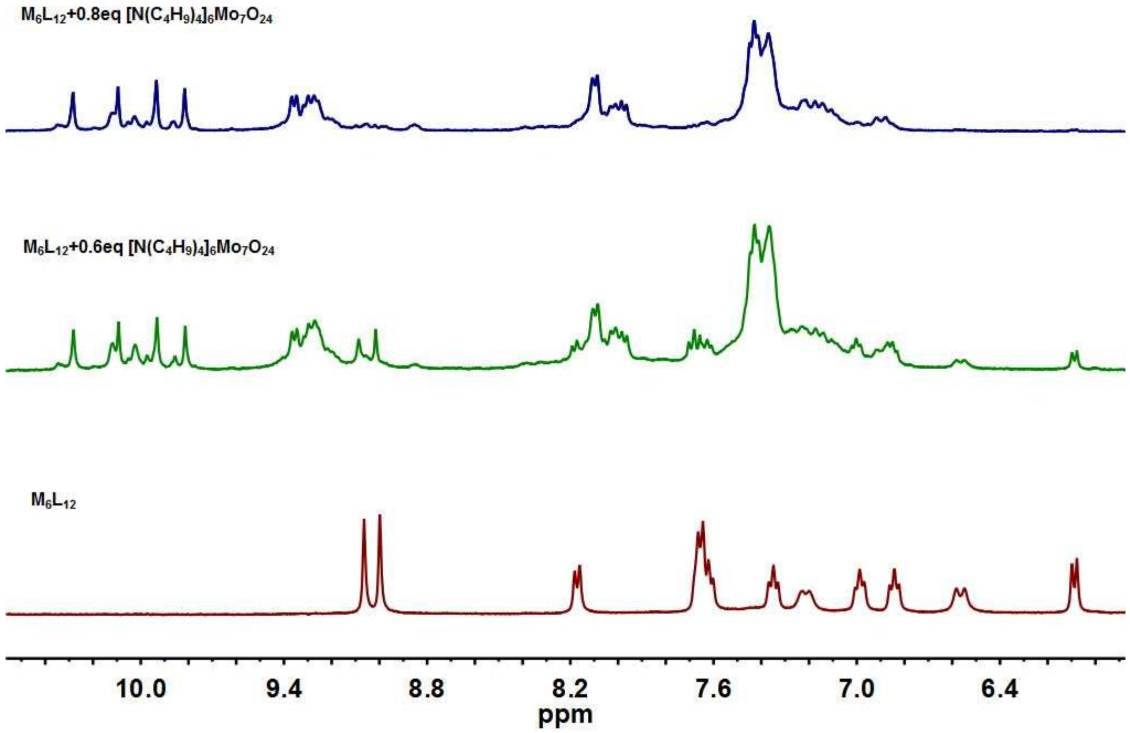

Supplementary Figure 49 ^{1}H NMR spectrum of complex $(Pd_6\mathbf{L}_{12})(BF_4)_{12}$ after the addition of 0.6 and 0.8 eq. of[$N(C_4H_9)_4]_6Mo_7O_{24}$, respectively (400 MHz, [D_6]DMSO, 298K).

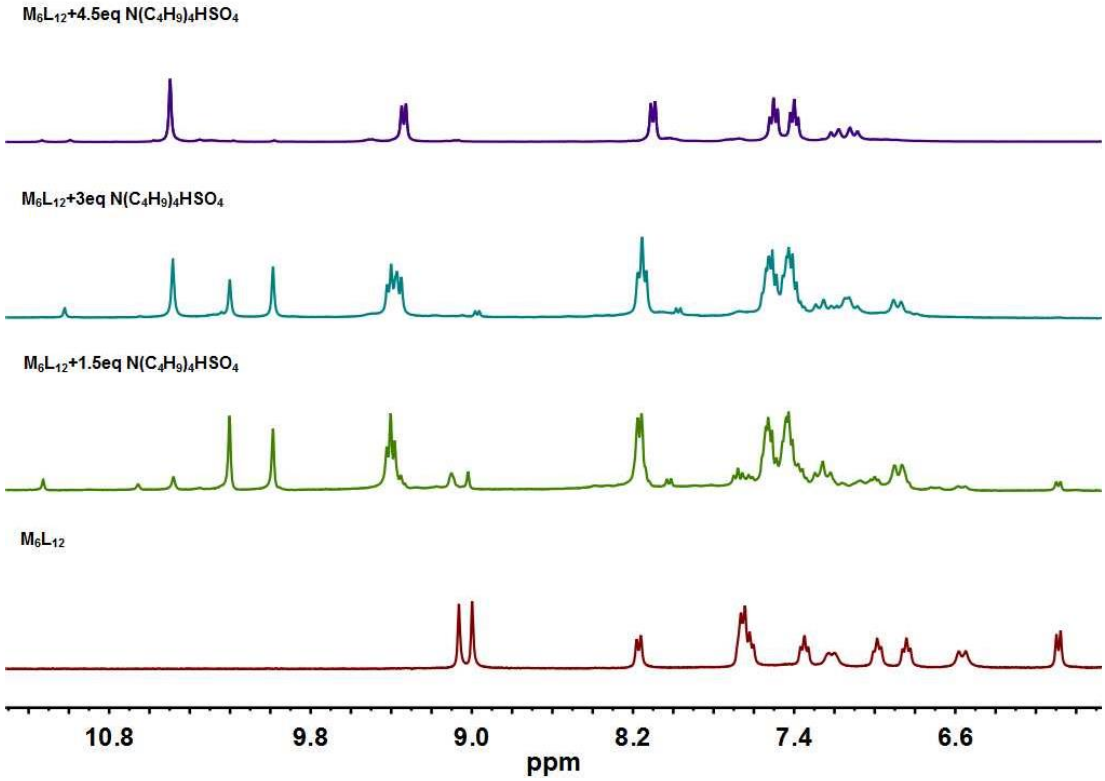

Supplementary Figure 50: ^{1}H NMR spectrum of complex $(Pd_6\mathbf{L}_{12})(BF_4)_{12}$ after the addition of 1.5, 3 and 4.5 eq. of $N(C_4H_9)_4HSO_4$, respectively (400 MHz, [D_6]DMSO, 298K)

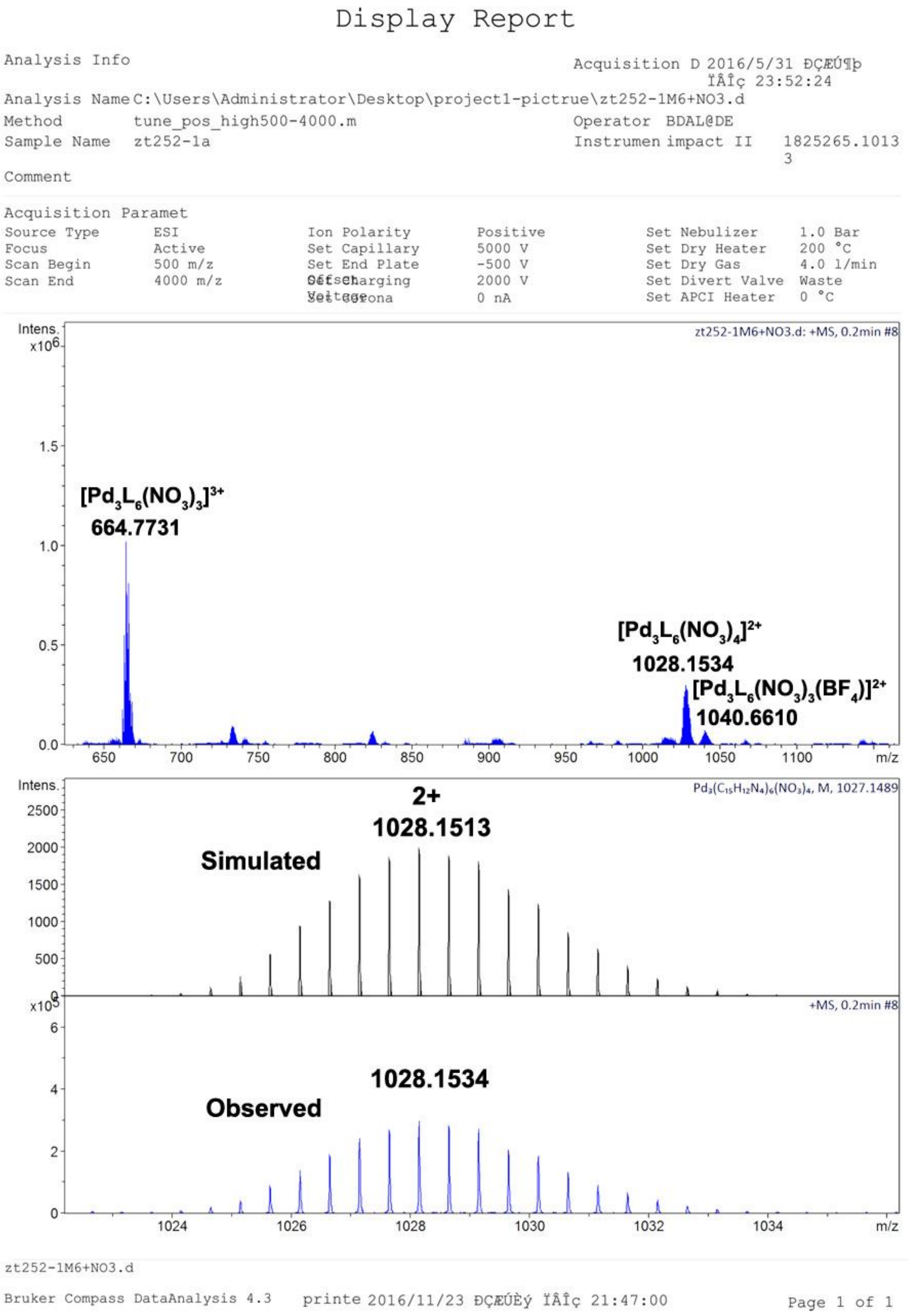

Supplementary Figure 51 ESI-Q-TOF mass spectrum of complex $(Pd_6L_{12})(BF_4)_{12}$ after the addition of 12 eq. of KNO_3 and observed and calculated isotope patterns of 2+

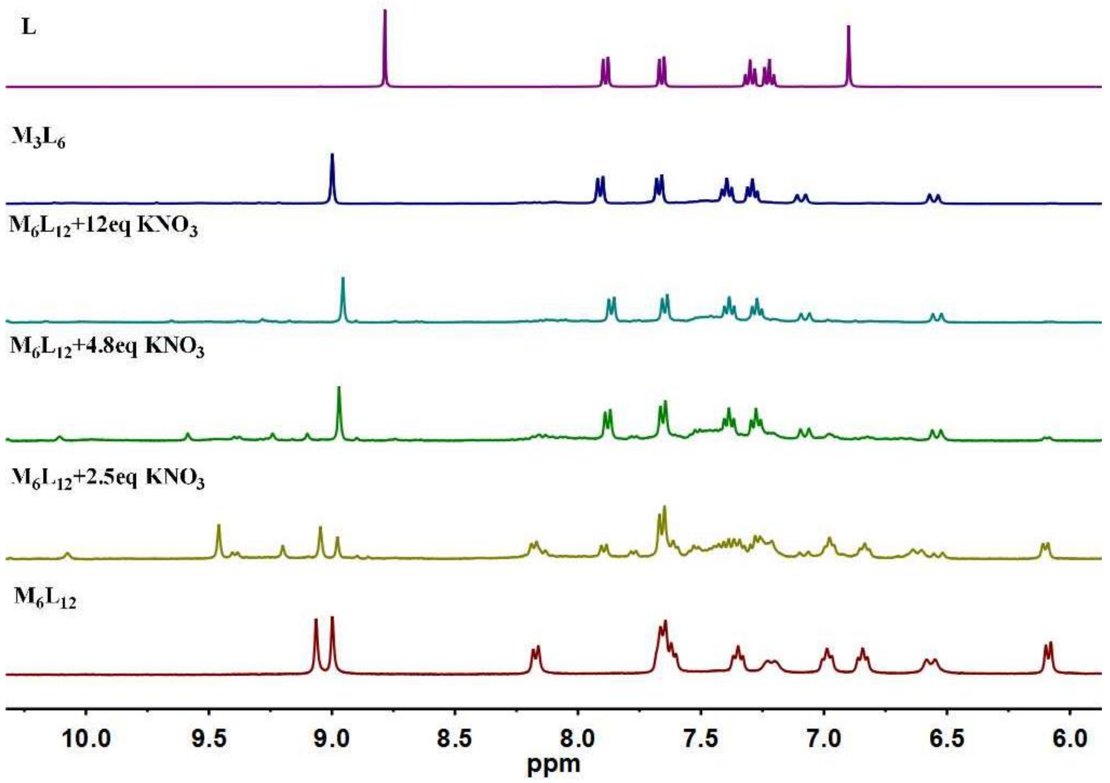

Supplementary Figure 52 ^{1}H NMR spectrum of complex $(Pd_6L_{12})(BF_4)_{12}$ after the addition of 2.5, 4.8 and 12 eq. of KNO_3, respectively (400 MHz, $[D_6]$DMSO, 298K).

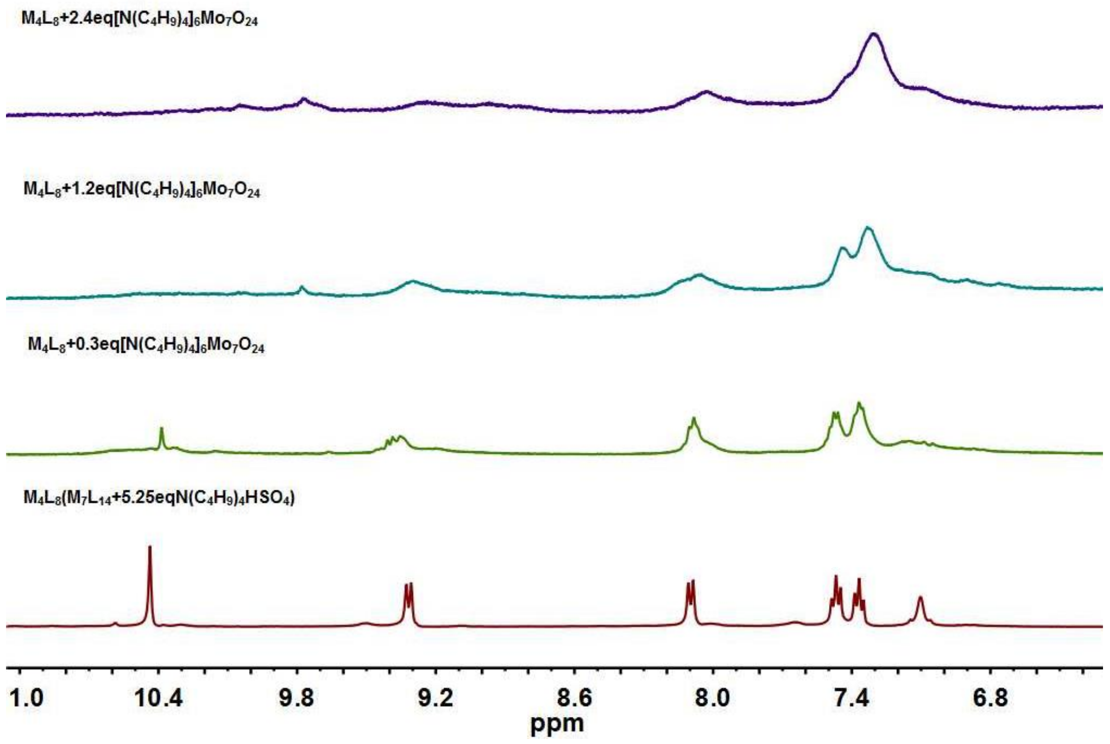

Supplementary Figure 53 ^{1}H NMR spectrum of complex $(Pd_4\mathbf{L}_8)(SO_4)_2(PF_6)_6$ after the addition of 0.3 , 1.2 and 2.4eq. of $[N(C_4H_9)_4]_6Mo_7O_{24}$, respectively (400 MHz, $[D_6]$DMSO, 298K).

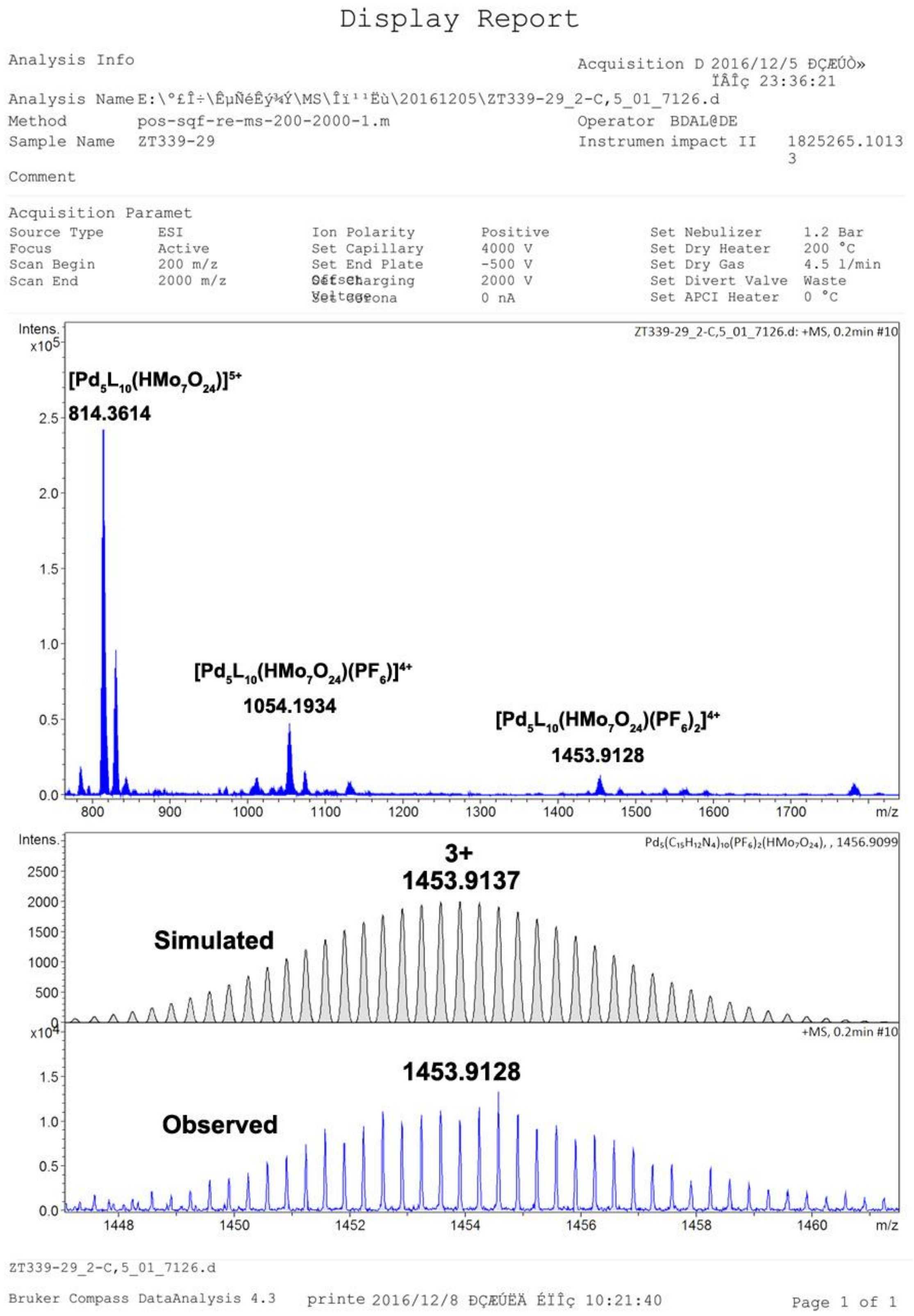

Supplementary Figure 54 ESI-Q-TOF mass spectrum of complex $Pd_4L_8(SO_4)_2(PF_6)_4$ after the addition of 2.4 eq. of $[N(C_4H_9)_4]_6Mo_7O_{24}$ and observed and calculated isotope patterns of 3+

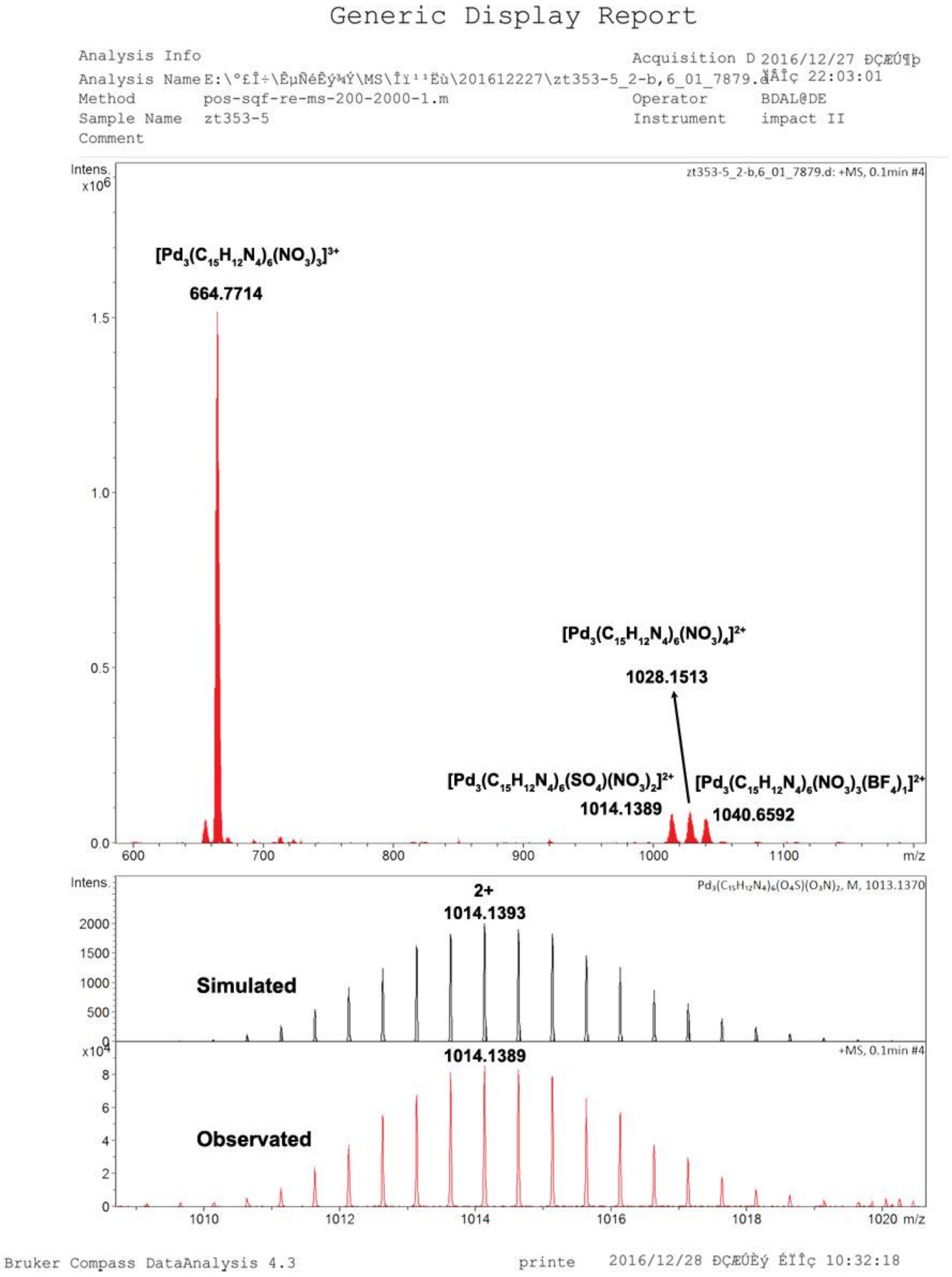

Supplementary Figure 55 ESI-Q-TOF mass spectrum of complex $Pd_4L_8(SO_4)_2(BF_4)_4$ after the addition of 13.3 eq. of KNO_3 and observed and calculated isotope patterns of 2+

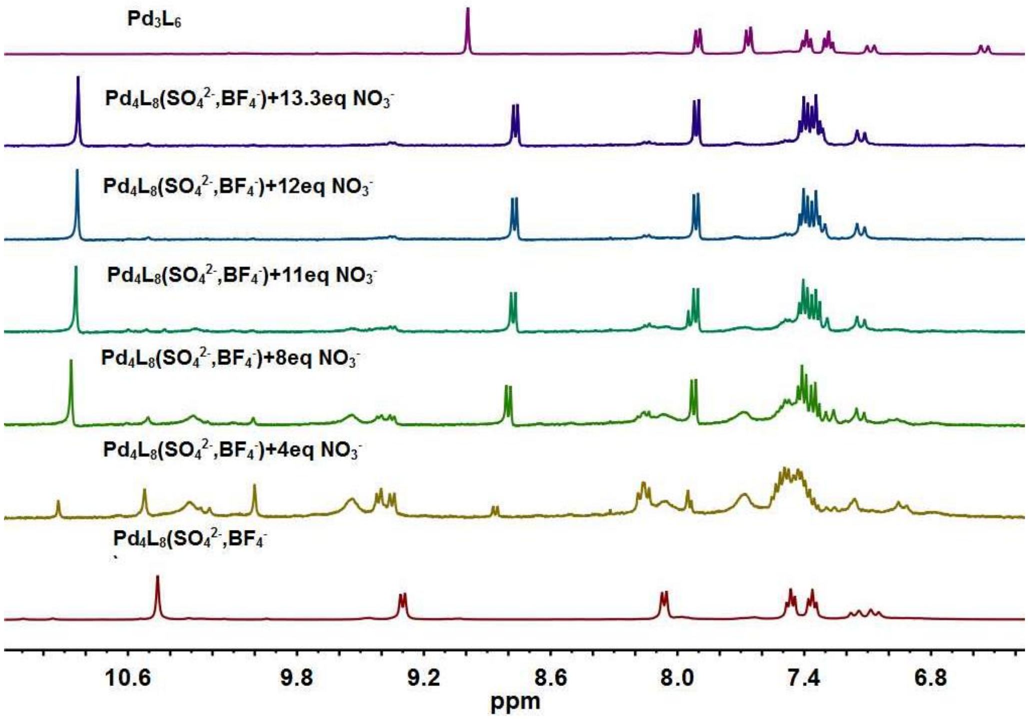

Supplementary Figure 56 ^{1}H NMR spectrum of complex $(Pd_4\mathbf{L}_8)(SO_4)_2(BF_4)_4$ after the addition of 4, 8, 11, 12 and 13.3 eq. of KNO_3, respectively (400 MHz, [D$_6$]DMSO, 298K).

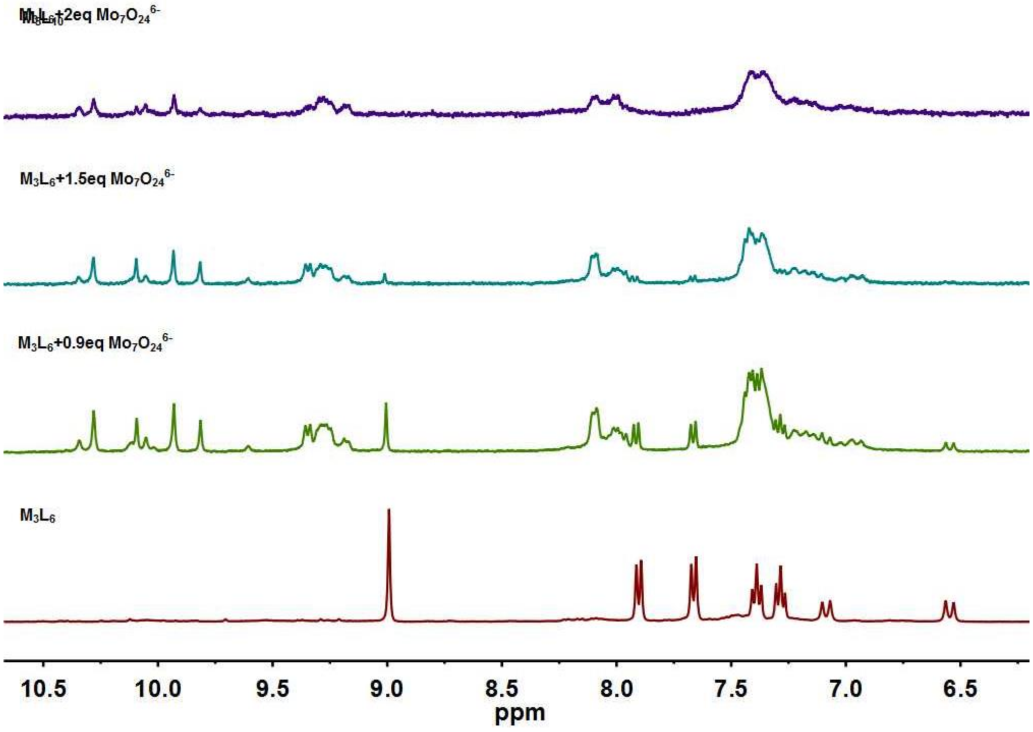

Supplementary Figure 57 ^{1}H NMR spectrum of complex $(Pd_3L_6)(NO_3)_6$ after the addition of 0.9, 1.5 and 2 eq. of $N(C_4H_9)_4Mo_7O_{24}$, respectively (400 MHz, [D$_6$]DMSO, 298K)

Display Report

Analysis Info | Acquisition D 2016/11/29 ĐÇÆÚ¶þ ÉÏÎç 0:02:14

Analysis Name	C:\Users\Administrator\Desktop\project1-pictrue\zt340-16M3-M5.d		
Method	pos-sqf-re-ms-200-2000-1.m	Operator	BDAL@DE
Sample Name	zt340-16	Instrumen	impact II 1825265.10133
Comment			

Acquisition Paramet

Source Type	ESI	Ion Polarity	Positive	Set Nebulizer	1.2 Bar
Focus	Active	Set Capillary	4000 V	Set Dry Heater	200 °C
Scan Begin	200 m/z	Set End Plate Offset	-500 V	Set Dry Gas	4.5 l/min
Scan End	2000 m/z	Set Charging Voltage	2000 V	Set Divert Valve	Waste
		Set Corona	0 nA	Set APCI Heater	0 °C

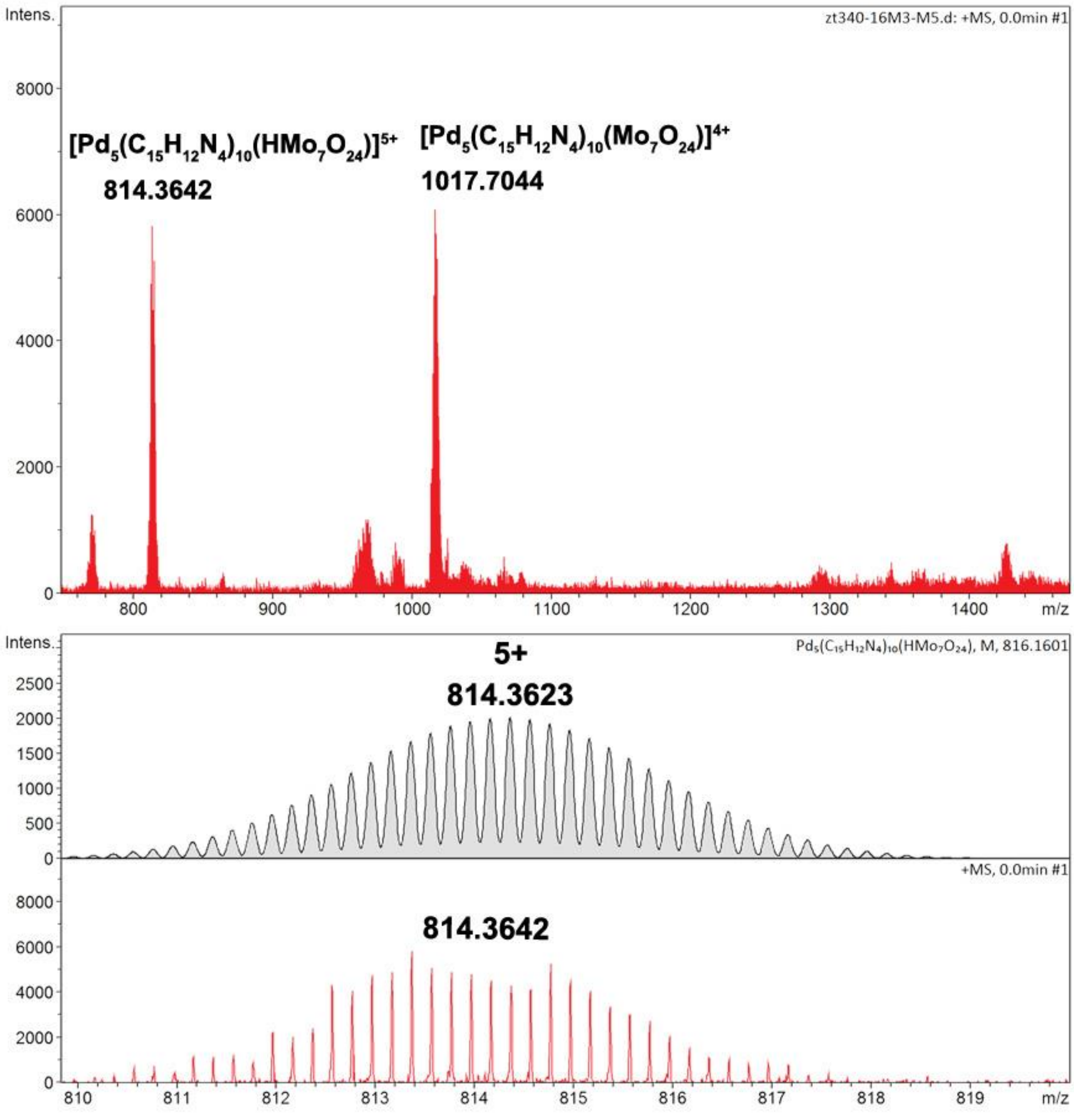

zt340-16M3-M5.d

Bruker Compass DataAnalysis 4.3 printe 2016/12/6 ĐÇÆÚ¶þ ÏÂÎç 17:01:39 Page 1 of 1

Supplementary Figure 58 ESI-Q-TOF mass spectrum of complex $(Pd_3L_6)(NO_3)_6$ after the addition of 2 eq. of $[N(C_4H_9)_4]_6Mo_7O_{24}$ and observed and calculated isotope patterns of 5+

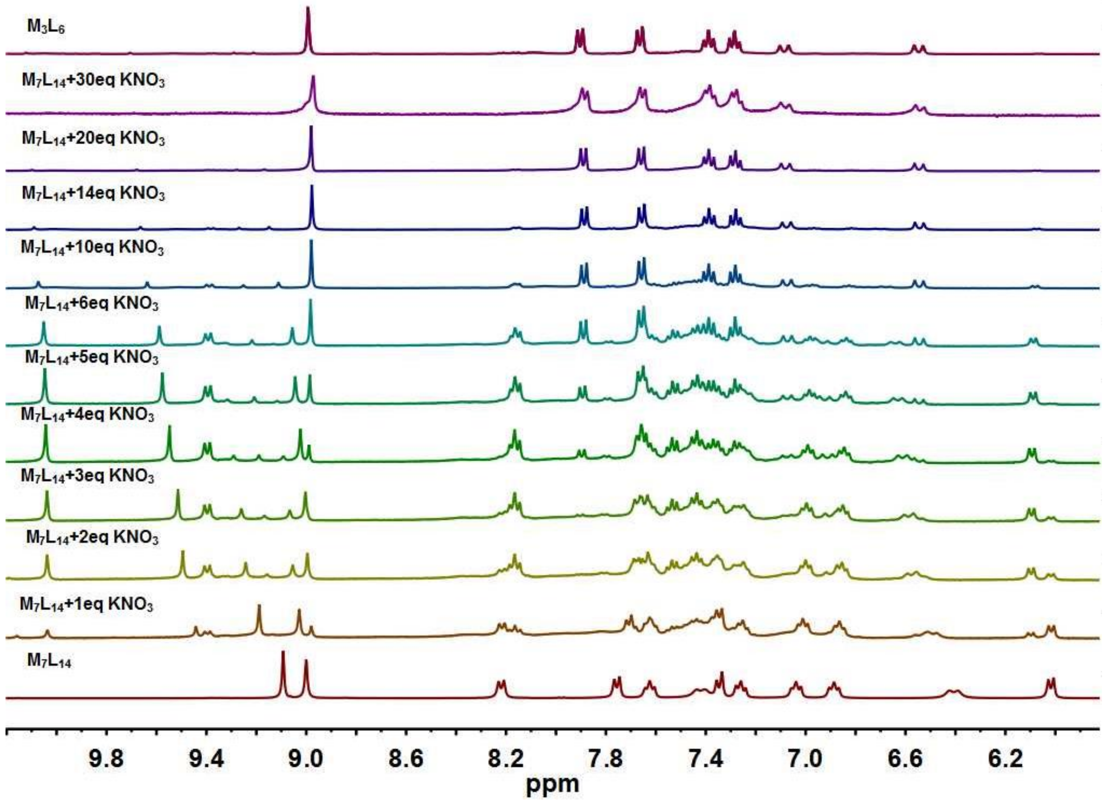

Supplementary Figure 59 ^{1}H NMR spectrum of complex $(Pd_7L_{14})(PF_6)_{14}$ after the addition of KNO_3(400 MHz, [D_6]DMSO, 298K)

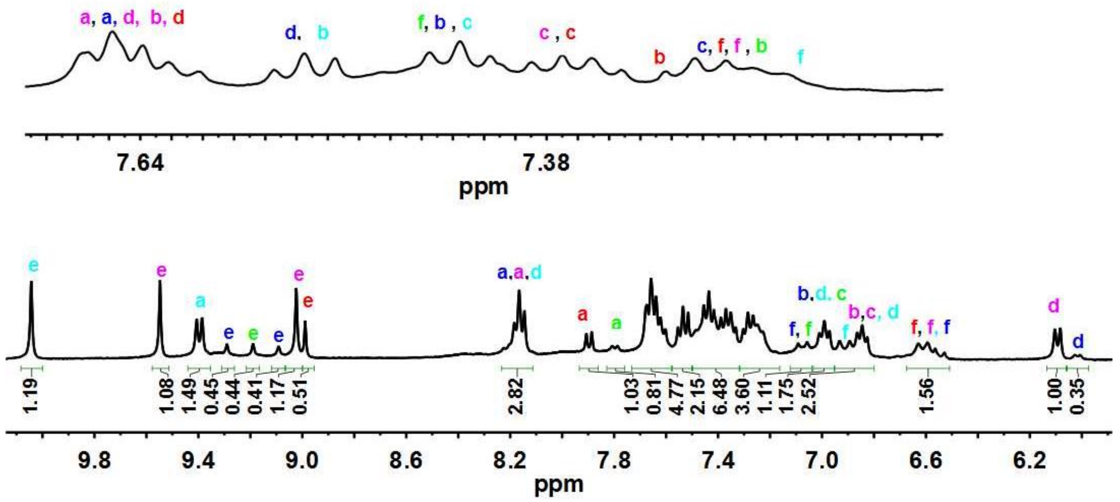

Supplementary Figure 60 ^{1}H NMR spectrum of complex $(Pd_7L_{14})(PF_6)_{14}$ after the addition of 4 eq. of KNO_3(Pd_3L_6 red, Pd_4L_8 sky blue, Pd_5L10_6 green, Pd_6L_{12} pink and Pd_7L_{14} dark blue)(400MHz, [D_6]DMSO, 298K)

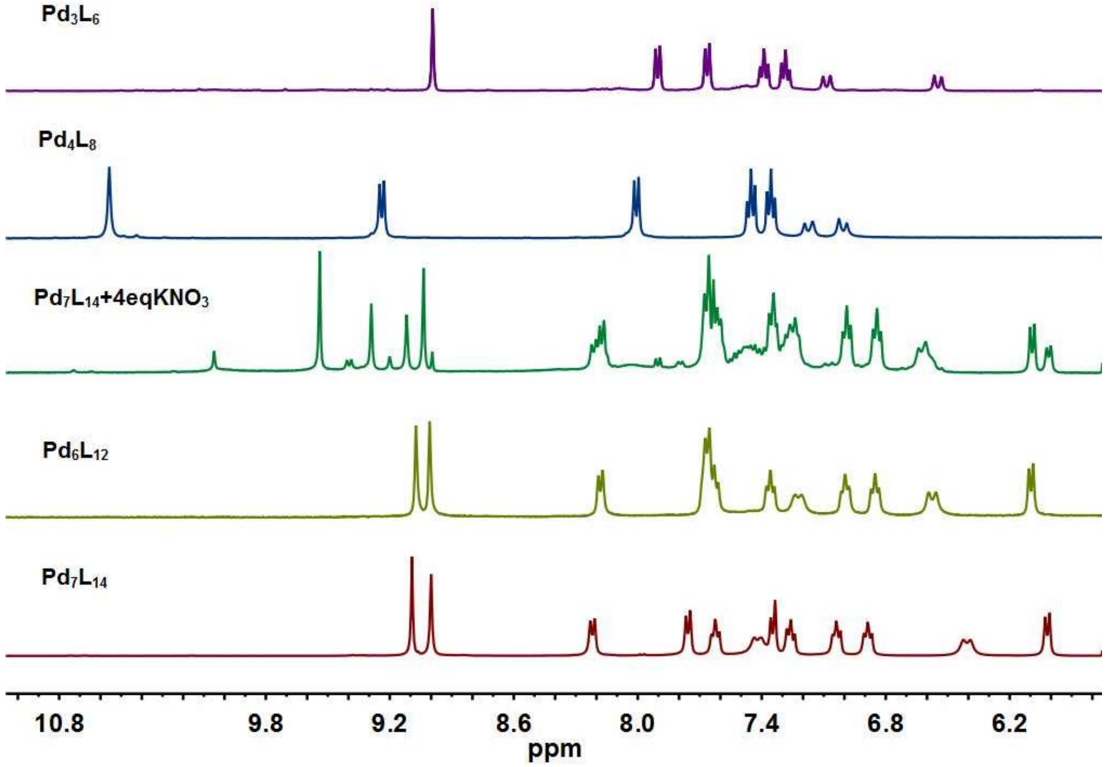

Supplementary Figure 61 ^{1}H NMR spectrum of complex Pd_3L_6, Pd_4L_8, Pd_6L_{12}, Pd_7L_{14} and $(Pd_7L_{14})(PF_6)_{14}$ with 4 eq. of KNO_3(400 MHz, [D6]DMSO, 298K)

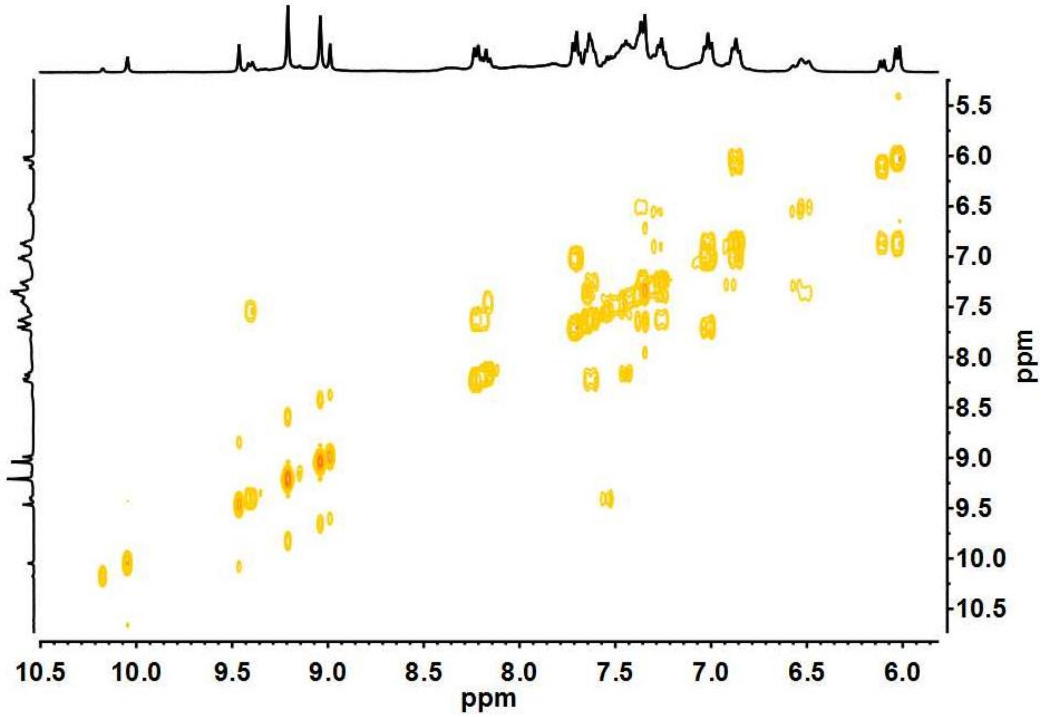

Supplementary Figure 62 ^{1}H-^{1}H COSY NMR spectrum of the $(Pd_7L_{14})(PF_6)_{14}$ after the addition of 1 eq. of KNO_3 (400 MHz, [D6]DMSO, 298K)

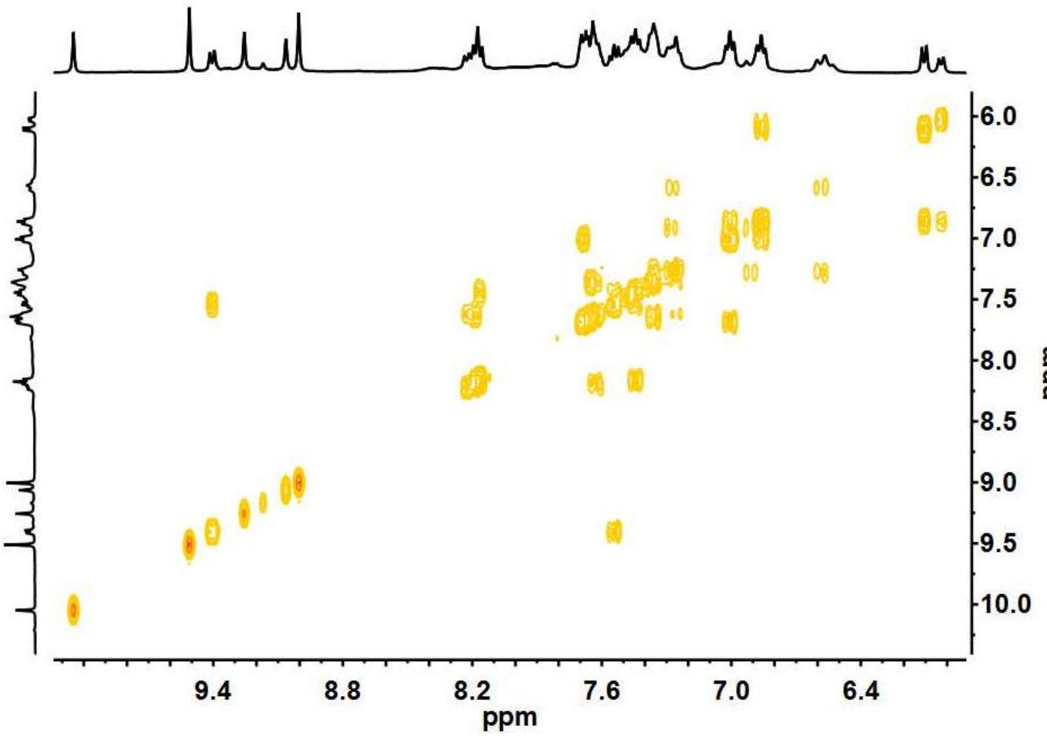

Supplementary Figure 63 ^1H-^1H COSY NMR spectrum of the $(Pd_7L_{14})(PF_6)_{14}$ after the addition of 2 eq. of KNO_3. (400 MHz, [D$_6$]DMSO, 298K)

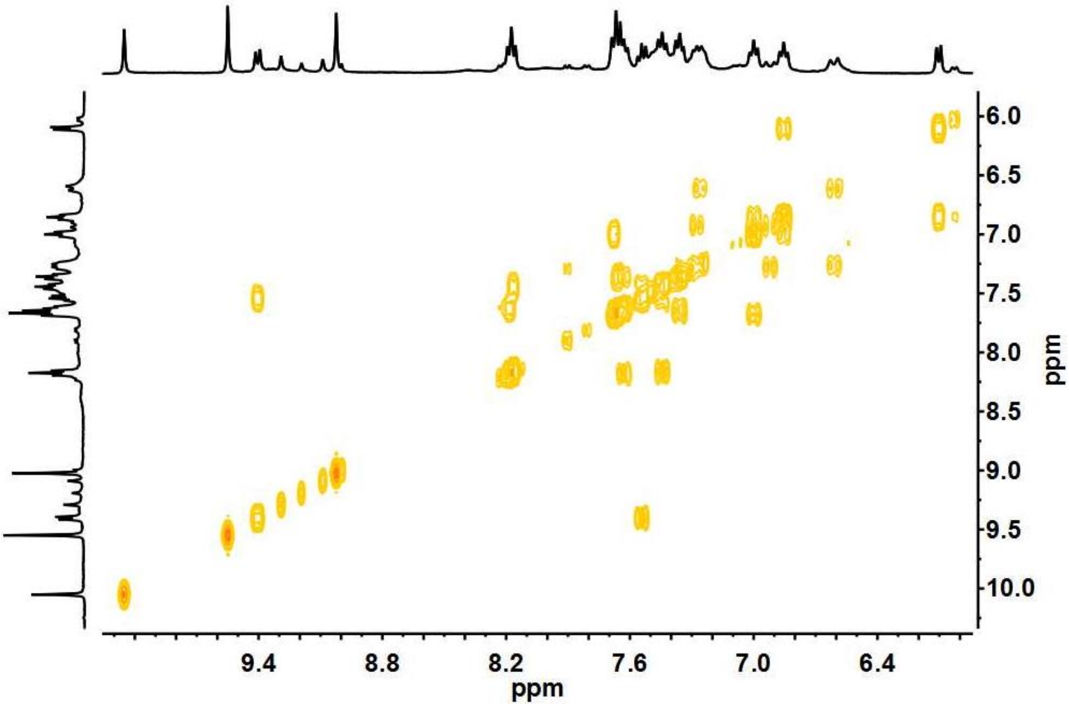

Supplementary Figure 64 ^1H-^1H COSY NMR spectrum of the $(Pd_7L_{14})(PF_6)_{14}$ after the addition of 3 eq. of KNO_3 .(400 MHz, [D$_6$]DMSO, 298K)

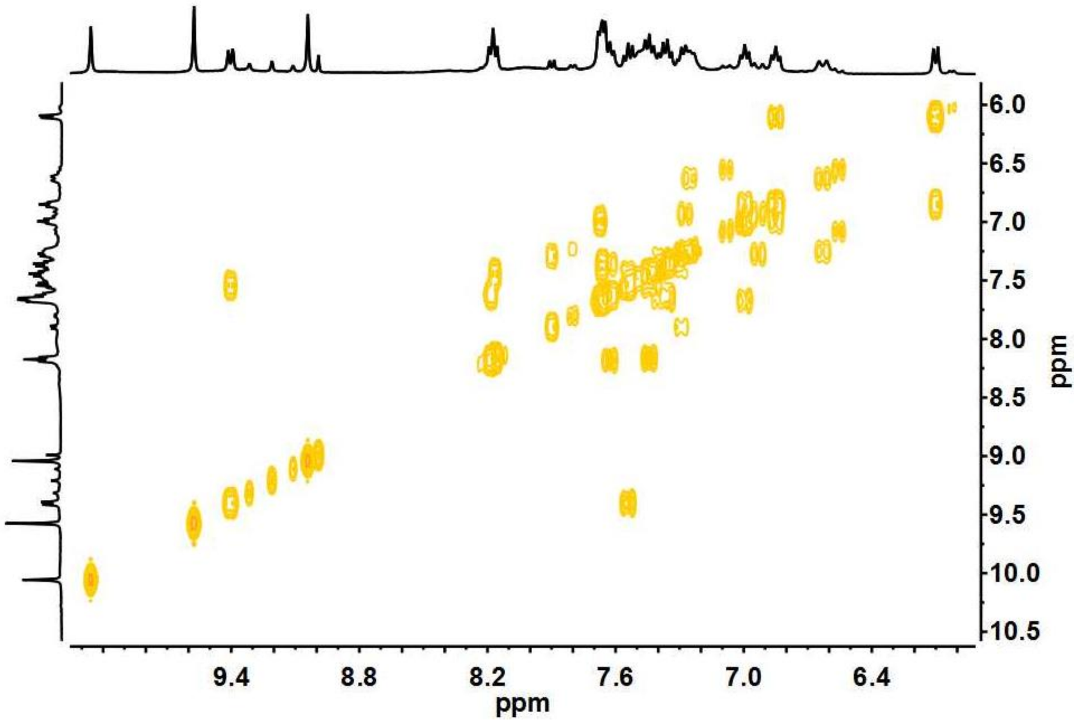

Supplementary Figure 65 ^1H-^1H COSY NMR spectrum of the $(Pd_7L_{14})(PF_6)_{14}$ after the addition of 4 eq. of KNO_3 .(400 MHz, [D$_6$]DMSO, 298K)

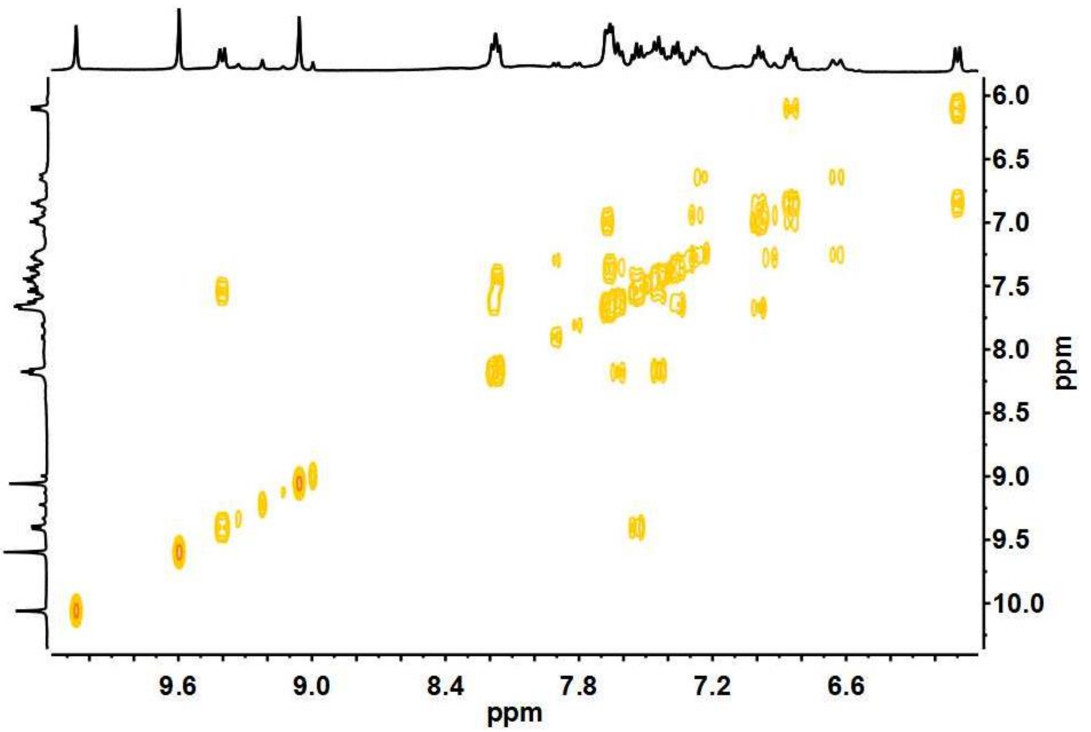

Supplementary Figure 66 ^1H-^1H COSY NMR spectrum of the $(Pd_7L_{14})(PF_6)_{14}$ after the addition of 5 eq. of KNO_3 .(400 MHz, [D$_6$]DMSO, 298K)

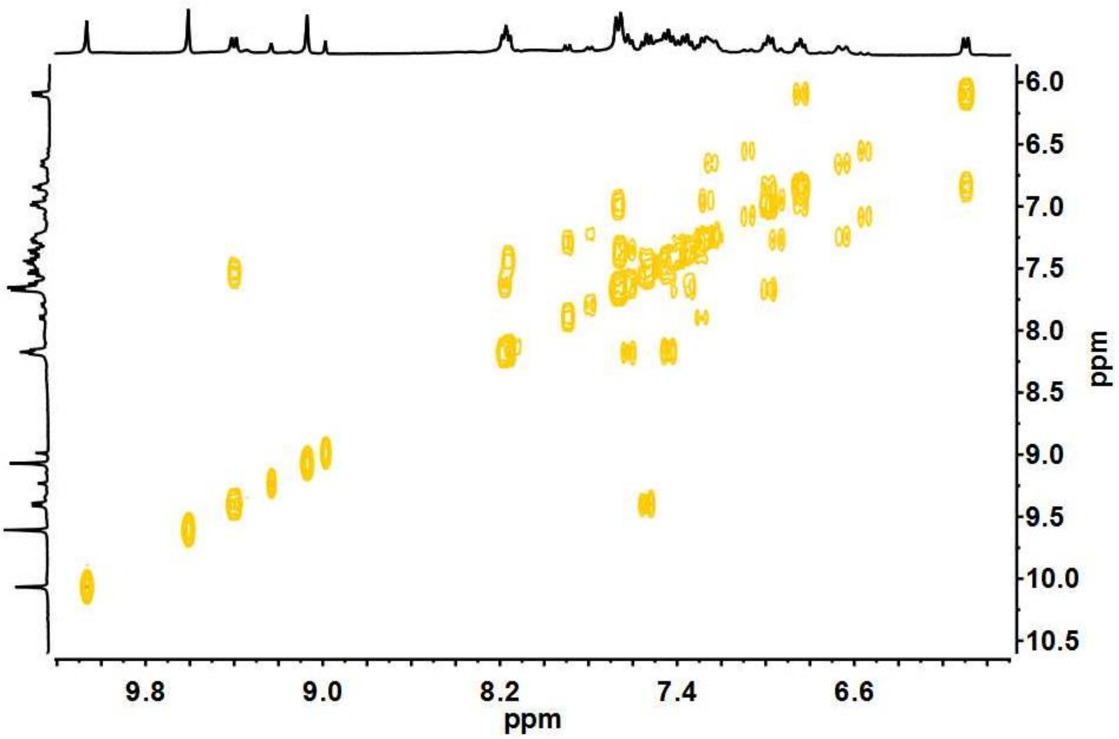

Supplementary Figure 67 ^{1}H-^{1}H COSY NMR spectrum of the $(Pd_7L_{14})(PF_6)_{14}$ after the addition of 6 eq. of KNO_3 .(400 MHz, [D$_6$]DMSO, 298K)

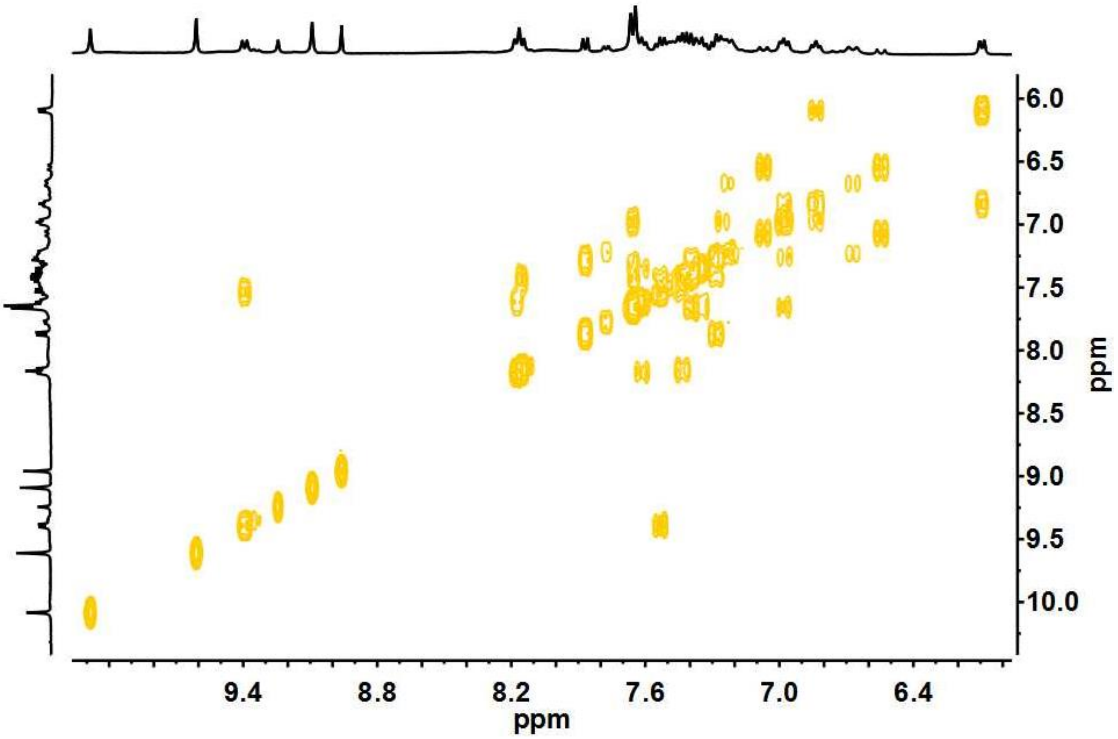

Supplementary Figure 68 ^{1}H-^{1}H COSY NMR spectrum of the $(Pd_7L_{14})(PF_6)_{14}$ after the addition of 10 eq. of KNO_3 .(400 MHz, [D$_6$]DMSO, 298K)

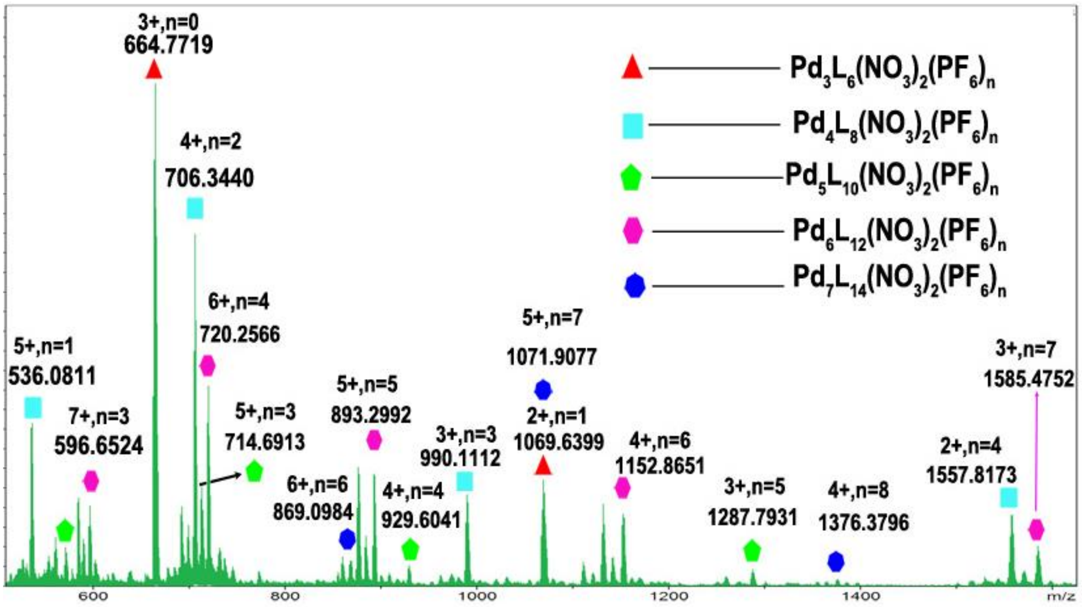

Supplementary Figure 69 ESI-Q-TOF mass spectrum of intermediate complexes after after the addition 4 eq. of KNO_3 to $(Pd_7L_{14})(PF_6)_{14}$.

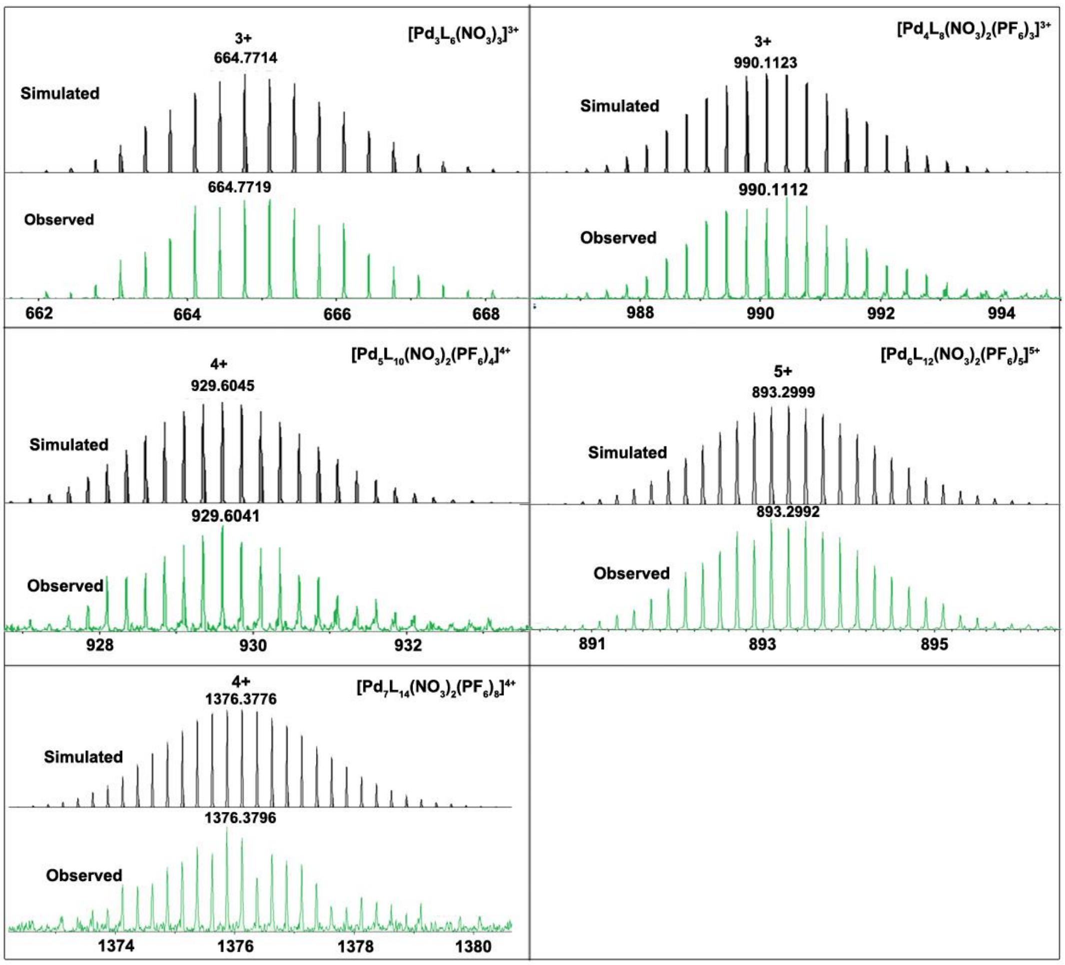

Supplementary Figure 70 The representative observed and simulated isotope patterns shown in Supplementary Figure 70

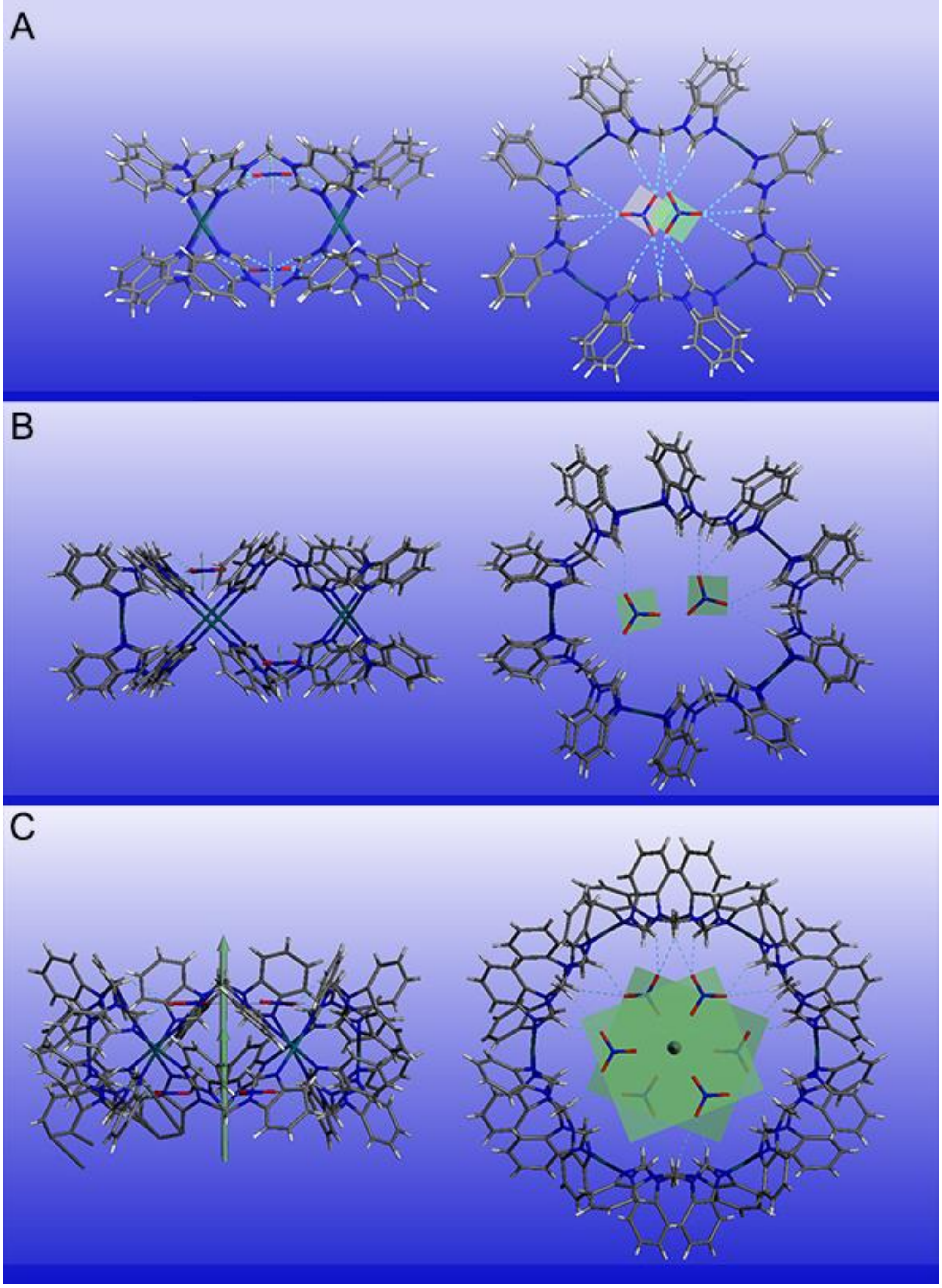

Supplementary Figure 71 Snapshots for the possible geometries of (A) $(NO_3)_2@Pd_4L_8$, (B) $(NO_3)_2@Pd_5L_{10}$ and (C) $(NO_3)_6@Pd_6L_{12}$ obtained by molecular modeling. Geometries for the Pd_nL_{2n} macrocycles and the NO_3 anion are adopted from the crystal structures. It can be seen that 1): Both Pd_4L_8 and Pd_5L_{10} can accommodate up to two NO_3 anions, but NO_3 anions are involved in more hydrogen bonding interactions (highlighted by light-green dashed lines) with Pd_4L_8 compared to Pd_5L_{10}; 2): Pd_6L_{12} can accommodate up to six NO_3 anions. We need to point out that these results are for very rough estimations only, because the contributions of the weakly hydrogen-bonding PF_6 anions and solvents molecules have been totally neglected during the modeling.

Supplementary Table 1 Crystal data and structure refinement for Pd_3L_6 (**2**)

Identification code	p-1	
Empirical formula	C97 H86 Cl14 N30 O18 Pd3	
Formula weight	2775.45	
Temperature	80(2) K	
Wavelength	0.68879 Å	
Crystal system	Triclinic	
Space group	P-1	
Unit cell dimensions	a = 18.088(4) Å	α= 73.70(3)°.
	b = 18.299(4) Å	β= 73.16(3)°.
	c = 20.871(4) Å	γ = 70.43(3)°.
Volume	6099(3) Å^3	
Z	2	
Density (calculated)	1.511 Mg/m^3	
Absorption coefficient	0.740 mm^{-1}	
F(000)	2796	
Crystal size	0.080 x 0.020 x 0.020 mm^3	
Theta range for data collection	1.009 to 25.499°.	
Index ranges	-22<=h<=22, -22<=k<=22, -26<=l<=26	
Reflections collected	46884	
Independent reflections	23481 [R(int) = 0.0273]	
Completeness to theta = 24.411°	94.5 %	
Absorption correction	None	
Refinement method	Full-matrix least-squares on F^2	
Data / restraints / parameters	23481 / 12 / 1460	
Goodness-of-fit on F^2	1.076	
Final R indices [I>2sigma(I)]	R1 = 0.1120, wR2 = 0.3624	
R indices (all data)	R1 = 0.1177, wR2 = 0.3659	
Extinction coefficient	n/a	
Largest diff. peak and hole	4.214 and -1.744 e.Å^{-3}	

Supplementary Table 2 Crystal data and structure refinement for Pd_4L_8 (SO_4^{2-} salt) (**3**)

Identification code	p21c_sq	
Empirical formula	C128 H122 N32 O28 Pd4 S10	
Formula weight	3302.77	
Temperature	293(2) K	
Wavelength	0.68879 Å	
Crystal system	Monoclinic	
Space group	$P2_1/c$	
Unit cell dimensions	a = 18.178(4) Å	α= 90°.
	b = 32.042(6) Å	β= 101.23(3)°.
	c = 18.582(4) Å	γ = 90°.
Volume	10616(4) $Å^3$	
Z	2	
Density (calculated)	1.033 Mg/m^3	
Absorption coefficient	0.442 mm^{-1}	
F(000)	3364	
Crystal size	0.25 x 0.22 x 0.20 mm^3	
Theta range for data collection	1.107 to 28.148°.	
Index ranges	-24<=h<=24, -43<=k<=43,	
	-25<=l<=25	
Reflections collected	93644	
Independent reflections	26904 [R(int) = 0.0809]	
Completeness to theta = 24.411°	99.8 %	
Refinement method	Full-matrix least-squares on F^2	
Data / restraints / parameters	26904 / 814 / 984	
Goodness-of-fit on F^2	1.042	
Final R indices [I>2sigma(I)]	R1 = 0.0765, wR2 = 0.2429	
R indices (all data)	R1 = 0.1161, wR2 = 0.2780	
Extinction coefficient	n/a	
Largest diff. peak and hole	1.421 and -1.269 e.$Å^{-3}$	

Supplementary Table 3 Crystal data and structure refinement for Pd_5L_{10} (**4**)

Identification code	pmmn_sq_sq	
Empirical formula	C150 H120 N40 Pd5	
Formula weight	3014.86	
Temperature	293(2) K	
Wavelength	0.68878 Å	
Crystal system	Orthorhombic	
Space group	Pmmn	
Unit cell dimensions	a = 20.3994(4) Å	α= 90°.
	b = 21.2991(4) Å	β= 90°.
	c = 29.5319(6) Å	γ = 90°.
Volume	12831.3(4) $Å^3$	
Z	2	
Density (calculated)	0.780 Mg/m^3	
Absorption coefficient	0.345 mm^{-1}	
F(000)	3060	
Crystal size	0.18 x 0.18 x 0.15 mm^3	
Theta range for data collection	0.668 to 25.498°.	
Index ranges	-25<=h<=25, -26<=k<=25, -36<=l<=36	
Reflections collected	188179	
Independent reflections	13683 [R(int) = 0.0591]	
Completeness to theta = 24.410°	98.6 %	
Absorption correction	None	
Refinement method	Full-matrix least-squares on F^2	
Data / restraints / parameters	13683 / 621 / 446	
Goodness-of-fit on F^2	2.991	
Final R indices [I>2sigma(I)]	R1 = 0.1540, wR2 = 0.5192	
R indices (all data)	R1 = 0.1627, wR2 = 0.5449	
Extinction coefficient	n/a	
Largest diff. peak and hole	5.923 and -2.097 e.$Å^{-3}$	

Supplementary Table 4 Crystal data and structure refinement for Pd_6L_{12} (**5**)

Identification code	r-3c_sq	
Empirical formula	C180 H144 F6 N48 Pd6	
Formula weight	3731.82	
Temperature	293(2) K	
Wavelength	0.68878 Å	
Crystal system	Trigonal	
Space group	R-3c	
Unit cell dimensions	a = 71.246(4) Å	α= 90°.
	b = 71.246(4) Å	β= 90°.
	c = 27.0831(16) Å	γ = 120°.
Volume	119056(15) $Å^3$	
Z	18	
Density (calculated)	0.937 Mg/m^3	
Absorption coefficient	0.406 mm^{-1}	
F(000)	34020	
Crystal size	0.21 x 0.21 x 0.21 mm^3	
Theta range for data collection	1.117 to 18.245°.	
Index ranges	-64<=h<=64, -64<=k<=64, -24<=l<=24	
Reflections collected	96189	
Independent reflections	10282 [R(int) = 0.1453]	
Completeness to theta = 18.245°	98.5 %	
Absorption correction	None	
Refinement method	Full-matrix least-squares on F^2	
Data / restraints / parameters	10282 / 2955 / 1066	
Goodness-of-fit on F^2	2.044	
Final R indices [I>2sigma(I)]	R1 = 0.1369, wR2 = 0.3885	
R indices (all data)	R1 = 0.1756, wR2 = 0.4507	
Extinction coefficient	n/a	
Largest diff. peak and hole	1.321 and -1.053 e.$Å^{-3}$	

Supplementary Table 5 The information of complex $(Pd_7L_{14})(PF_6)_{14}$ after the addition 35 eq. of $N(C_4H_9)_4BF_4$ in ESI-Q-TOF mass spectrum.

Formula	Charge	m/z(Simulated)	m/z(Observed)
$Pd_6(C_{15}H_{12}N_4)_{12}(BF_4)_6$	+6	689.7873	689.7871
$Pd_6(C_{15}H_{12}N_4)_{12}(BF_4)_7$	+5	844.9456	844.9455
$Pd_6(C_{15}H_{12}N_4)_{12}(BF_4)_8$	+4	1077.9331	1077.9338
$Pd_6(C_{15}H_{12}N_4)_{12}(BF_4)_9$	+3	1466.2455	1466.2431
$Pd_6(C_{15}H_{12}N_4)_{12}(BF_4)_5(PF_6)_1$	+6	699.4474	699.4475
$Pd_6(C_{15}H_{12}N_4)_{12}(BF_4)_6(PF_6)_1$	+5	856.7377	856.7374
$Pd_6(C_{15}H_{12}N_4)_{12}(BF_4)_7(PF_6)_1$	+4	1092.6732	1092.6719
$Pd_6(C_{15}H_{12}N_4)_{12}(BF_4)_8(PF_6)_1$	+3	1485.5657	1485.5643
$Pd_6(C_{15}H_{12}N_4)_{12}(BF_4)_4(PF_6)_2$	+6	709.1057	709.1069
$Pd_6(C_{15}H_{12}N_4)_{12}(BF_4)_5(PF_6)_2$	+5	868.3298	868.3298
$Pd_6(C_{15}H_{12}N_4)_{12}(BF_4)_6(PF_6)_2$	+4	1107.1634	1107.1627
$Pd_6(C_{15}H_{12}N_4)_{12}(BF_4)_7(PF_6)_2$	+3	1505.2192	1505.2178
$Pd_6(C_{15}H_{12}N_4)_{12}(BF_4)_4(PF_6)_3$	+5	879.9220	879.9222
$Pd_6(C_{15}H_{12}N_4)_{12}(BF_4)_5(PF_6)_3$	+4	1121.6521	1121.6535
$Pd_6(C_{15}H_{12}N_4)_{12}(BF_4)_6(PF_6)_3$	+3	1524.5394	1524.5369

Supplementary Table 6 The detailed MS assignment for all the Pd_nL_{2n} complexes observed in Supplementary Figure 70

formula	z	m/z(Simulated)	m/z(Observed)
$Pd_3(C_{15}H_{12}N_4)_6(NO_3)3$	+3	664.7714	664.7719
$Pd_3(C_{15}H_{12}N_4)_6(NO_3)_3(PF_6)$	+2	1069.6395	1069.6399
$Pd_4(C_{15}H_{12}N_4)_8(NO_3)_2(PF_6)$	+5	536.0815	536.0811
$Pd_4(C_{15}H_{12}N_4)_8(NO_3)_2$ $(PF_6)_2$	+4	706.3430	706.3440
$Pd_4(C_{15}H_{12}N_4)_8(NO_3)_2$ $(PF_6)_3$	+3	990.1123	990.1112
$Pd_4(C_{15}H_{12}N_4)_8(NO_3)_2$ $(PF_6)_4$	+2	1557.6507	1557.6496
$Pd_5(C_{15}H_{12}N_4)_{10}(NO_3)_2$ $(PF_6)_2$	+6	571.4148	571.4142
$Pd_5(C_{15}H_{12}N_4)_{10}(NO_3)_2$ $(PF_6)_3$	+5	714.6907	714.6913
$Pd_5(C_{15}H_{12}N_4)_{10}(NO_3)_2$ $(PF_6)_4$	+4	929.6045	929.6041
$Pd_5(C_{15}H_{12}N_4)_{10}(NO_3)_2$ $(PF_6)_5$	+3	1287.7942	1287.7931
$Pd_6(C_{15}H_{12}N_4)_{12}(NO_3)_2$ $(PF_6)_2$	+8	503.9506	503.9494
$Pd_6(C_{15}H_{12}N_4)_{12}(NO_3)_2$ $(PF_6)_3$	+7	596.6528	596.6524
$Pd_6(C_{15}H_{12}N_4)_{12}(NO_3)_2$ $(PF_6)_4$	+6	720.2558	720.2566
$Pd_6(C_{15}H_{12}N_4)_{12}(NO_3)_2$ $(PF_6)_5$	+5	893.2999	893.2992
$Pd_6(C_{15}H_{12}N_4)_{12}(NO_3)_2$ $(PF_6)_6$	+4	1152.866	1152.8651
$Pd_6(C_{15}H_{12}N_4)_{12}(NO_3)_2$ $(PF_6)_7$	+3	1585.4763	1585.4752
$Pd_6(C_{15}H_{12}N_4)_{12}(NO_3)_3(PF_6)_2$	+7	584.7991	584.7989
$Pd_6(C_{15}H_{12}N_4)_{12}(NO_3)_3(PF_6)_3$	+6	706.4264	706.4270
$Pd_6(C_{15}H_{12}N_4)_{12}(NO_3)_3$ $(PF_6)_4$	+5	876.7046	876.7045
$Pd_6(C_{15}H_{12}N_4)_{12}(NO_3)_3$ $(PF_6)_5$	+4	1132.3720	1132.3716
$Pd_6(C_{15}H_{12}N_4)_{12}(NO_3)_3$ $(PF_6)_6$	+3	1557.8175	1557.8173
$Pd_7(C_{15}H_{12}N_4)_{14}(NO3)_2$ $(PF_6)_3$	+9	531.0763	531.0741
$Pd_7(C_{15}H_{12}N_4)_{14}(NO3)_2$ $(PF_6)_4$	+8	615.5814	615.5793
$Pd_7(C_{15}H_{12}N_4)_{14}(NO3)_2$ $(PF_6)_5$	+7	724.2308	724.2317
$Pd_7(C_{15}H_{12}N_4)_{14}(NO3)_2$ $(PF_6)_6$	+6	869.0968	869.0984
$Pd_7(C_{15}H_{12}N_4)_{14}(NO3)_2$ $(PF_6)_7$	+5	1071.9091	1071.9077
$Pd_7(C_{15}H_{12}N_4)_{14}(NO3)_2$ $(PF_6)_8$	+4	1376.3776	1376.3796
